# A robust *cis*-regulatory network ensures *Otx2* expression during retinal development

**DOI:** 10.1242/dev.204881

**Published:** 2026-03-27

**Authors:** Ian J. Purvis, Omar E. Ochoa Olmos, Ko Uoon Park, Michael L. Kaufman, Charles M. Henry, Chris Schaaf, Olivia J. Clise, Corey D. Tesdahl, Ami Haas, Joseph A. Brzezinski

**Affiliations:** ^1^ Department of Ophthalmology, University of Colorado Anschutz Medical Campus, Aurora, CO 80045, USA; ^2^ Cell Biology, Stem Cells, and Development Graduate Program, University of Colorado Anschutz Medical Campus, Aurora, CO 80045, USA; ^3^ Department of Biomedical Informatics, University of Colorado Anschutz Medical Campus, Aurora, CO 80045, USA; ^4^ University of Colorado Cancer Center, University of Colorado Anschutz Medical Campus, Aurora, Colorado 80045, USA; ^5^ Neurosciences Graduate Program, University of Colorado Anschutz Medical Campus, Aurora, CO 80045, USA

**Keywords:** Enhancers, Promoters, Mouse, *Otx2*, Retinal development, Cell fate specification, Photoreceptors, Bipolar cells, CRISPRi

## Abstract

The transcription factor *Otx2* is essential for photoreceptor and bipolar cell formation during retinal development. *Otx2* expression is complex and underlies multiple cell fate decisions during development. To understand how *Otx2* expression is regulated, we explored the activity and function of three of its enhancers (DHS2, DHS4 and DHS15). Enhancer reporter assays and lineage tracing show that DHS4 initiates *Otx2* expression while DHS2 and DHS15 maintain expression in photoreceptors. Matched CRISPR/Cas9 and CRISPR interference systems were used to mutate or epigenetically silence enhancers, respectively. CRISPR reduced OTX2 expression acutely, but failed to significantly alter cell fate choice over the long term. In contrast, CRISPR interference of these enhancers caused permanent OTX2 loss and corresponding cell fate changes. While these data suggest that each enhancer is needed for normal *Otx2* expression, it also highlights that the enhancers can interact and substitute for each other during development. This *cis*-regulatory element flexibility likely promotes *Otx2* expression robustness. Such robustness may enable complex genes, like *Otx2*, to resist environmental stressors and regulatory disruptions to promote reproducible developmental outcomes.

## Teaser

**Summary:** Disruption of Otx2 *cis*-regulatory element function in murine retinas demonstrates flexibility in how they regulate Otx2 expression, suggesting that normal development depends on such *cis*-regulatory robustness.

## INTRODUCTION

The retina detects light and relays photic information to the brain, enabling vision. The retina comprises one glial and six neuronal cell types, which must be generated in the correct proportions to enable proper vision. These seven cell types arise from a population of retinal progenitor cells (Turner and Cepko, 1987; Turner et al., 1990). Progenitors permanently exit the cell cycle (i.e. are born) throughout development to generate neurons and glia. Progenitors that exit the cell cycle early in development primarily give rise to ‘early born’ cell types, such as retinal ganglion, amacrine, horizontal, and cone photoreceptor cells. Subsequently, progenitors generate ‘late born’ cell types, including rod photoreceptors, bipolar cells and Müller glia (Carter-Dawson and LaVail, 1979; Young, 1985; Turner et al., 1990; La Vail et al., 1991; Rapaport et al., 2004). Complex gene regulatory networks control how progenitors select their cell type identity (i.e. fate specification).

The transcription factor *Otx2* is an essential component in multiple developmental contexts (Baas et al., 2000). It plays a key role in a gene regulatory network needed for the formation of ∼85% of retinal cells. Conditional *Otx2* knockout from the developing mouse retina results in the absence of photoreceptors and bipolar cells, and an increase in amacrine and other cell types (Nishida et al., 2003; Koike et al., 2007; Sato et al., 2007; Yamamoto et al., 2020). During mouse retinal development, *Otx2* expression is activated in retinal progenitor cells just before exiting the cell cycle (Emerson et al., 2013; Ghinia Tegla et al., 2020; Muranishi et al., 2011; Kaufman et al., 2019). OTX2^+^ cells can give rise to five neuronal cell types: amacrine, horizontal and bipolar interneurons, and both cone and rod photoreceptors (Fossat et al., 2007; Sato et al., 2007; Baas et al., 2000; Brzezinski et al., 2013; Emerson et al., 2013; Nishida et al., 2003; Kaufman et al., 2021). Amacrine and horizontal cells lose *Otx2* expression during their maturation while photoreceptors and bipolar cells maintain expression into maturity, albeit at distinctly different levels (Koike et al., 2007; Nishida et al., 2003; Yamamoto et al., 2020; Fossat et al., 2007). The mechanisms that initiate and maintain this complex *Otx2* expression pattern in developing and mature cell types are not fully understood.

Transcription is controlled in large part by non-coding *cis*-regulatory elements, such as promoters and enhancers. Promoters are located just upstream of the transcriptional start site of a gene and are needed to recruit core transcriptional machinery to activate expression. Notably, *Otx2* has three putative promoters (1A, 1B and 1C) that result in transcripts with unique 5′ untranslated regions (Courtois et al., 2003; Fossat et al., 2005). In the embryonic eye, all three promoters are used with similar frequency, while the adult retina favors the two most distal promoters (A and B) (Fossat et al., 2005). The necessity and cell type-specific utilization of these *Otx2* promoters have not been determined within the retina. Enhancers are frequently located far from the transcription start site of a gene, at times exceeding 1 million base pairs (bp) from their target gene (Lim and Levine, 2021; Kim and Wysocka, 2023; Laverre et al., 2022). Unlike promoters, enhancers can be upstream of, downstream, or within genes. A prevailing model is that spatial, temporal and quantitative aspects of transcription are regulated by DNA looping that enables close interactions between promoters and enhancers in 3D space (Kim and Wysocka, 2023; Lim and Levine, 2021; Vos et al., 2021; Lin et al., 2022; Chen et al., 2024; Fulco et al., 2019; Hamamoto and Fukaya, 2022).

We previously identified three *Otx2* enhancers based on DNase hypersensitivity sequencing (DHS) of the developing retina, termed DHS2, DHS4 and DHS15 (Wilken et al., 2015). We found that the DHS4 enhancer controlled the initiation of *Otx2* expression (Kaufman et al., 2021). Although clustered regularly interspaced short palindromic repeats (CRISPR)/Cas9-mediated mutagenesis of DHS4 strongly decreased OTX2 during development, expression mostly recovered over time and only partially disrupted retinal cell fate specification (Kaufman et al., 2021). This suggested that other enhancers substitute for DHS4 to restore gene expression and the formation of OTX2^+^ photoreceptors and bipolar cells.

How DHS2 and DHS15 enhancers work with *Otx2* promoters to regulate gene expression and cell fate specification in the mouse retina is largely unknown. Here, we observed that DHS2 and DHS15 activity began in the embryonic retina and persisted into adulthood. Their expression patterns were very similar and restricted to OTX2^+^ cell types, including rods, cones and bipolar cells. Interestingly, systematic mutagenesis across each enhancer suggests that their activities are uniquely regulated. Targeting the DHS2 and DHS15 enhancers with CRISPR strongly reduced OTX2 expression. However, similar to DHS4 (Kaufman et al., 2021), OTX2 expression was restored at later time points. To test whether these enhancers were substituting for each other, we used CRISPR interference (CRISPRi) to perturb their function along with interacting enhancers. CRISPRi against each enhancer caused a permanent loss of OTX2 expression and a fate shift into OTX2-negative cell types. Our perturbation of the three *Otx2* promoters suggested that they were largely interchangeable during development. Together, our results argue that multiple *cis*-regulatory elements interact and substitute for each other to ensure robust *Otx2* expression and retinal cell fate specification. They also highlight the utility of directly comparing CRISPR and CRISPRi approaches to decipher enhancer function. Discovering how *Otx2* and other complex genes involved in multiple cellular contexts acquire regulatory flexibility and robustness is key to understanding the mechanisms of gene expression, development and evolution.

## RESULTS

### DHS2 and DHS15 drive activity in OTX2^+^ cells during retinal development

**Fig. 1.**
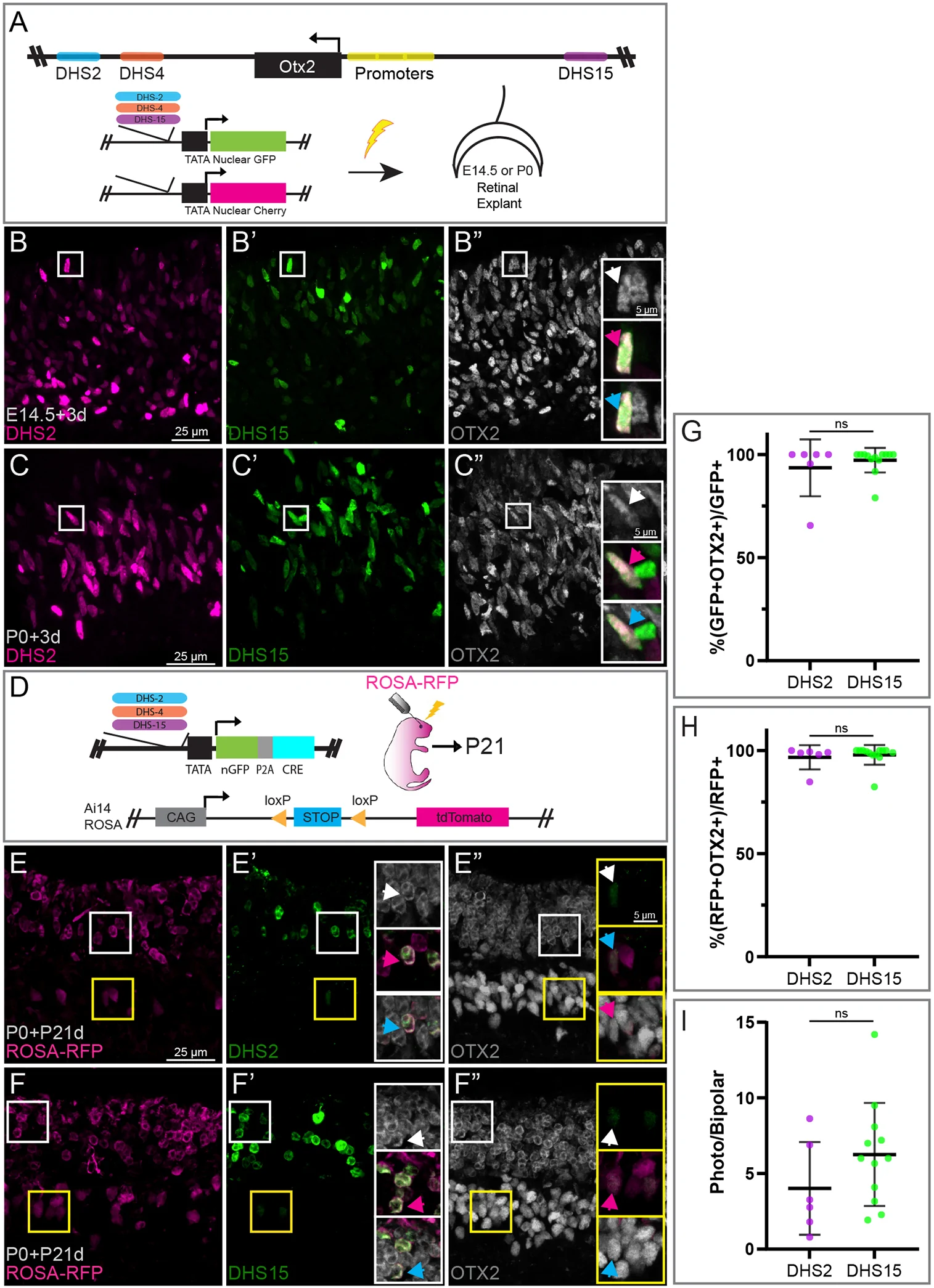
**Spatiotemporal characterization of *Otx2* enhancers.** (A) Enhancer cloning and electroporation scheme. Sequences were cloned upstream of a minimal TATA promoter to drive nuclear-localized (n) GFP or mCherry expression. E14.5 or P0 retinal explants were co-electroporated with a 1:1 mixture of plasmids and cultured *ex vivo* for 3 days. (B-C″) Immunohistochemical staining for GFP (DHS15), mCherry (DHS2) and OTX2. Boxed areas are magnified in insets. (D) Lineage tracing scheme. Enhancer sequences were cloned upstream of a minimal TATA promoter driving nGFP and Cre. Plasmids were electroporated *in vivo* into P0 *ROSA-RFP* mice to permanently label recombined cells with tdTomato. (E-F″) Staining for tdTomato, GFP and OTX2 in retinal sections from P21 eyes. Across this and subsequent figures, the outer retina is up and arrows mark single (white), double (magenta) or triple (cyan) labeled cells. Note that the white arrow in the yellow inset box of E″ marks a faintly GFP^+^ bipolar cell. Scale bars: 25 µm (main panels); 5 µm (insets). (G,H) Graphs showing the percentage of GFP^+^ (G) or tdTomato^+^ (RFP) (H) cells that co-express OTX2. (I) Graph showing the ratio of tdTomato^+^ (RFP) photoreceptors (OTX2 dim) to bipolar cells (OTX2 bright) in the lineage-tracing experiments. For plots, dots represent individual quantified images from a biological *N*≥3 explants. Bars show mean±sd. ns, not significant (Mann–Whitney non-parametric tests between groups; *P*>0.05).

*Otx2* is transiently made by precursors that can adopt multiple cell identities (Emerson et al., 2013; Ghinia Tegla et al., 2020; Muranishi et al., 2011; Kaufman et al., 2019, 2021; Fossat et al., 2007; Sato et al., 2007; Baas et al., 2000). Loss of *Otx2* causes a fate shift of photoreceptors and bipolar cells into other cell types (Nishida et al., 2003; Koike et al., 2007; Sato et al., 2007; Yamamoto et al., 2020; Kaufman et al., 2021; Ghinia Tegla et al., 2020). To better understand how *Otx2* controls fate specification, we previously used DHS to identify enhancers that regulate specific aspects of the *Otx2* expression pattern (Wilken et al., 2015). This revealed three conserved sequences, termed DHS2, DHS4 and DHS15, that flank the *Otx2* locus in mice (Fig. 1A, Fig. S1) (Wilken et al., 2015). Assay for transpose accessible chromatin sequencing (ATAC-seq) datasets generated from whole retina (Aldiri et al., 2017) show that DHS2 and DHS15 become more accessible as development proceeds (Fig. S1). Homologous human sequence regions have similar accessibility, suggesting functional conservation (Fig. S1) (Marchal et al., 2022; Finkbeiner et al., 2022). Each of the three DHS sequences were shown to have reporter activity in OTX2^+^ cells in the mouse retina and were presumed to function as *Otx2* enhancers (Wilken et al., 2015). We previously showed that DHS4 is transiently expressed at the onset of *Otx2* activation and is essential for normal OTX2 expression (Kaufman et al., 2021). Here, we explored DHS2 and DHS15 activity and function.

We first measured DHS2 and DHS15 activity using plasmid-based reporter assays in electroporated retinal explants. DHS2 and DHS15 sequences were cloned into reporter plasmids containing a minimal promoter driving nuclear-localized GFP or mCherry (Fig. 1A) (Mills et al., 2017; Goodson et al., 2020b; Kaufman et al., 2021; Wilken et al., 2015; Bachu et al., 2022). We have shown that these minimal promoter constructs drive negligible activity in the absence of upstream enhancer sequences (Goodson et al., 2020b; Mills et al., 2017; Bachu et al., 2022; Wilken et al., 2015). Constructs were electroporated into wild-type embryonic day (E) 14.5 or postnatal day (P) 0 mouse retinal explants and incubated for 3 days in culture (*ex vivo*) (Fig. 1A) (Mills et al., 2017; Goodson et al., 2020b; Kaufman et al., 2021; Bachu et al., 2022). The explants were fixed and subjected to immunohistochemistry to detect electroporated cells (GFP or mCherry) and OTX2. We observed that DHS2 and DHS15 were active at both embryonic and postnatal time points (Fig. 1B-C″, Fig. S2). Aligning with our previous observations (Wilken et al., 2015), DHS2 and DHS15 activities were restricted to OTX2^+^ cells, consistent with their roles as retinal *Otx2* enhancers. We then compared their activity patterns relative to each other in co-electroporated explants and observed many co-expressing cells (Fig. 1B-C″). To estimate the co-expression rate of these enhancers, we first calculated how often we could detect co-electroporated cells. We co-electroporated DHS2 (or DHS15) constructs that drove GFP and mCherry and calculated the double-labeling frequency (Fig. S2). We then divided the DHS2^+^/DHS15^+^ overlap by the maximum co-electroporation rate (61%) to conservatively estimate the overlap between these enhancers in embryonic (80%, *n*=3) and postnatal (68%, *n*=4) OTX2^+^ cells (Fig. S2). Since DHS4 initiates *Otx2* expression (Kaufman et al., 2021), we reasoned that DHS4^+^ cells would transition towards using DHS2 and DHS15 enhancers to maintain OTX2 expression. To test this, we co-electroporated the DHS4 reporter (Kaufman et al., 2021) along with DHS2 or DHS15 constructs into E14.5 or P0 explants (Fig. S3). After 3 days, we observed numerous cells that co-expressed DHS4 and either the DHS2 or DHS15 reporter (Fig. S3). These findings show that DHS2, DHS4 and DHS15 enhancers have transcriptional activity within the same OTX2^+^ cell, although not necessarily simultaneously.

Despite their high similarity, the DHS2 and DHS15 enhancer patterns were not identical, reflecting subtle differences in their onset timing, spatial patterns, or our ability to fully detect their activity. To dissect their patterns further, we conducted *in vivo* lineage-tracing experiments by inserting the DHS2 and DHS15 sequences into a plasmid that drives both nuclear GFP and Cre recombinase (Goodson et al., 2020b; Kaufman et al., 2021; Bachu et al., 2022) (Fig. 1D). These plasmids were electroporated *in vivo* into newborn *ROSA-RFP* (Goodson et al., 2020b; Kaufman et al., 2021; Madisen et al., 2010; Bachu et al., 2022) mouse eyes and their retinas were collected at P21, well after the completion of neurogenesis (Fig. 1D). In this system, cells that express Cre recombinase become permanently labeled by tdTomato (RFP) expression. In parallel, cells that have recently expressed the enhancer will be labeled with nuclear-localized GFP. We observed that both GFP^+^ and RFP^+^ cells in the DHS2 and DHS15 lineages were heavily biased (>95%) towards OTX2^+^ cell types (Fig. 1E-H). However, GFP signal was almost exclusively localized to photoreceptors (dim OTX2^+^) while RFP also marked bipolar cells (intense OTX2^+^) in the inner retina (Fig. 1E-F″). This pattern and the rare presence of GFP^+^ bipolar cells (Fig. 1E-F″) suggests that DHS2 and DHS15 are active in OTX2^+^ cells that give rise to photoreceptors and bipolar cells, but this activity is subsequently lost in bipolar cells as the retina matures. We then calculated the ratio of RFP^+^ photoreceptors to bipolar cells (Fig. 1I). The theoretical ratio is ∼10:1 (Jeon et al., 1998), but we expected this to be lower because our assay excludes a sizeable population of rods generated before P0. We observed photoreceptor-to-bipolar ratios of ∼4:1 for DHS2 and ∼7:1 for DHS15 (Fig. 1I). These ratios approximate what is expected in the postnatal retina, suggesting that bipolar cells are well represented in each enhancer lineage. This was further supported by the lack of any overt morphological subtype bias in the RFP^+^ bipolar cells we observed. Although the DHS2 and DHS15 ratios were not significantly different from each other, these enhancers may differ modestly in their onset timing or duration while photoreceptor and bipolar cells fate choices are being made.

To determine whether DHS2 and DHS15 label cone photoreceptors, we conducted lineage-tracing experiments in embryonic *ROSA-RFP* retinal explants. We electroporated our lineage plasmids at E14.5, collected them after 14 days of culture, and stained them for GFP, RFP, OTX2 and the cone markers S-opsin and ARR3 (Zhang et al., 2003; Applebury et al., 2000). As before, nearly all GFP^+^ and RFP^+^ cells co-expressed OTX2. We observed GFP^+^ and RFP^+^ cells that co-expressed S-opsin or ARR3 in the DHS2 and DHS15 electroporations, representing ∼6% of the lineage (Fig. S4). Thus, the DHS2 and DHS15 enhancers are also active in cones.

### Mutagenesis of enhancer sequences reveals potential transcription factor-binding sites

To predict which transcription factors bind and regulate DHS2 and DHS15, we first built and screened a series of enhancer sub-element constructs for their activity (Mills et al., 2017; Kaufman et al., 2021; Bachu et al., 2022). The full sequences of DHS2 (739 bp) and DHS15 (1232 bp) were subdivided and cloned into reporter vectors (Fig. 1A, Fig. S5). To measure electroporation efficiency, we used a positive-control plasmid containing a ubiquitously expressed EF1α (*EEF1A1*) promoter to drive nuclear-localized mCherry expression (Kaufman et al., 2021; Mills et al., 2017; Bachu et al., 2022; Wilken et al., 2015). Experimental and control plasmids were co-electroporated into P0 explants, cultured for 3 days, and evaluated for GFP, mCherry and OTX2 expression. DHS2 and DHS15 enhancer sub-element activity patterns were categorized as: (1) expressed and OTX2 specific, or (2) lacking activity and/or specificity (Fig. S5). From this, we identified short sequences that retained high GFP expression and OTX2 specificity for DHS2 (bit 10, 120 bp) and DHS15 (bit 6, 123 bp) (Fig. S5).

**Fig. 2.**
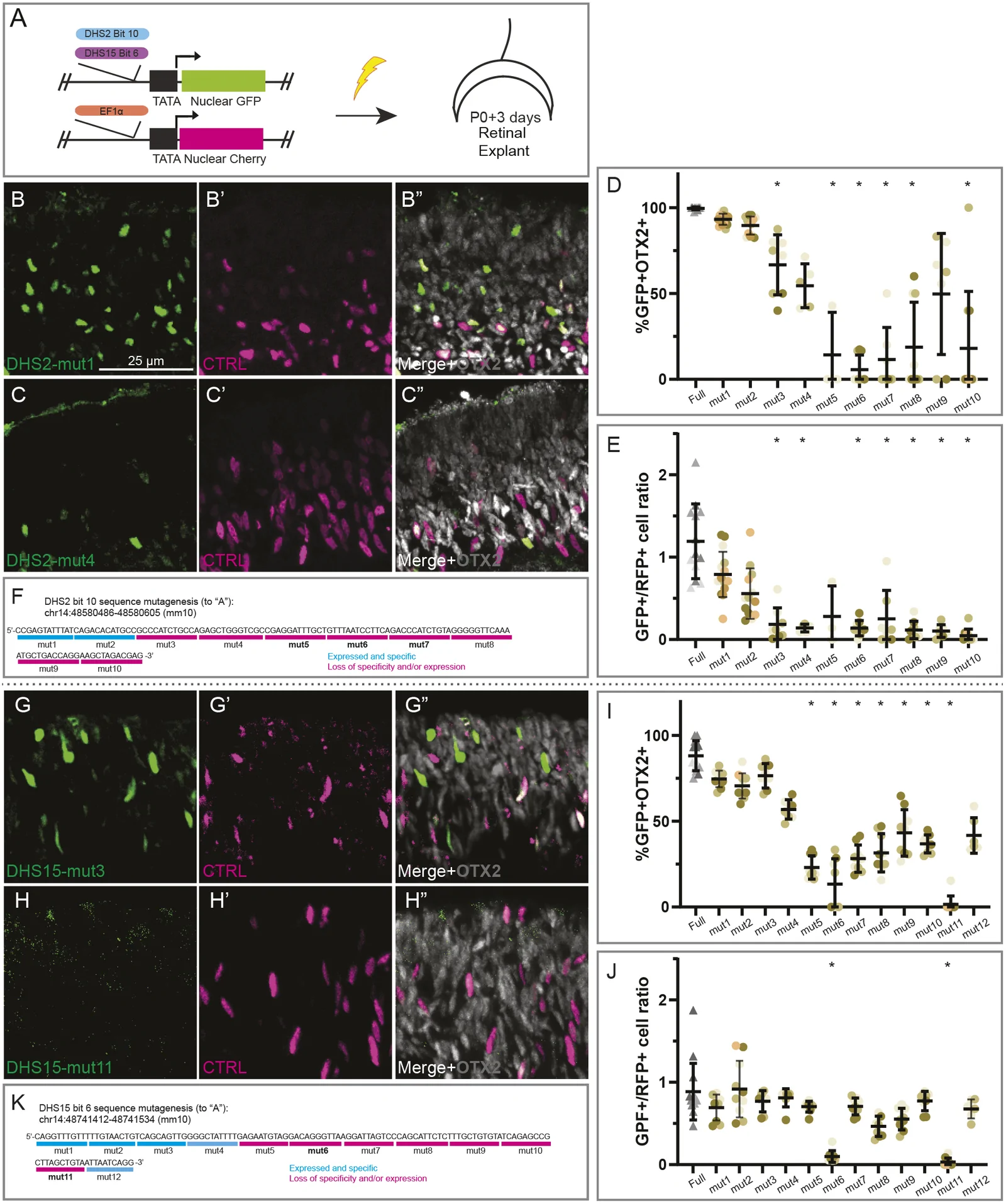
**Mutagenesis reveals essential sequences of DHS2 and DHS15.** (A) Scheme showing minimal DHS2 and DHS15 sub-elements used for tiled mutagenesis along with a ubiquitous electroporation control (EF1α-mCherry). Mutations replaced sequences with adenine (‘A’) nucleotides. Plasmids were co-electroporated in a 1:1 ratio in P0 explants and collected after 3 days of culture. (B-C″) Images showing GFP, mCherry (RFP) and OTX2 staining from DHS2 mutated sequences that retained (mut1; B-B″) and lost (mut4; C-C″) GFP expression. (D,E) Plots showing the percentage of GFP^+^ cells (specificity) and the ratio of GFP^+^ to RFP^+^ cells (activity) that express OTX2 in DHS2 sub-element constructs. The intact DHS2 minimal element (full) was used to establish a baseline. (F) Scheme showing mutagenized sequences within the DHS2 minimal element and whether they are necessary for specificity or activity. Bold denotes particularly important sequences. (G-H″) Images showing GFP, mCherry (RFP) and OTX2 expression from DHS15 mutated sequences that retained (mut3; B-B″) and lost (mut11; C-C″) GFP expression. (I,J) Plots for DHS15 specificity (I) and activity (J), as above. (K) Scheme showing essential DHS15 sequences, as above. Scale bars: 25 µm. Across this and subsequent figures, the dots and triangles in each plot represent individual quantified images from a biological *N*≥3 explants. Biological samples share the same color shade per condition. For this and subsequent figures, changes were evaluated using Kruskal–Wallis non-parametric ANOVA tests and the bars show mean±s.d. Black asterisks denote *P*<0.05 compared to the intact (full) condition.

To narrow the essential sequences further, we then systematically mutagenized DHS2 bit 10 and DHS15 bit 6∼10 bp at a time into strings of adenine (A) residues, generating 10-12 mutant (mut) constructs for each enhancer (Fig. 2). These constructs were electroporated into P0 explants and screened as above (Fig. 2). We quantified the percentage of GFP^+^ cells that expressed OTX2 (i.e. specificity) along with the ratio of GFP^+^ to RFP^+^ cells (i.e. activity) for each mutagenesis condition and compared them to the intact enhancers. DHS2 mutagenesis experiments demonstrated that the sequence regions covering mut5-8 were particularly important for specificity (Fig. 2B-F). The regions covered by mut3-10 had few GFP^+^ cells per section, showing that these sequences were essential for activity (Fig. 2B-F). Thus, critical transcription factor-binding sites span the mut3-10 regions of DHS2 (Fig. 2F). Mutagenesis of DHS15 yielded similar results (Fig. 2G-K). There was a loss of specificity with regions covered by mut4-12; however, conspicuous reductions in mut4 and mut12 specificity were not statistically significant (Fig. 2G-I). Activity was only reduced in mut6 and mut11 conditions (Fig. 2J). Together, these data suggest that the sequence spanning mut5-11 is essential for DHS15 bit 6 activity (Fig. 2G-J).

With essential sequences defined for each enhancer, we next predicted transcription factor-binding sites for DHS2 bit 10 and DHS15 bit 6 using JASPAR (Rauluseviciute et al., 2024). We identified many potential binding sites using a minimum score of 250 (*P*<0.007) (Table S1). We simplified this into transcription factor families and overlaid the results with the essential sequences for DHS2 (Fig. S6) and DHS15 (Fig. S7). While both essential regions had predicted K50 homeodomain (e.g. CRX and OTX2) and ROR (e.g. RORβ) binding sites, these similarly expressed enhancers had many non-concordant predictions (Figs S6, S7). For example, there were predictions for basic helix-loop-helix (bHLH) factors (e.g. NEUROD1) for DHS2, but MEF2 and Q50 homeodomain (e.g. RAX) for DHS15 (Figs S6-S7). These data suggest that DHS2 and DHS15 use both shared and distinct upstream regulators to drive highly similar spatial and temporal activity patterns in the retina.

### Targeted mutagenesis of DHS2 and DHS15 reduces OTX2 acutely

**Fig. 3.**
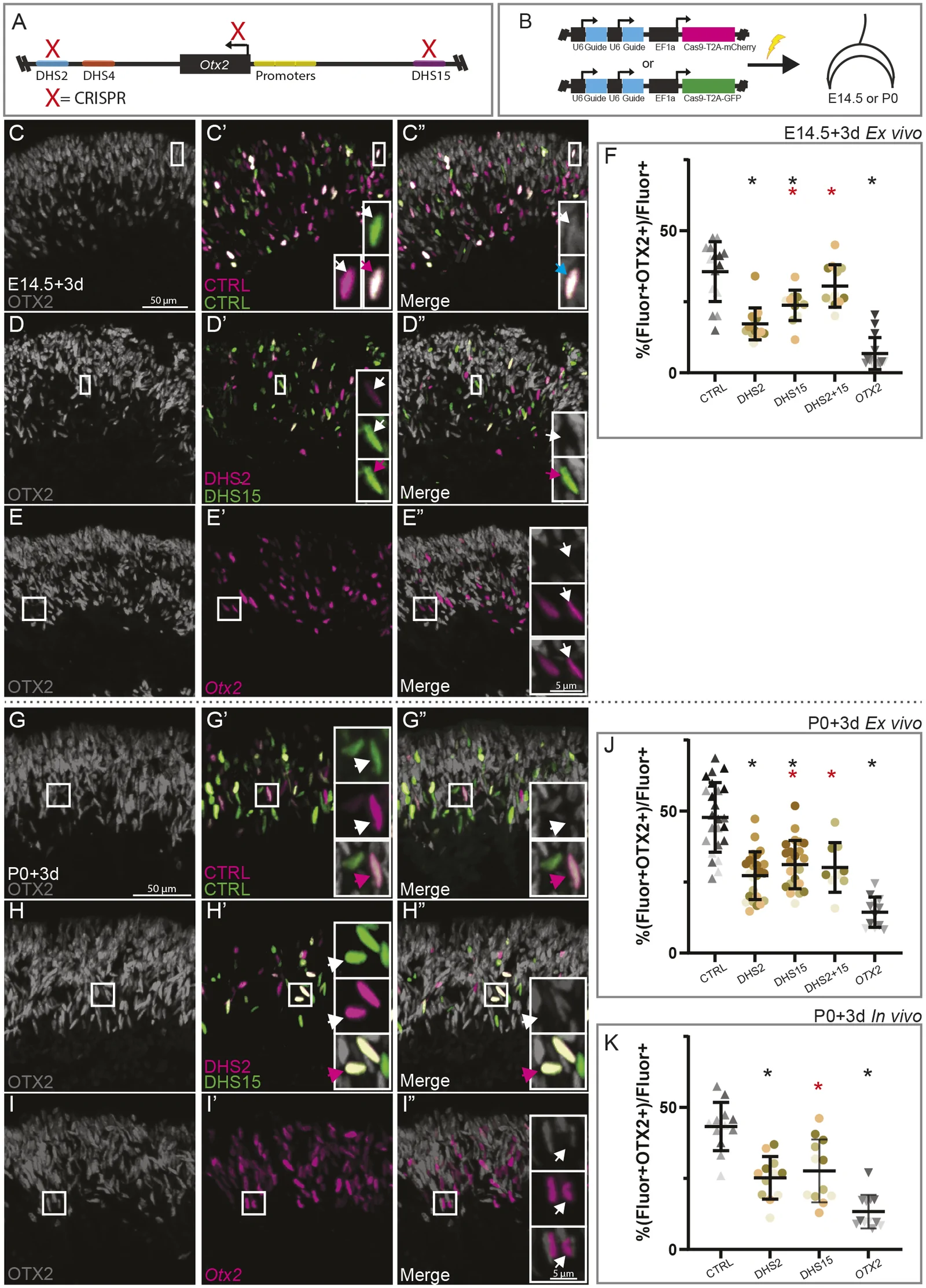
**CRISPR targeting of DHS2 and DHS15 reduces OTX2.** (A) Scheme showing conventional CRISPR (X) targeting of enhancers and the *Otx2* coding region (positive control). (B) Scheme showing all-in-one, dual guide plasmid structure. The ubiquitous EF1α promoter drives Cas9-T2A-H2B-mCherry (or GFP) expression. Negative control plasmids contain guides that do not target the mouse genome. Plasmids were electroporated into E14.5 or P0 retinal explants (*ex vivo*) or eyes (*in vivo*). (C-E″,G-I″) Retinas stained for OTX2, mCherry and GFP. (C-E″) E14.5 electroporated explants collected after 3 days. Boxed areas are magnified in insets. Scale bars: 50 µm (main panels); 5 µm (insets). (F) Graph showing the percentage of electroporated cells that express OTX2 under negative control (CTRL), single CRISPR (DHS2 or DHS15), dual CRISPR (DHS2+15) and positive control conditions (OTX2). (G-I″) Images from explants electroporated at P0 and cultured for 3 days. (J,K) Graphs showing the percentage of electroporated cells that express OTX2 from P0 retinas electroporated and cultured *ex vivo* (J) or *in vivo* collected at P3 (K). Black asterisks denote *P*<0.05 compared to the negative control (CTRL) condition while red asterisks denote *P*<0.05 compared to the positive control (OTX2).

DHS2 and DHS15 activity is highly specific to OTX2^+^ retinal cell types, suggesting that these enhancers are essential. To test this, we took a CRISPR mutagenesis approach, as done previously (Kaufman et al., 2021; Goodson et al., 2020a), to target essential sequences in DHS2 and DHS15 (Fig. 2). Pairs of CRISPR guides targeting the DHS2 bit 10 and DHS15 bit 6 regions were designed and cloned into a plasmid system that uses two U6 promoters to express guide RNA pairs along with a ubiquitously expressed EF1α element to drive Cas9 and a nuclear-localized fluorescent reporter (Fig. 3, Table S2) (Goodson et al., 2020b; Kaufman et al., 2021). This all-in-one system allowed us to electroporate a single plasmid to perform targeted mutagenesis while tracking the affected cells with a nuclear reporter (Fig. 3). As a positive control, we built a CRISPR plasmid containing two guides targeting the first coding exon of *Otx2.* Our negative, non-targeting control contained two guides that lack binding sites in the mouse genome (Kaufman et al., 2021; Goodson et al., 2020a). Plasmids targeting DHS2 or DHS15 were electroporated into E14.5 or P0 retinal explants and grown for 3 days in culture before being stained for the fluorescent reporter (GFP or mCherry) and OTX2 (Fig. 3C-J). The percentage of electroporated cells that co-expressed OTX2 was quantified for each time point and compared against non-targeting and *Otx2* targeting control conditions. Targeting DHS2 or DHS15 at both E14.5 and P0 showed significantly reduced overlap between electroporated cells and OTX2 compared to the negative control (Fig. 3F,J, black asterisks). The reduction from DHS15 targeting was not as severe as perturbing the *Otx2* coding region at either time point (Fig. 3F,J, red asterisks). Since DHS2 and DHS15 have similar expression patterns, we tested whether these enhancers were redundant by targeting both simultaneously. For this, we co-electroporated CRISPR constructs for each enhancer and examined GFP^+^/RFP^+^ double-labeled cells (Fig. 3D-D″,H-H″). Simultaneous targeting did not reduce OTX2 expression more than single perturbations, arguing against redundancy at these time points (Fig. 3F,J).

To account for developmental differences when culturing retinal explants, *in vivo* electroporations were conducted in newborn eyes and the retinas were collected at P3. After immunohistochemistry and quantification as described above, we observed similar OTX2 overlap reductions in DHS2 and DHS15 targeting conditions, but with more variability (Fig. 3K). Together, our *in vivo* and *ex vivo* results argue that DHS2 and DHS15 enhancers are essential for OTX2 expression at these developmental time points.

### Targeting DHS2 and DHS15 enhancers has a moderate effect on cell fate choice

**Fig. 4.**
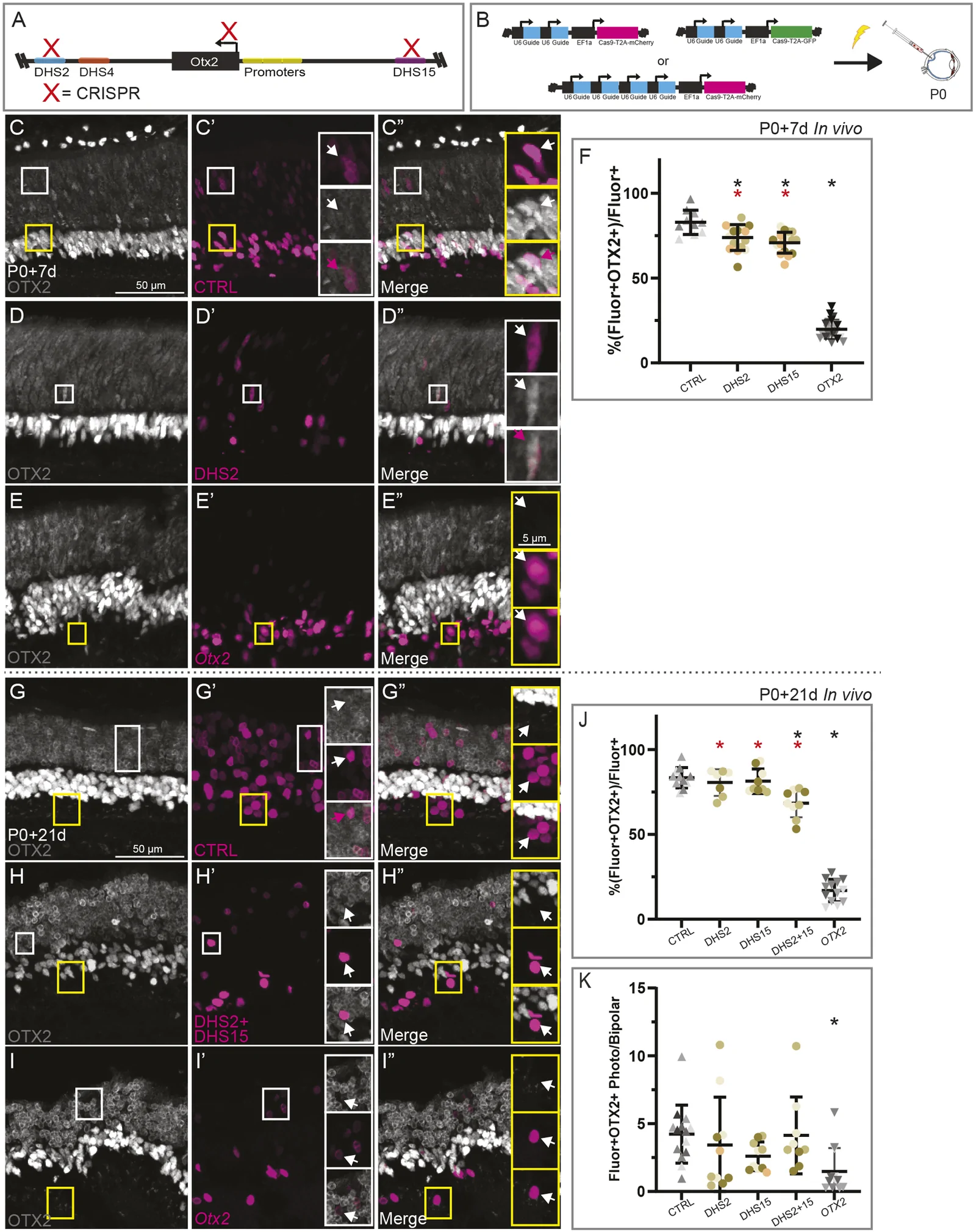
**DHS2 and DHS15 CRISPR modestly affects cell fate choice.** (A-B) Scheme showing CRISPR targets (X) and plasmid design, as in Fig. 3. Plasmids were electroporated *in vivo* into P0 retinas and collected at P7 (C-E″) or P21 (G-I″). (C-E″,G-I″) Staining for mCherry (or GFP) and OTX2 in P7 (C-E″) or P21 (G-I″) retinas. Note that EF1α-driven plasmids often show dimmer signal intensity in photoreceptors at these ages, similar to the OTX2 staining pattern. Boxed areas are magnified in insets. Scale bars: 50 µm (main panels); 5 µm (insets). (F,J) Graphs showing the percentage of electroporated cells that co-express OTX2 under negative control (CTRL), single CRISPR (DHS2 or DHS15) and positive control conditions (OTX2) at P7 (F) or P21 (J). The P21 time point includes the four guide RNA plasmid used to simultaneously target both enhancers (DHS2+15). (K) Graph showing the photoreceptor-to-bipolar (OTX2 dim/bright) cell ratio of electroporated OTX2^+^ cells at P21. Black asterisks denote *P*<0.05 compared to the negative control (CTRL) condition while red asterisks denote *P*<0.05 compared to the positive control (OTX2).

Disrupting DHS2 and DHS15 strongly reduced OTX2 expression after 3 days, suggesting that these enhancers are necessary for normal cell fate specification. To test this, we repeated DHS2 and DHS15 CRISPR perturbations using P0 *in vivo* electroporation. However, we collected retinas at P7 and P21 to measure cell fate changes after the completion of neurogenesis (Fig. 4) (Carter-Dawson and LaVail, 1979; Rapaport et al., 2004; Young, 1985). We stained retinas for electroporated cells (GFP or mCherry) and OTX2. At P7 (Fig. 4C-F), the percentage of electroporated cells that co-expressed OTX2 was strongly and significantly reduced in the *Otx2* targeting condition compared to the non-targeting negative control (Fig. 4F, black asterisk). Targeting DHS2 and DHS15 significantly reduced OTX2 overlap (∼15%), but this effect was modest compared to P0 retinas examined after 3 days (∼50%) and the positive control condition (∼75%) (Figs 3K and 4F). These modest phenotypes at P7 are unlikely due to CRISPR dilution from additional cell divisions, as the positive control plasmids targeting the *Otx2* coding region remain highly efficacious at P7 and because we observed Cas9 protein in electroporated retinas at this and later time points (Fig. 4F, Fig. S8).

We then examined the effects of enhancer perturbations at P21, well after neurogenesis has completed (Fig. 4G-K). As at P7, targeting the *Otx2* coding region profoundly lowered OTX2 co-expression (Fig. 4I-J). However, DHS2 and DHS15 CRISPR targeting did not reduce OTX2 overlap compared to the negative control (Fig. 4J). Thus, mutating these enhancers did not grossly alter cell fate specification. We also observed that the photoreceptor-to-bipolar cell ratio was equivalent between negative control, DHS2 and DHS15 targeting conditions, arguing against a fate shift within OTX2^+^ cell types (Fig. 4K). Surprisingly, targeting the *Otx2* coding region showed a significant reduction in the photoreceptor-to-bipolar cell ratio, which may indicate a higher impact on photoreceptor formation (Fig. 4K).

We did not see complete loss of OTX2 when targeting the coding region, reflecting the incomplete penetrance of the CRISPR system at the individual cell level (Figs 3, 4) (Fuselier et al., 2021; Wang et al., 2024; Goodson et al., 2020a; Kaufman et al., 2021). Most photoreceptors in these conditions still make OTX2, demonstrating that they escaped accumulating homozygous deleterious mutations. However, we consistently observed a small number (five or fewer per imaged field) of electroporated cells in the outer nuclear layer at P21 that lacked OTX2 (Fig. 4I-I″). We stained these retinas with the rod marker NR2E3 (Haider et al., 2000, 2001; Bumsted O'Brien et al., 2004; Peng et al., 2005) and observed that these OTX2-negative cells were NR2E3^+^ (Fig. S9). This argues that, once formed, rods can tolerate *Otx2* loss and survive (Housset et al., 2013). We rarely observed OTX2 loss within photoreceptors in the DHS2 and DHS15 CRISPR targeting conditions at P7 or P21, suggesting that targeted cells recovered OTX2 expression. The lack of overt toxicity and cell fate changes within the OTX2 population argues against a strong selection effect from enhancer targeting. Together, these findings raised the possibility that OTX2 recovery is due to enhancer substitution.

To test whether DHS2 and DHS15 can substitute for each other, we targeted both enhancers simultaneously and examined P21 retinas. To increase efficiency, we retrofitted our CRISPR system to use a single plasmid expressing all four guides against these enhancers simultaneously (Figs 3, 4H, Fig. S8). Compared to the negative control, there was a significant reduction in OTX2 overlap in the double-targeting condition (Fig. 4J, black asterisk). However, this reduction was modest compared to the positive control (Fig. 4J, red asterisk). There was no change in the photoreceptor-to-bipolar cell ratio in the double-targeting condition (Fig. 4K). This reduction in OTX2^+^ cells suggested that another population is increased, presumably amacrine cells (Koike et al., 2007; Nishida et al., 2003; Yamamoto et al., 2020; Sato et al., 2007; Ghinia Tegla et al., 2020). To measure this, we stained retinas from each condition with PAX6, which marks amacrine cells intensely in the inner nuclear layer (Rath et al., 2009; Lakowski et al., 2007; de Melo et al., 2003). We observed no significant differences in the percentage of electroporated cells that expressed PAX6 between the negative control and the single DHS2 and DHS15 targeting conditions, as expected (Fig. S10). This contrasted with targeting the *Otx2* coding region, which increased PAX6 overlap (Fig. S10, black asterisk). Targeting DHS2 and DHS15 simultaneously with our four-guide plasmid significantly increased PAX6 overlap, consistent with a proportional shift from OTX2^+^ fates towards amacrine identity (Fig. 4J, Fig. S10). Taken together, our results suggest that DHS2 and DHS15 partially substitute for each other to enable OTX2 expression and photoreceptor and bipolar cell formation.

When designing our CRISPR guide pairs to target DHS2 and DHS15, we selected the highest scoring sequences that were within or closely flanking each element on the presumption that they would create guide spanning deletions that permanently disable enhancer function. To test this, we profiled CRISPR mutations in retinal cells. We electroporated P0 retinas and flow sorted fluorescent cells after 3 days of culture. We then purified DNA, generated short PCR amplicons spanning each guide, and subjected them to next generation sequencing. As controls, we electroporated non-targeting guides and positive control guides against the *Otx2* coding region. After aligning sequence reads, we were able to determine the types of mutations created by each guide, except for one from DHS15 (Table S3). Importantly, we found little evidence of deletions spanning guide pairs. Moderate (6-30 bp) deletions were also uncommon. Instead, we found that most indels and substitutions extended just one to five nucleotides from the predicted cutting site in each guide (Table S3). We also saw differences between different guides and target sites. For the coding region, one guide was much more effective at creating indels that disrupt the reading frame (Table S3). Notably, a large fraction of reads in our control coding region sample contained small indels (>25%) at each guide locus. This is consistent with sequence differences between electroporated CD1 mice and the mm39 C57BL/6J reference genome. While CRISPR at the *Otx2* coding region was highly effective at making disabling mutations, the observed rate (∼50%) was lower than the OTX2 loss observed by histology (∼75%). This argues that our mutation screening platform under-reports disabling mutations. Although we could not screen DHS15 fully, we observed few mutations in the enhancer region (Table S3). Both DHS15 guides are located just outside the critical sequence region; thus, small indels from either guide are unlikely to disable enhancer activity. DHS2 guides overlap with the essential mut3 and mut7 regions, but the one spanning mut7 was more effective at creating mutations. Nonetheless, the rate of potentially disabling mutations was still low (∼15%) (Table S3). This included a frequently observed 1 bp insertion that disrupts a highly conserved bHLH binding site in mut7 (Fig. S6, Table S3). Overall, our findings argue that mutagenesis of DHS2/15 enhancer sequences was not sufficient to cause the observed OTX2 expression loss.

Cas9 can persistently bind to intact and cut DNA (Richardson et al., 2016; Sternberg et al., 2014), and may sterically inhibit transcription factor localization to nearby essential binding sites to prevent enhancer activity. To test whether Cas9 blocks enhancer activity, we modified our DHS2 and DHS15 CRISPR plasmids to contain a mutated version of Cas9 that lacks cutting activity (dCas9) (Mali et al., 2013). These dCas9 constructs were electroporated into P0 explants, collected after 3 days of culture, and stained for dCas9 and OTX2 (Fig. S11). Targeting either DHS2 or DHS15 reduced OTX2 significantly, consistent with steric inhibition of enhancer function (Fig. S11). Nonetheless, the OTX2 reduction was roughly half as efficacious as conventional (cutting) CRISPR conditions (Fig. 3). This argues that the DHS2 and DHS15 CRISPR phenotypes are caused by a combination of mutagenesis and steric hinderance driven by persistent Cas9 binding.

### DHS2 and DHS15 CRISPR interference impairs enhancer function

We found that conventional CRISPR targeting of DHS2 and DHS15 strongly reduced the number of cells that express OTX2 after 3 days. However, this loss was subsequently overcome, enabling relatively normal cell fate specification. Owing to the small number of disabling mutations in DHS2 and DHS15, recovery is likely due to enhancer substitution. To examine this further, we took a CRISPRi approach. CRISPRi uses a catalytically dead (d) version of Cas9 fused to protein domains (KRAB and MeCP2) that epigenetically silence transcription without introducing DNA mutations (Yeo et al., 2018). CRISPRi-mediated silencing is only effective when targeting *cis*-regulatory sequences (e.g. promoters and enhancers) and the repressive effect does not spread far along the genomic DNA from the location targeted by guide RNAs (Yeo et al., 2018; Morris et al., 2023; Fulco et al., 2016; Kaplan et al., 2024; Lin et al., 2022; Usluer et al., 2023). Based on these features, we reasoned that CRISPRi could be used to permanently disrupt DHS2 and DHS15 enhancer function along with that of other closely interacting enhancers brought into tight proximity through DNA looping.

**Fig. 5.**
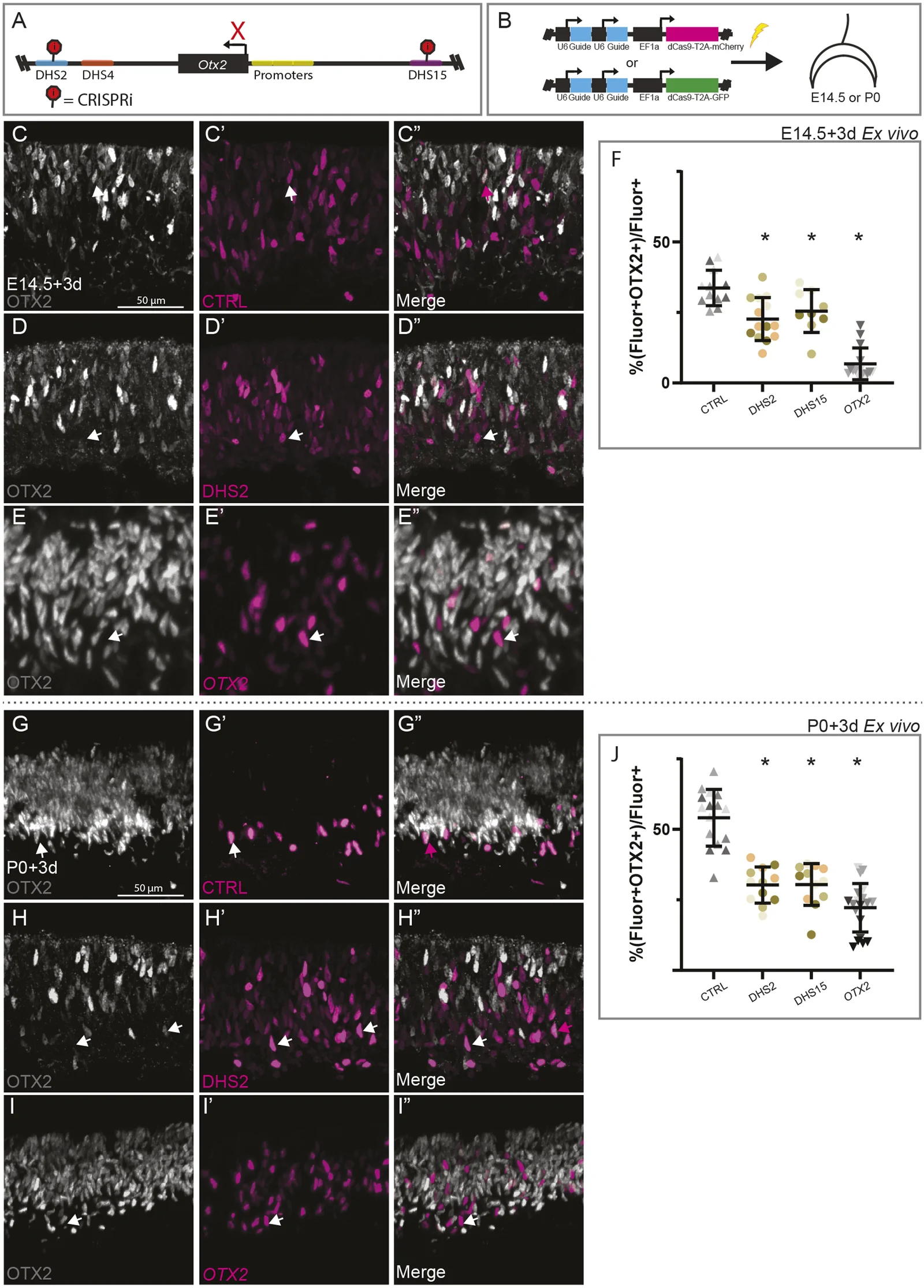
**DHS2 and DHS15 CRISPR interference reduces OTX2 expression.** (A) Scheme showing CRISPRi targets (stop sign) and the CRISPR positive control targeting the *Otx2* coding region (X). (B) Plasmids containing guide sequences used for conventional CRISPR in Fig. 3 upstream of a ubiquitous EF1α promoter driving Cas9-KRAB-MeCP2-T2A-H2B-mCherry (or GFP) expression. The negative control plasmid contains guides that do not target the mouse genome. Plasmids were electroporated into E14.5 or P0 retinal explants and cultured for 3 days. (C-E″,G-I″) Retinas stained for mCherry (or GFP) and OTX2 from E14.5 (C-E″) or P0 (G-I″) electroporations. Scale bars: 50 µm. (F,J) Graphs showing the percentage of electroporated cells that express OTX2 under negative control CRISPRi (CTRL), single CRISPRi (DHS2 or DHS15) and positive control conventional CRISPR conditions (OTX2) from E14.5 (F) or P0 (J) electroporations. Black asterisks denote *P*<0.05 compared to the negative control (CTRL) condition.

To perform CRISPRi, we retrofitted our all-in-one CRISPR plasmids to use a dCas9-KRAB-MeCP2 cassette. This was coupled with nuclear-localized fluorescent protein (GFP or mCherry) reporters. Plasmids contained two U6 promoters and the same guide RNA pairs were reused to target DHS2 and DHS15 (Fig. 5, Table S2). A negative control plasmid was built in the same fashion, again using guides that do not target the mouse genome. To test the efficacy of these CRISPRi plasmids, retinal explants were electroporated at E14.5 or P0 (Fig. 5) and incubated for 3 days in culture. The retinas were collected and stained for electroporated cells (GFP or mCherry) and OTX2. We observed that CRISPRi targeting of DHS2 and DHS15 strongly reduced OTX2 overlap at both time points compared to the negative control (Fig. 5C-J, black asterisks). This OTX2 reduction was not statistically different from what we observed when targeting the *Otx2* coding region using conventional CRISPR (Fig. 5E-F,I-J). This showed that DHS2 and DHS15 CRISPRi can be used to efficiently disrupt enhancer function.

### Targeting *Otx2* enhancers with CRISPRi alters cell fate

**Fig. 6.**
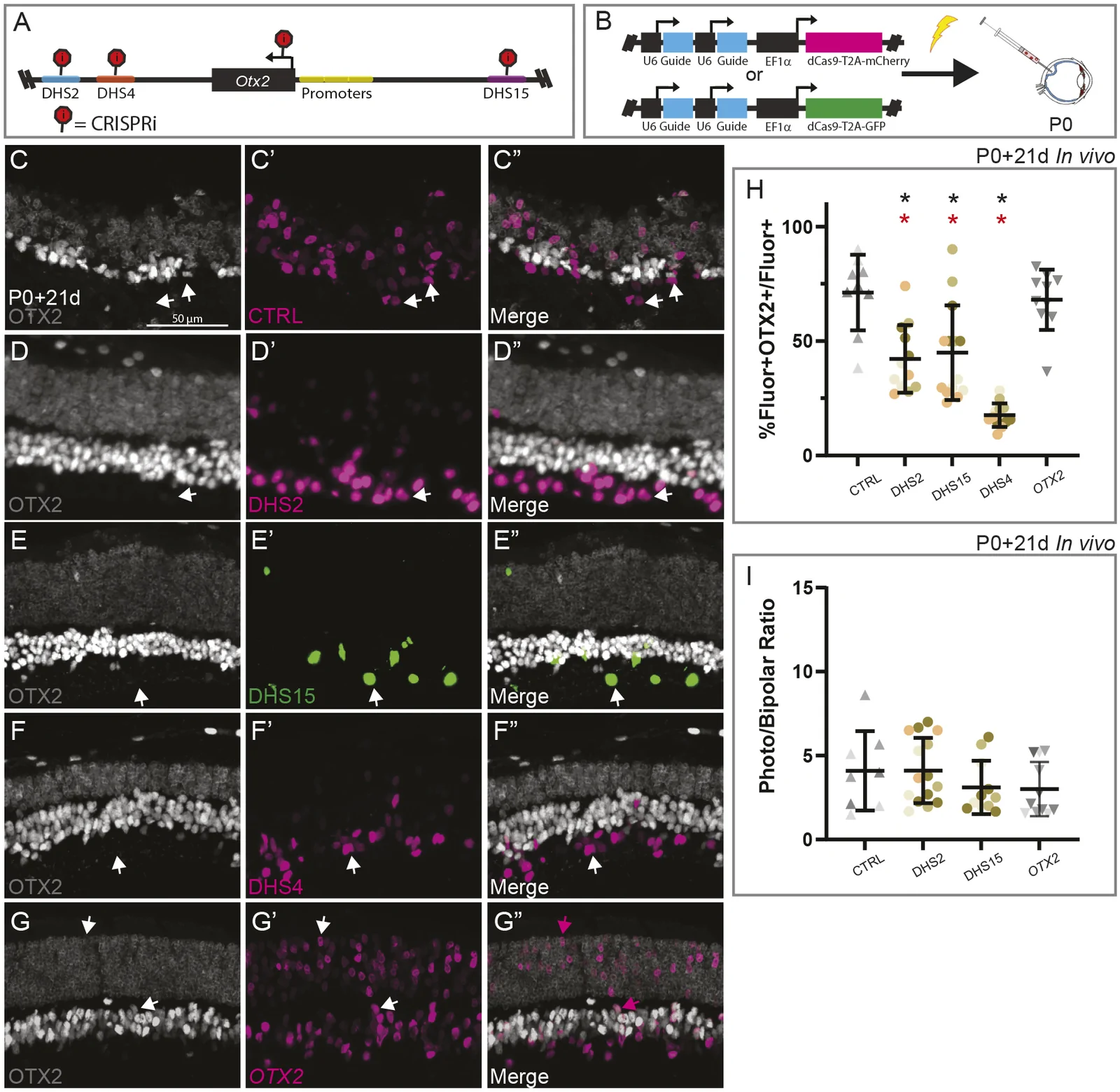
**DHS2, DHS4 and DHS15 CRISPRi alter retinal cell fate choice.** (A,B) Scheme showing CRISPRi targets (stop sign) and plasmid design, as in Fig. 5. A control CRISPRi plasmid targeting the *Otx2* coding region was used as a specificity control (OTX2). Plasmids were electroporated *in vivo* at P0 and eyes collected at P21. (C-G″) Retinal sections stained for mCherry (or GFP) and OTX2. Note that EF1α driven plasmids often show dimmer signal intensity in photoreceptors. Scale bars: 50 µm. (H) Graph showing the percentage of electroporated cells that express OTX2 under negative control (CTRL), single targeting (DHS2, DHS4 or DHS15), and specificity control (OTX2) CRISPRi conditions. (I) Graph showing the photoreceptor-to-bipolar (OTX2 dim/bright) cell ratio of electroporated OTX2^+^ cells at P21. Black asterisks denote *P*<0.05 compared to the negative control (CTRL) condition while red asterisks denote *P*<0.05 compared to the specificity control (OTX2).

We anticipated that targeting DHS2 and DHS15 with CRISPRi would permanently block their activity while preventing interactions with other enhancers. This would severely inhibit OTX2 expression, altering cell fate choice in the retina. To test this, we used DHS2, DHS4 and DHS15 CRISPRi constructs with the same guides used in our conventional CRISPR experiments to enable direct comparisons (Figs 3, 4, 6) (Kaufman et al., 2021). We used the same CRISPRi negative control as described above (Fig. 5). As a regional specificity control, we created a CRISPRi plasmid that targets the first coding exon of *Otx2.* This mimics our conventional CRISPR targeting conditions, but is not expected to interfere with transcription as the guides are located too far downstream of the *Otx2* promoters (Fig. 6). We performed *in vivo* electroporation of our CRISPRi constructs in newborn mice and collected retinas at P21. Tissues were stained for electroporated cells (GFP or mCherry) and OTX2 (Fig. 6C-G″). We observed that electroporated cells in the negative control conditions were mostly OTX2^+^ rods and bipolar cells (∼74%) (Fig. 6H). OTX2-negative amacrines and glia were seen in the inner nuclear layer (Fig. 6C). CRISPRi targeting of DHS2, DHS4 and DHS15 shifted the distribution of electroporated cells towards the inner nuclear layer and reduced overlap with OTX2 (Fig. 6D-F″). DHS4 CRISPRi resulted in the strongest reduction of OTX2, mimicking conventional CRISPR targeting of the *Otx2* coding region (Figs 4J and 6H). This perturbation was much stronger than we previously observed with DHS4 conventional CRISPR (Kaufman et al., 2021). In contrast, CRISPRi of DHS2 and DHS15 each reduced OTX2 overlap by ∼30% (Fig. 6H), which was roughly twice as efficacious as simultaneous conventional CRISPR targeting of these enhancers (Fig. 4J). No changes were seen in the photoreceptor-to-bipolar cell ratios compared to control (Fig. 6I), suggesting that targeting DHS2 and DHS15 impacts OTX2^+^ cell fates equally. These stronger cell fate phenotypes were consistent with CRISPRi permanently disrupting DHS2 and DHS15 activity along with interacting *cis*-regulatory elements. Finally, targeting the *Otx2* coding region with CRISPRi did not significantly reduce its own expression, reinforcing its need for proximity to relevant regulatory loci such as promoters and enhancers to silence gene expression (Fig. 6G-I).

CRISPRi has been estimated to operate over linear sequence regions spanning <500 bp (Yeo et al., 2018; Morris et al., 2023; Fulco et al., 2016; Kaplan et al., 2024; Lin et al., 2022; Usluer et al., 2023). To further evaluate the specificity of our CRISPRi system, we designed six guides targeting non-coding regions across the *Otx2* locus (Fig. S12). These plasmids were electroporated in E14.5 explants, cultured, and examined using the same paradigm mentioned above (Fig. 5). Of these constructs, five had no significant impact on OTX2 expression, whereas one unexpectedly increased OTX2 expression (Fig. S12). This experiment was repeated by *in vivo* electroporation of P0 retinas with three constructs. None of these guide sequences reduced OTX2 expression at P21 (Fig. S12). Rarely during our CRISPR and CRISPRi *in vivo* experiments, we electroporated the OTX2^+^ retinal pigmented epithelium (RPE) (Fig. S13). RPE electroporated with either DHS4 CRISPRi or DHS2 CRISPR always retained OTX2 expression (Fig. S13). Correspondingly, ATAC-seq data from mature mouse RPE shows that DHS2 and DHS4 lack appreciable chromatin accessibility (Fig. S13) (Liao et al., 2024). Together, these results demonstrate the tissue-specific nature of these *Otx2* enhancers and the narrow context-dependent conditions by which CRISPRi can alter gene expression and fate choice *in vivo*.

### Disrupting *Otx2* promoters suggests that they are interchangeable

The murine *Otx2* gene has at least three different transcriptional start sites, with promoters immediately upstream of non-coding exons 1A, 1B and 1C (Fig. S14) (Courtois et al., 2003; Fossat et al., 2005). These promoters are each utilized dynamically across retinal development (Fossat et al., 2005). To determine whether these promoters are necessary for OTX2 expression in the retina, we designed CRISPR and CRISPRi guide pairs targeting exons 1A, 1B and 1C (Figs S14, S15, Table S2). Owing to a lack of high scoring guides immediately upstream of these exons, we did not ideally target the promoters. Nonetheless, one guide of each pair was near (1A: +11 bp; 1B: +3 bp; 1C: +88 bp) the transcription start site of each exon, enabling CRISPRi efficacy. These constructs were electroporated into E14.5 or P0 retinal explants, cultured for 3 days, and evaluated by immunohistochemistry as above (Figs 3, 5). We observed that CRISPRi of exon 1A significantly reduced OTX2 expression in E14.5 explants, but slightly elevated OTX2 in P0 cultures (Fig. S14). Conventional CRISPR targeting of exons 1A, 1B and 1C with the same guide pairs did not alter OTX2 expression at either E14.5 or P0 (Fig. S15). However, it slightly and variably increased PAX6 expression (Fig. S15). We performed mutation screens for each promoter targeting guide and observed a range of mutations (Table S3). This included a higher frequency of medium (>10 bp) and guide-spanning deletions (Table S3). While further study is needed, the absence of major phenotypes using matched CRISPR and CRISPRi approaches suggests that the *Otx2* promoters are interchangeable during retinal development.

## DISCUSSION

**Fig. 7.**
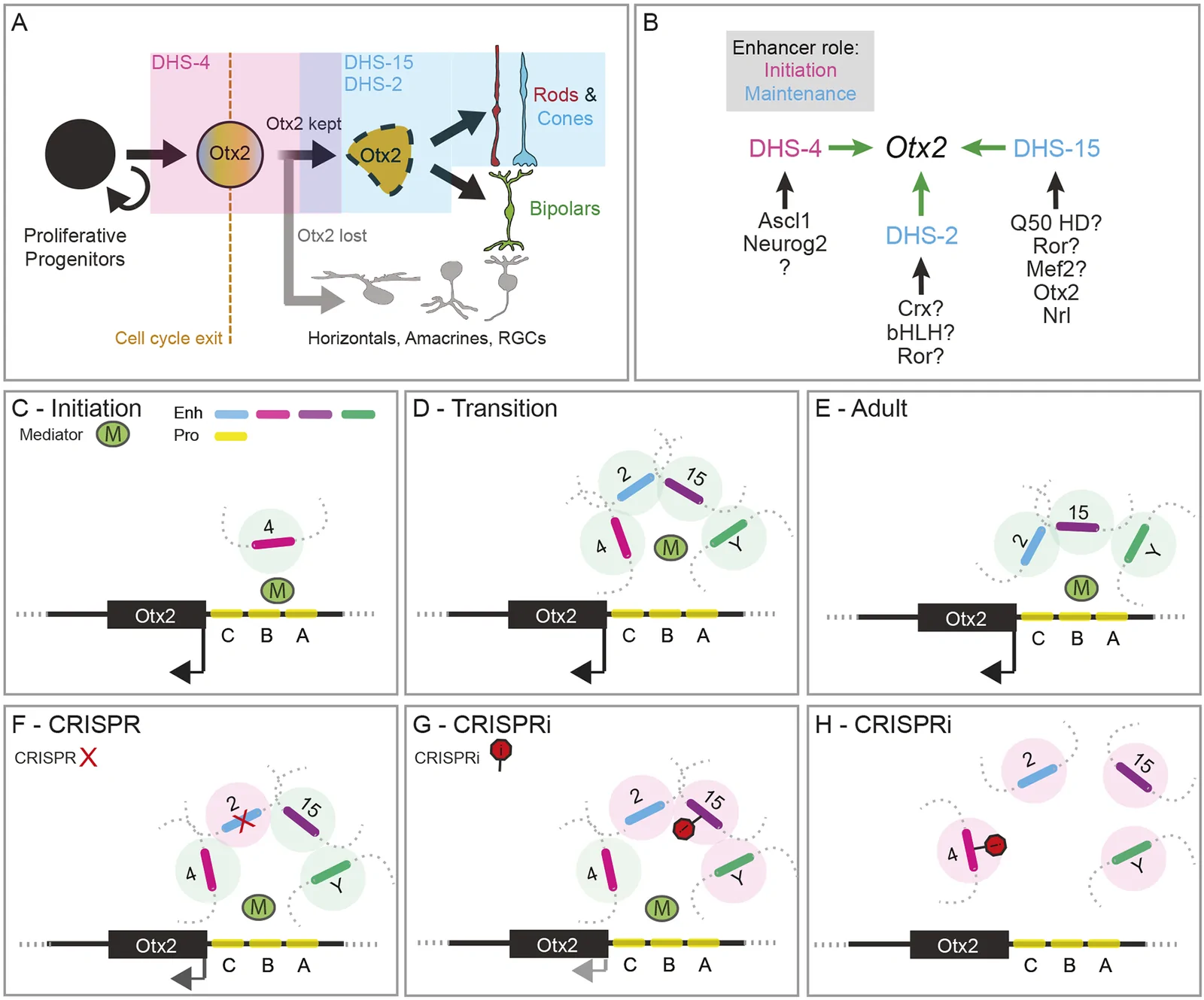
**Proposed *Otx2* enhancer expression, regulation and function in the retina.** (A) Scheme showing cell fate specification, *Otx2* expression, and enhancer activity. DHS4 initiates *Otx2* expression and has a broader lineage than DHS2 and DHS15, which only give rise to photoreceptors and bipolar cells. DHS2 and DHS15 remain active in rods and cones, but not bipolar cells. RGCs, retinal ganglion cells. (B) Potential upstream regulation of DHS2/4/15 is different for each enhancer (see also Kaufman et al., 2021). bHLH, basic helix-loop-helix; HD, homeodomain. (C-E) Proposed interactions between *Otx2* enhancers and promoters across development. (C) DHS4 interacts with *Otx2* promoters to initiate gene expression. (D) As OTX2^+^ cells select their fates, DHS2, DHS15 and other enhancers (Y) form a hub that interacts with promoters to maintain expression. (E) In mature OTX2^+^ cells, DHS4 is no longer active. DHS2, DHS15 and other enhancers (Y) sustain expression in photoreceptors while others (Y) are needed for bipolar cell expression. Enh, enhancer; Pro, promoter. (F-H) Potential mechanisms of enhancer behavior after perturbation. (F) CRISPR targeting (X) modestly reduces OTX2 expression, which may be due to mutations or steric hinderance disrupting the enhancer. This simple loss of function likely enables other enhancers to substitute and restore expression. (G) CRISPRi (stop sign) against DHS2 or DHS15 had a stronger cell fate phenotype than simultaneously targeting these enhancers with conventional CRISPR, suggesting that interacting enhancer function is blocked. The incomplete phenotype may indicate that CRISPRi disrupts only one of multiple enhancer hubs. (H) The strong DHS4 CRISPRi phenotype may be due to the disassembly of enhancer hubs, disrupting 3D genomic architecture, and preventing interactions with the *Otx2* promoters.

Fine spatiotemporal control of *Otx2* gene expression is essential for normal retinal development. We characterized DHS2 and DHS15 activity along with the function of several *Otx2* enhancers and promoters. Unlike DHS4, expression of which is early and transient (Wilken et al., 2015; Kaufman et al., 2021), DHS2 and DHS15 drove highly similar patterns of sustained activity specifically within developing OTX2^+^ cells and mature photoreceptors. This suggests that DHS4 initiates *Otx2* expression, which is then sustained in rods, cones and developing bipolar cells by the activity of DHS2, DHS15 and additional enhancers (Fig. 7A). We employed a matched CRISPR and CRISPRi approach to discover how *cis*-regulatory elements control *Otx2* expression and fate choice during development. Our data argue that all three *Otx2* enhancers are essential and that flexibility in how these elements interact and substitute for each other controls cell fate specification. Like other complex genes with multiple developmental roles, *Otx2* may require a high degree of *cis*-regulatory flexibility to ensure gene expression is robust and regulated correctly across numerous contexts (Kvon et al., 2021; Lin et al., 2022; Kim and Wysocka, 2023).

### DHS2 and DHS15 use distinct mechanisms to drive similar expression patterns

DHS2 and DHS15 specifically marked OTX2^+^ cells, had highly similar activity patterns, and were frequently (68-80%) active in the same cells. Both broadly marked developing OTX2^+^ cells, but their activity persisted only in rods and cones. This suggests that DHS2 and DHS15 activity starts after cell cycle exit, beyond the point when cells can adopt OTX2-negative fates (Fig. 7A). This contrasts with DHS4, expression of which is earlier, transient and includes OTX2-negative cell types in its lineage (Fig. 7A) (Kaufman et al., 2021). These observations suggest the following model (Fig. 7A-E). Early in development, DHS4 becomes active and interacts with the *Otx2* promoters to initiate expression (Fig. 7C). Next, DHS2, DHS15, and possibly other unknown enhancers, are engaged to maintain *Otx2* expression as cell fates are specified (Fig. 7D). In the mature retina, DHS2 and DHS15 are used to maintain *Otx2* expression in photoreceptors while additional enhancer(s) account for bipolar cell expression (Fig. 7E). It is unclear whether DHS4 and DHS2/15 have overlapping activity due to the long half-life of fluorescent reporters (Figs S2, S3, Fig. 7A). However, we observed that DHS4-driven transcripts were present in postmitotic *Otx2*^+^ cells, consistent with DHS4 activity briefly overlapping with that of DHS2/15 (Kaufman et al., 2021). Dissection of enhancer activity using single-cell RNA-sequencing and pseudotime analysis will define the temporal and lineage relationships between these enhancers. This will empower the use of these enhancers as precision tools for manipulating gene expression during retinal development.

The essential regions of DHS2 and DHS15 were highly conserved, but were not homologous to each other. Binding site predictions for each essential region revealed shared and unique features. Both DHS2 and DHS15 had predictions for K50 homeodomain and ROR transcription factor binding. RORβ is made by developing and mature photoreceptors (Jia et al., 2009; Liu et al., 2013; Fu et al., 2014; Srinivas et al., 2006) and may explain why these enhancers remain active in rods and cones. K50 homeodomain transcription factors include *Otx2* and its downstream target *Crx*, which are expressed by photoreceptors and bipolar cells (Yamamoto et al., 2020; Burglin and Affolter, 2016; Furukawa et al., 1997b; Chen et al., 1997; Nishida et al., 2003). Chan and colleagues identified an enhancer sequence (O5) that is nearly identical to DHS2 bit 10 and demonstrated that it contains an essential K50 homeodomain binding site (Chan et al., 2020). The K50-binding sites suggest that OTX2 binds its own enhancers in a positive-feedback fashion. To explore this, we examined OTX2 cleavage under targets and tagmentation (CUT&Tag) and chromatin immunoprecipitation (ChIP) datasets from E14.5 and adult murine retinas (Fig. S16) (Ge et al., 2023; Samuel et al., 2014). These datasets showed negligible OTX2 binding at DHS2, a modest peak at DHS4 (but in the non-essential C region) (Kaufman et al., 2021), and strong binding at DHS15 (Fig. S16). This suggests that OTX2 binds the DHS15 enhancer to maintain its own expression while its downstream target CRX regulates the DHS2 enhancer (Fig. 7B). Unexpectedly, CRX ChIP data did not show a strong signal at DHS2 in adult retina (Corbo et al., 2010). The DHS15 enhancer had binding predictions for Q50 homeodomain and MEF2 factors (Fig. 7B). Several MEF2 factors are made by photoreceptors (Nagar et al., 2017; Omori et al., 2015; Andzelm et al., 2015; Ochoa Olmos et al., 2025), as is the Q50 homeodomain factor RAX (Furukawa et al., 1997a; Rohde et al., 2011; Mathers et al., 1997; Muranishi et al., 2011; Ochi et al., 2004). DHS2 contained bHLH (e.g. NEUROD1) and MAF (e.g. NRL) binding predictions in its essential sequence region (Fig. 7B). However, examining NRL CUT&Tag data (Liang et al., 2022) revealed binding to the DHS15 enhancer, which increased over development (Fig. S17). It is unclear whether NRL is required for expression early in development and how DHS15 is made in cones, which lack NRL. Although we have yet to determine which factors bind to these enhancers across development, our predictions argue that the expression patterns of DHS2 and DHS15 are controlled by shared and distinct upstream regulators (Fig. 7B). This may enable these enhancers to work in parallel to ensure robust *Otx2* expression during development.

### DHS2 and DHS15 are necessary for *Otx2* expression and fate specification

Perturbing DHS2 or DHS15 acutely reduced the number of cells that expressed OTX2. Conventional CRISPR with guides targeting the O5 enhancer (i.e. DHS2) also reduced *Otx2* expression (Chan et al., 2020). Our prior work showed that DHS4 perturbation caused an acute loss of OTX2 as well (Kaufman et al., 2021). Together, these outcomes argue that DHS2, DHS4 and DHS15 are each necessary for *Otx2* expression during development.

Despite their initial importance, targeting DHS2/4/15 with conventional CRISPR resulted in only minor cell fate changes after the completion of neurogenesis (Kaufman et al., 2021). This is tied to the ability of targeted cells to express OTX2, as CRISPR mutagenesis of the *Otx2* coding region resulted in severe OTX2 loss and major cell fate changes (Ghinia Tegla et al., 2020; Kaufman et al., 2021). CRISPR mutation screening revealed that DHS2 and DHS15 had few permanently disabling mutations. However, we estimate that at least one-third of mutations could not be detected for technical reasons. The mechanisms and kinetics of CRISPR mutagenesis vary between proliferative and postmitotic cells, such as retinal neurons (Ramadoss et al., 2025). Postmitotic cells use conventional non-homologous end joining pathways to repair double-strand breaks leading to small indels and substitutions, whereas proliferative cells can use alternative end joining pathways that generate larger deletions (Brinkman et al., 2018; Ramadoss et al., 2025) that could span guide pairs. Neuronal cells also take longer to accumulate mutations (Ramadoss et al., 2025), which may explain why some photoreceptors in *Otx2* CRISPR conditions lacked OTX2 expression. This could reflect a subpopulation of late mutagenizing cells that disable *Otx2* after photoreceptor identity is established, when OTX2 is no longer needed (Beby et al., 2010; Housset et al., 2013; Kaufman et al., 2021). Our mutation screening suggests that a blend of proliferative and postmitotic CRISPR mutagenesis features occur in the retina. Nonetheless, the strong OTX2 reduction seen with dCas9 enhancer targeting argues that the acute CRISPR phenotypes were largely due to the physical effects of Cas9, which can block transcription factor binding to *cis*-regulatory elements (Auradkar et al., 2023). Cas9 binding is persistent and holds onto cleaved DNA for protracted time periods, potentially leading to steric hinderance of enhancer activity (Richardson et al., 2016; Sternberg et al., 2014). Although the number of mutations seen in DHS2 and DHS15 was low 3 days after electroporation, it is possible that mutations continue to slowly accumulate in postmitotic neurons (Ramadoss et al., 2025), eventually preventing Cas9 binding and steric hinderance. Nonetheless, the relatively quick recovery of OTX2 expression and cell fate choice we observed with conventional CRISPR is likely mediated by enhancer substitution.

To explore substitution, we used CRISPRi to disrupt enhancers and their interacting partners (Lin et al., 2022; Hamamoto and Fukaya, 2022; Lim and Levine, 2021; Morris et al., 2023; Fulco et al., 2016). The strong CRISPRi phenotypes argue that this perturbation disrupts both the targeted enhancer and interacting *cis*-regulatory elements that substitute for their loss. The moderate cell fate changes seen with simultaneous DHS2/15 CRISPR targeting at P21 imply that these enhancers are partially redundant. However, stronger fate changes from enhancer CRISPRi argue that additional *cis*-regulatory elements interact with DHS2 and DHS15 to sustain *Otx2* expression during development. The strong DHS4 CRISPRi phenotype could reflect an indispensable requirement for *Otx2* initiation or that DHS4 enhancer interactions with other *cis*-regulatory elements are essential for *Otx2* expression. In support of the latter, we found that mutating the upstream regulators of DHS4, *Ascl1* and *Neurog2*, was unable to permanently block *Otx2* expression (Kaufman et al., 2021). Nonetheless, it is possible that DHS4 is both essential on its own and key for fostering *cis*-regulatory interactions. Future experiments that test how *Otx2* enhancer CRISPRi perturbations impact chromatin status and 3D genomic architecture will reveal how enhancers work together to regulate gene expression.

### *Otx2* promoter and enhancer flexibility during retinal development

Disruption of *Otx2 cis*-regulatory elements suggested that they are used flexibly during development. None of our promoter perturbations caused large changes in OTX2 expression. CRISPRi against the 1A region moderately decreased the number of OTX2^+^ cells embryonically. However, a small increase in the number of OTX2^+^ cells was seen when this region was targeted postnatally. This seemingly paradoxical result may be due to enhancer release and re-targeting, which can occur when disabling a promoter causes enhancers to change their promoter preference (Oh et al., 2021). While each *Otx2* promoter is active during development (Fossat et al., 2005), their transcripts have different 5′ untranslated sequences. Stability of these transcripts may differ, altering OTX2 expression dynamics and fate choice. Future work is needed to determine the necessity of each *Otx2* promoter, whether DHS2/4/15 have preferred promoters, if enhancer disruption alters promoter usage, and whether promoter choice impacts *Otx2* expression kinetics or enhancer substitution.

The use of matched CRISPR and CRISPRi perturbations suggested that *Otx2* enhancers can substitute for each other, providing regulatory flexibility during development. There are several non-exclusive mechanisms that can explain how *Otx2* enhancers substitute for each other. The simplest mechanism is that *Otx2* enhancers behave in an incompletely redundant fashion (i.e. like shadow enhancers) (Kvon et al., 2021). It has been proposed that seemingly redundant enhancers function by increasing expression robustness, which buffers transcriptional noise, environmental stresses, and possibly even deleterious mutations to canalize development (Kvon et al., 2021; Kim and Wysocka, 2023; Lin et al., 2022). While DHS2 and DHS15 have highly similar expression patterns, our experiments suggest that additional substituting enhancers exist. A likely candidate is the O7 enhancer described by Chan and colleagues (Chan et al., 2020). This enhancer is located between DHS2 and DHS4, shows sustained activity in photoreceptors, and contains an essential K50 homeodomain-binding site (Chan et al., 2020).

Cryptic enhancers are another source of regulatory flexibility that may be used to overcome mutations. This proposed class of enhancers is thought to be inactive until cells experience stress or other perturbed conditions (Kvon et al., 2021; Durai et al., 2019; Kalay et al., 2019; Narasimhan et al., 2015). While conventional CRISPR can alter sequences to block enhancer activity, these mutations may also create new spatial relationships or binding sites that result in novel (‘cryptic’) enhancer activity that partially rescues gene expression. Cryptic activity could arise when disabling enhancers cause their upstream regulators to accumulate and bind to sequences that typically have little or no activity, thereby ‘awakening’ them. This could also alter the spatial and temporal specificity of intact enhancers. For example, perturbing DHS2 may allow bHLH factors to accumulate and bind to the DHS4 enhancer (Kaufman et al., 2021), aberrantly sustaining its normally brief activity and rescuing *Otx2* expression and fate choice.

Enhancer interactions and substitution flexibility could be due to changes in the 3D genomic architecture at the *Otx2* locus during development. Active enhancers are thought to be brought into 3D complexes (hubs) where they closely interact with each other and promoters to drive transcription (Thomas et al., 2021; Kim and Wysocka, 2023; Lim and Levine, 2021; Hamamoto and Fukaya, 2022). The mild CRISPR cell fate phenotypes may indicate that disabling mutations or steric hinderance from Cas9 does not alter 3D genomic architecture or disrupt enhancer hub function (Fig. 7F). However, the acute CRISPR effect on OTX2 expression may be due to steric hinderance temporarily impacting the creation, maintenance or function of enhancer hubs (Sapozhnikov and Szyf, 2021; Gao et al., 2014; Howe et al., 2017). The stronger CRISPRi cell fate phenotypes could be the result of epigenetic modifications from the dCas9-KRAB/MECP2 fusion protein silencing all enhancers that are close together in a 3D hub environment (Fig. 7G). Enhancer CRISPRi may also alter genome architectural dynamics, disrupting hub formation, maintenance, and interactions with promoters (Fig. 7H). Much remains to be discovered about the mechanisms that control *Otx2 cis*-regulatory flexibility and how this robustness impacts retinal cell fate specification.

### Limitations of our study

There were two major limitations in our exploration of *Otx2* enhancer function. First, we measured OTX2 expression at the protein level and categorized cells as either positive or negative. While this binary classification enabled us to quantify cell fate changes, it was not able to account for the likely possibility that these enhancers impact *Otx2* transcription levels (Chan et al., 2020). For example, CRISPR may lower *Otx2* transcript levels at all time points, although not enough to alter the formation of photoreceptors and bipolar cells. Second, we had difficulty measuring how extensively our perturbations disrupted enhancer function at the cell level. Mutational variance and the paucity of guide-spanning deletions made comparing phenotypic magnitude between targeted loci challenging. It was also difficult to determine whether differences in cell fate phenotypes between CRISPRi conditions were due to how enhancer interactions were perturbed or instead by how efficient CRISPRi was at silencing each enhancer. Fully understanding how *Otx2* enhancers function and interact with each other will require experiments that address transcriptional levels, epigenetic effects from CRISPRi, and the use of transgenic mice with uniform surgical *Otx2* enhancer deletions.

## MATERIALS AND METHODS

### Mice, electroporation and tissue collection

All animal procedures were approved by the University of Colorado Denver Institutional Animal Care and Use Committee. Male and female mice were used in equal numbers. Retinas were harvested at either E13.5, E14.5 or P0 from wild-type CD1 mice (strain 022, Charles River Laboratories) or *ROSA-RFP* mice [*B6.Cg-Gt(ROSA)26Sor^tm14(CAG-tdTomato)Hze^/J*] (strain 007914, The Jackson Laboratory) (Madisen et al., 2010).

To assess enhancer activity and function, retinas from E13.5, E14.5 or P0 mice were dissected, electroporated and cultured as *ex vivo* explants, as previously described (Mills et al., 2017; Goodson et al., 2020b; Kaufman et al., 2021; Bachu et al., 2022; Goodson et al., 2020a). Briefly, eyes were dissected in cold Hank's Balanced Salt Solution (HBSS, with Ca^2+^ and Mg^2+^) (Corning) containing 6 mg/ml glucose and 0.05 M HEPES. For P0 retinas, the tissue was dissected freely, while embryonic eyes were dissected to retain the retina and lens. A 1.5 µl volume of electroporation solution (45 µg plasmid DNA in 9 µl water, with 6 µl Methyl Green in glycerol) was applied to the photoreceptor side of the retina. Electroporations were carried out using five square-wave pulses (50 V for 50 ms, with 250 ms intervals) using a Bio-Rad Gene Pulser Xcell (Mills et al., 2017; Goodson et al., 2020b; Kaufman et al., 2021; Bachu et al., 2022). However, most *ex vivo* electroporations were conducted using an 8×5×3 mm (Fukuda Type) chamber (CUY520P5, Bulldog Bio). The same electroporator settings described above were used with the chamber. A mixture of 1 µg/µl plasmid DNA in 100 µl PBS was made for each electroporation and added to the chamber. Retinal explants were then delicately deposited by gravity into the chamber using a plastic transfer pipette, to avoid diluting the mixture. After electroporation, explants were cultured in growth media (Neurobasal Medium, 1× N2 supplement, 1× L-glutamine, 1× penicillin/streptomycin and 1% fetal bovine serum; Gibco/Thermo Fisher Scientific) in a 12-well plate. Cultures were maintained at 37°C under 5% CO_2_ with gentle nutation (8 RPM) for up to 72 h to facilitate oxygenation. Long-term explants (≥7 days) were electroporated as above, the lens removed, and the retina flattened ganglion cell-side down onto Omnipore membrane filters (0.45 μm) (Millipore Sigma). These were then transferred onto 30 mm diameter 0.4 μm Millicell CM cell culture inserts (Millipore Sigma) in a 6-well plate. The flattened explants were cultured at the air-media interface in growth media containing 10% fetal bovine serum. Flattened explants were grown with 5% CO_2_ in a 37°C incubator, but without nutation. Half of the media was changed every other day (Goodson et al., 2020a,b; Kaufman et al., 2021; Bachu et al., 2022; Mills et al., 2017).

For *in vivo* electroporations (de Melo and Blackshaw, 2011; Goodson et al., 2020a; Kaufman et al., 2021), newborn CD1 or *ROSA-RFP* mice were cryo-anesthetized on ice for 3-5 min. Once unresponsive to tail pinch, pups were transferred to an ice pack for surgery. A 31 G needle was used to create a lengthwise incision along the eyelid to expose the eye. A second 31 G needle was inserted along the nasal sclera, adjacent to the cornea, to create a small hole. A 32 G blunt-tip Hamilton syringe was used to inject 0.5 µl of a 2 µg/µl DNA solution (in sterile H_2_O) into the subretinal space of the central retina. Electroporation was performed using five square-wave pulses (80 V for 50 ms duration, with 950 ms intervals) using a Bio-Rad Gene Pulser Xcell and Tweezertrodes (de Melo and Blackshaw, 2011; Goodson et al., 2020a; Kaufman et al., 2021). Following the procedure, Neosporin was applied to the eye and Buprenex (0.1 mg/kg) was administered subcutaneously for pain relief. The pups were placed on a circulating water heating pad at 37°C until pink and responsive and then returned to their home cage.

After culturing, explants were fixed in 2% paraformaldehyde for 20 min at room temperature. Eyes subjected to *in vivo* electroporations were punctured with a 28 G needle in the cornea and then fixed in 2% paraformaldehyde for 1-2 h at room temperature. Fixed tissue was cryopreserved in progressive sucrose gradients (10%, 20%, 30%), embedded in O.C.T. medium (Sakura), and stored at −80°C until sectioning.

### Immunohistochemistry

Retinal tissues were cryosectioned at 10 µm using a Thermo Fisher Scientific MICROM HM 550 cryostat and mounted on Colorfrost Plus microscope slides (Thermo Fisher Scientific). Immunohistochemistry was performed as previously described (Brzezinski et al., 2010; Bachu et al., 2022; Goodson et al., 2018, 2020a,b; Kaufman et al., 2021; Brzezinski et al., 2013). Briefly, slides were blocked at room temperature for 1 h in a milk block solution (supernatant from 5% dry milk and 0.5% Triton X-100 dissolved in PBS). Primary antibodies (Table S2) were diluted in milk block solution, and the slides were incubated overnight (12-18 h) at room temperature. After washing with PBS, the slides were incubated for 1 h at room temperature with fluorescently labeled secondary antibodies (Table S2) diluted in milk block. The slides were washed in PBS again and then mounted for imaging.

### Imaging, quantification and statistics

Images of retinal sections were acquired using Nikon C2 or Nikon AXR laser-scanning confocal microscopes. Images were captured in ND2 file format as a 1024×1024 pixel area using sequential laser passes to capture each fluorescent color channel. Three to five *z*-stacks (1-1.5 μm per slice) were captured, maximum-intensity *z*-projected with ImageJ (Goodson et al., 2020a; Kaufman et al., 2021), and minimally processed using Adobe Photoshop or GNU Image Manipulation Program (GIMP) to equalize levels. Quantification of immunostained nuclei was carried out manually from 200× or 400× magnification fields. In most manipulations, the percentage of electroporated cells that co-express a given nuclear marker was determined. Quantifications were made on a minimum of three explants or eyes per condition. Data were tabulated in Microsoft Excel and statistically evaluated in GraphPad Prism. Statistical significance in Fig. 1 was calculated using the Mann–Whitney non-parametric test. For all other figures, the non-parametric Kruskal–Wallis test with Dunn's multiple comparisons was used to evaluate significance. *P*<0.05 was considered statistically significant. Graphs were generated using GraphPad Prism using SuperPlot (Lord et al., 2020) features to distinguish biological replicates.

### Enhancer cloning and mutagenesis

For insertional and mutagenesis cloning, we used In-Fusion (Takara Bio) PCR-based cloning according to the manufacturer's protocols (Kaufman et al., 2021; Bachu et al., 2022; Goodson et al., 2020b; Mills et al., 2017). Primers were designed to target the mouse genome (GRCm38/mm10 assembly), and sequences were amplified from C57BL/6J mouse genomic DNA (strain #000664, The Jackson Laboratory). Enhancer sequences were inserted into the pMin-nGFP plasmid, upstream of a minimal TATA promoter to drive nuclear-localized (n) GFP expression (Wilken et al., 2015; Goodson et al., 2020b; Kaufman et al., 2021; Mills et al., 2017). A similar process was used to clone into a pMin-nCherry backbone. Tiled mutagenesis of DHS2 and DHS15 enhancers was performed by inverse PCR using primers to convert target sequences to 10-12 bp blocks of adenine nucleotides (Mills et al., 2017; Kaufman et al., 2021; Bachu et al., 2022). For lineage-tracing constructs, the pMin-nGFP vector (Wilken et al., 2015; Kaufman et al., 2021; Bachu et al., 2022; Goodson et al., 2020b) was modified to co-express Cre recombinase. All enhancer sequences are listed in Table S2.

### Design of CRISPR/Cas9 plasmids

The pSpCas9(BB)-2A-GFP (PX458) plasmid was obtained from Addgene (Addgene plasmid #48138, deposited by Dr Feng Zhang, Massachusetts Institute of Technology, Cambridge, USA) (Ran et al., 2013). This plasmid was modified as previously described to replace the *Cbh* promoter with a human EF1α promoter (Goodson et al., 2020a; Kaufman et al., 2021). A subsequent modification was made to replace GFP with mCherry to establish a second reporter color (Goodson et al., 2020a; Kaufman et al., 2021). Subsequently, histone H2B nuclear localization fusions were added to both reporter variants to create strong stable reporters. These modifications resulted in two all-in-one reporter versions: cytoplasmic (EF1α-pSpCas9-2A-GFP and EF1α-pSpCas9-2A-mCherry) and nuclear-localized (EF1α-pSpCas9-2A-H2B-GFP and EF1α-pSpCas9-2A-H2B-mCherry). Further modifications were made to generate CRISPRi backbones. A plasmid generated by the Church laboratory (Yeo et al., 2018) containing KRAB and MeCP2 domains fused with a dCas9 protein was purchased from Addgene (Addgene plasmid #110821). We adapted this plasmid into our all-in-one system by incorporating a T2A-H2B-GFP (or mCherry) sequences downstream of the MeCP2 domain. These plasmid backbones have been deposited in Addgene (https://www.addgene.org/Joseph_Brzezinski/).

Guide RNAs for inducing loss-of-function phenotypes were designed to target protein-coding exon sequences for direct gene disruption or to flank essential enhancer regions (Goodson et al., 2020a; Kaufman et al., 2021). Guides were designed using sequences and coordinates from the mm9 genome assembly. The guides were designed using the CRISPR Design and Analyze Guides tool on Benchling.com, with priority given to guides with high on-target scores (greater than 70) and low off-target potential (less than 50). The selected guides were then checked for relevant off-target effects using BLAST. Guide sequences that did not target coding sequences in other genes were selected. The best two guides (Table S2) were ordered as paired oligos from IDT. CRISPR/CRISPRi plasmids were digested with BbsI and used for Golden Gate cloning to insert the annealed, phosphorylated guide oligos (Ran et al., 2013). To generate multi-guide plasmids, a combination of PCR and In-Fusion cloning was employed. Briefly, primer pairs spanning the backbone and 5′ end of the U6 cassette along with another primer targeting the 3′ end were generated. PCR was conducted on a CRISPR/CRISPRi plasmid (5 ng) that had previously incorporated a guide RNA sequence using Golden Gate cloning. This amplified the entire U6 guide RNA cassette including a KpnI restriction site present between the U6 cassette and the Cas9 sequence. The correct base-pair length of the PCR products was confirmed by gel electrophoresis. Concurrently, another CRISPR/CRISPRi plasmid (2 µg) containing a previously incorporated guide RNA sequence was digested using KpnI for at least 30 min at 37°C. After linearizing the ‘acceptor’ plasmid using KpnI, the PCR product containing the ‘donor’ guide was incorporated into the ‘acceptor’ plasmid via In-Fusion cloning as previously described (Kaufman et al., 2021). This protocol was used to generate all dual-guide plasmids used in this manuscript. To generate the four-guide plasmid, the same protocol was conducted using the DHS2-dual guide and DHS15-dual guide plasmids. In this case, the DHS2-dual guide was the ‘acceptor’ plasmid and the DHS15-dual guide was the ‘donor’ plasmid. Finally, we generated dCas9-only plasmids using our CRISPRi plasmids as a template. Inverse PCR was employed to remove the KRAB and MeCP2 domains while retaining all the other sequences within the CRISPRi plasmids for DHS2 and DHS15. The resulting plasmids contained the same nuclease-deficient Cas9 (Cas9_N−_) generated previously (Mali et al., 2013). However, this also omitted one of the two nuclear localization sequences in the construct. In all cases, cloning was confirmed by Sanger or Nanopore sequencing.

### CRISPR mutation screening

To measure the spectrum of mutations that occur in retina cells electroporated with conventional CRISPR plasmids, we took a deep-sequencing approach. P0 CD1 retinal explants were electroporated with CRISPR constructs, incubated for 3 days, and fluorescent cells isolated by fluorescence-activated cell sorting as previously described (Kaufman et al., 2019). To reduce the presence of contaminating DNA from lysed wild-type cells, sorted cells were pelleted (5 min at 300 ***g***) and incubated with 2 U of DNase (NEB, M0303S, B0303S) at 37°C for at least 30 min. Cells were washed three times with PBS before proceeding to genomic DNA extraction (QIAGEN, 69504). PCR primers were designed and used with CloneAmp HiFi PCR premix (639298) to make small amplicons from sorted cell genomic DNA that flanked guide sites (Table S3). Amplicons approximating the expected size and smaller were isolated using agarose gel electrophoresis (Takara Bio, 740609.50). These amplicons were sent for next-generation sequencing (NGS) (Quintara Biosciences) with a target depth of >10,000 paired- or single-end reads per sample. We were unable to generate successful amplicons for DHS15 guide 1, and guide 1 for promoter 1A.

FASTQ files generated from NGS were converted to BAM files. The mouse reference genome (mm39/GRCm39) from the UCSC Genome Browser was indexed using BWA (version 0.7.17) for read alignment. Paired-end FASTQ files from Quintara Biosciences NGS sequencing runs were aligned to the mm39 reference genome using BWA-MEM with default parameters. Aligned reads were sorted and converted to BAM format using SAMtools (version 1.x), which were used for visualization and the CrispRVariants pipeline (Lindsay et al., 2016). This pipeline counts, localizes and plots both indel and single-nucleotide variants relative to a guide RNA sequence, allowing for detailed analyses of CRISPR-Cas9 experiments. AI (Integrated AI Suite, from Cursor) was used to streamline code utilization in the CrispRVariants analysis. Outcomes are listed in Table S3 and the analysis pipeline is available in the GitHub repository: (https://github.com/MLKaufman/purvis-dev2025-crispr-outcomes/tree/main?tab=readme-ov-file).

### JASPAR transcription factor-binding predictions

Using the JASPAR 2024 track (Rauluseviciute et al., 2024) within the UCSC Genome Browser mm10 assembly, we used a moderately strict threshold of 250 to identify predicted transcription factor-binding sites in DHS2 and DHS15 critical regions. All transcription factors annotated within the JASPAR database were included in the prediction analysis. The full list of predicted transcription factor-binding sites for DHS2 and DHS15 is in Table S1.

### Visualization of external datasets

Genome tracks were visualized using the UCSC Genome Browser. Mouse (Aldiri et al., 2017) and human (Marchal et al., 2022) whole-retina ATAC-seq datasets were used in Fig. S1. Mature mouse RPE ATAC-seq data (Liao et al., 2024) was used in Fig. S13. Mature and E14.5 mouse retinal OTX2 ChIP-seq (Samuel et al., 2014) and CUT&Tag (Ge et al., 2023) datasets, respectively, were used in Fig. S16. NRL CUT&Tag datasets from P2, P4, P10 and P28 mouse retinas (Liang et al., 2022) were used in Fig. S17.

## Supplementary Material



10.1242/develop.204881_sup1 Supplementary information

Table S1. List of JASPAR predicted binding sites for DHS2 and DHS15 minimal enhancers.

Table S2. List of enhancer sequences, guides, and antibodies used.

Table S3. Mutation spectrum analysis in retinal cells electroporated with conventional CRISPR guides.
