# Supplementary material for: A robust cis-regulatory network ensures Otx2 expression during retinal development
Source: Development. 2026 Mar 27;153(6):dev204881. doi: 10.1242/dev.204881 (PMC13495790; doi:10.1242/dev.204881)
Supplement: Supplementary file 1 [file develop-153-204881-s1.pdf]

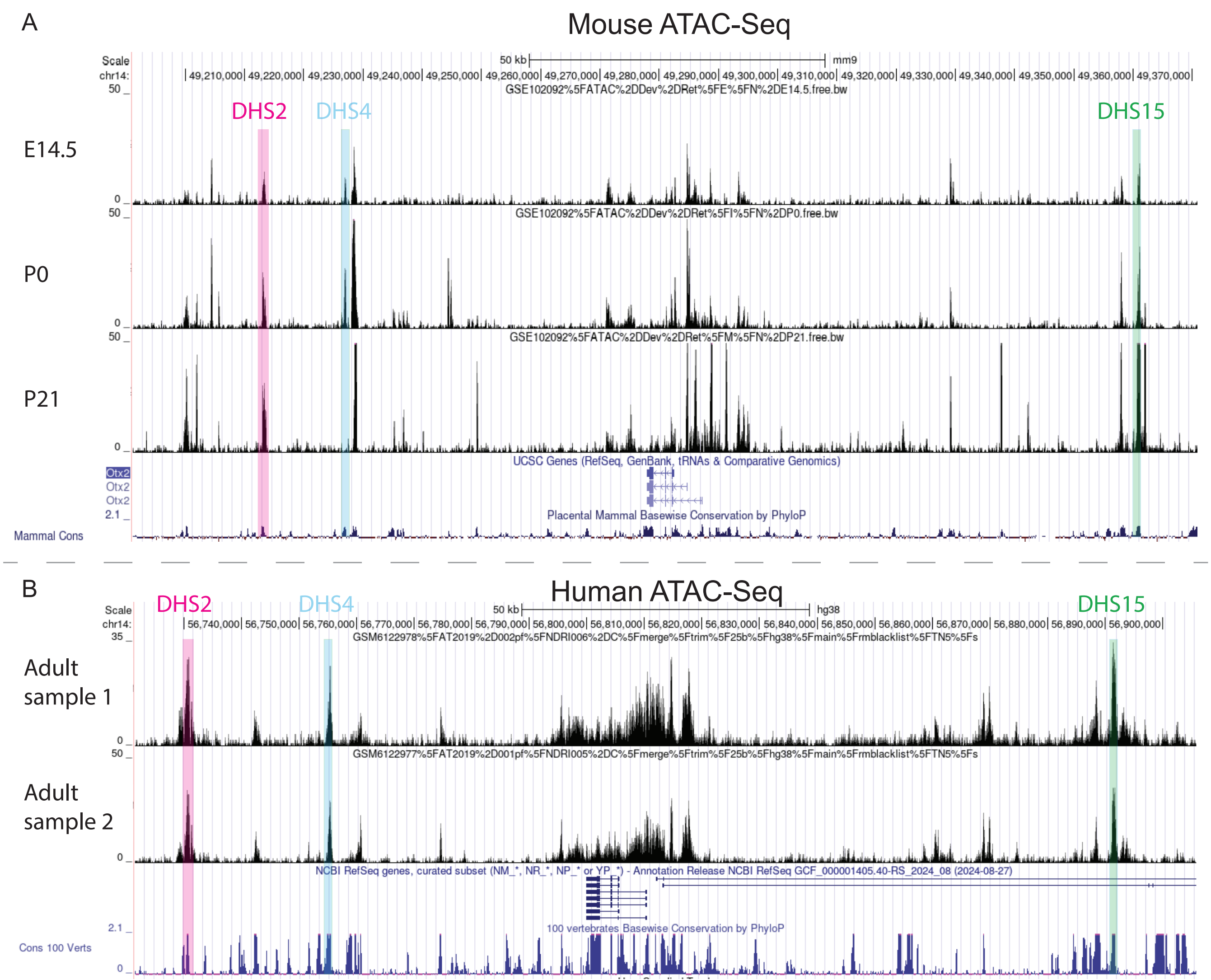

Fig. S1. Chromatin accessibility around *Otx2*. (A-B) Assay for transpose accessible chromatin (ATAC) sequencing (seq) tracks plotted on the USCS genome browser for mouse (mm10) (A) and human (hg38) (B). **(A)** ATAC-seq tracks from E14.5, P0, and P21 whole mouse retinas showing DHS2, DHS4 and DHS15 relative to the *Otx2* gene. **(B)** ATAC-seq tracks from two different adult whole retinal samples are visualized, highlighting DHS2, DHS4, and DHS15. All three enhancer regions are highly conserved.

Development • Supplementary information

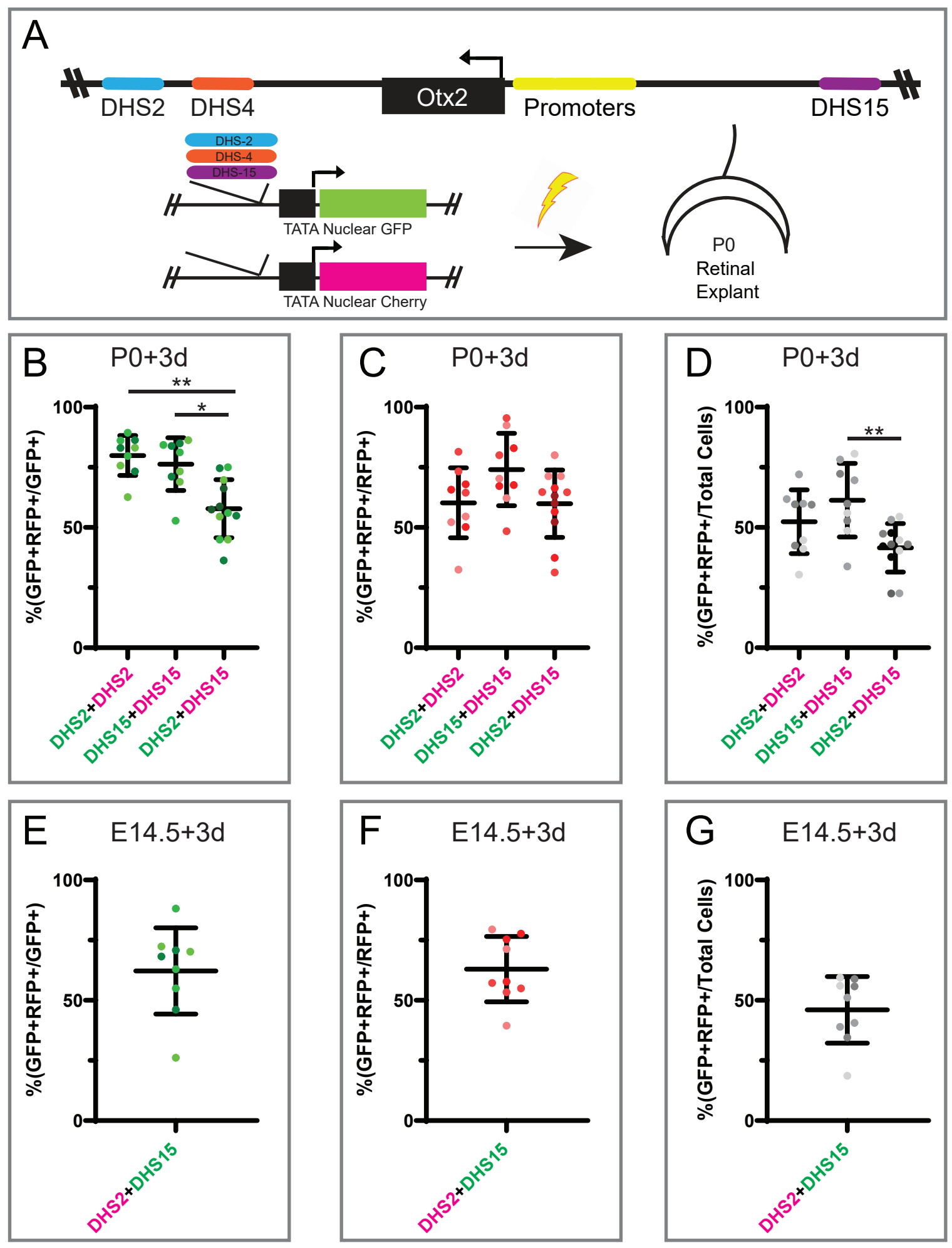

Fig. S2. Co-expression of DHS2 and DHS15 enhancers. (A) Enhancer cloning and electroporation scheme. Sequences were cloned upstream of a minimal TATA promoter to drive nuclear-localized (n) GFP or mCherry expression. E14.5 or P0 retinal explants were co-electroporated with DHS2-nGFP + DHS2-mCherry, DHS15-nGFP + DHS15-mCherry or DHS2-nGFP + DHS15-mCherry constructs using a 1:1 mixture and cultured for 3 days. **(B-D)** P0 or **(E-G)** E14.5 co-expression was quantified with the indicated denominators. For all plots, dots represent individual quantified images from a biological N ≥ 3 explants.

Development • Supplementary information

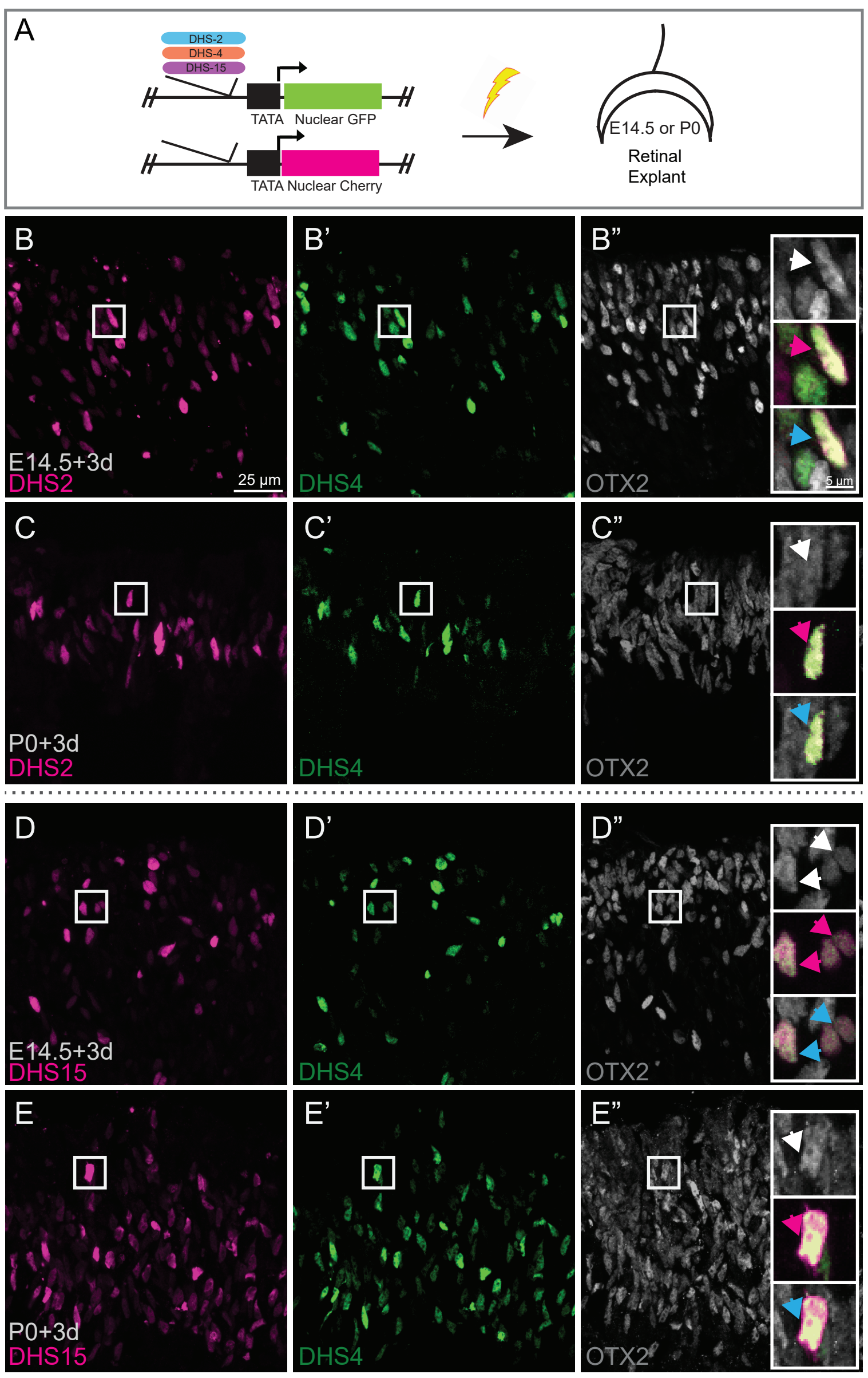

Fig. S3. Spatiotemporal characterization of *Otx2* enhancers. (A) Enhancer cloning and electroporation scheme. Sequences were cloned upstream of a minimal TATA promoter to drive nGFP or mCherry expression. E14.5 or P0 retinal explants were co-electroporated with a 1:1 mixture of plasmids and cultured for 3 days. **(B-E")** Sections immunostained for GFP, mCherry, and OTX2. Arrows mark single (white), double (magenta), and triple (cyan) labeled cells. Note overlap between DHS2 and DHS4 (B-C") and DHS15 and DHS4 (D-E") from both E14.5 and P0 electroporations. Scale bars are 25 µm for all panels and 5 µm for insets. The outer retina is up in all panels.

Development • Supplementary information

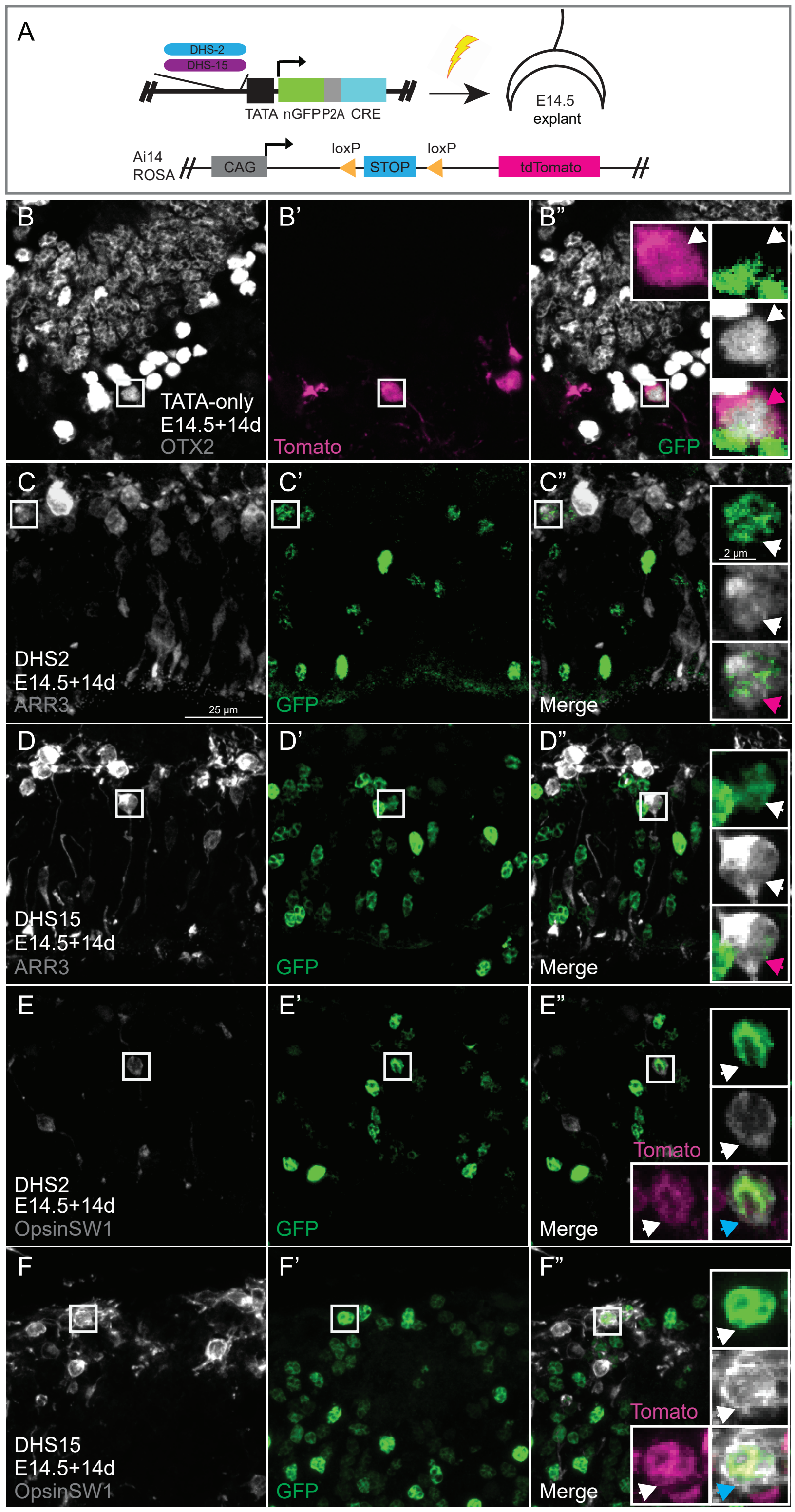

Fig. S4. Embryonic lineage tracing of DHS2 and DHS15 enhancers marks cones. (A) Lineage tracing scheme, as in Fig. 1. Enhancer sequences were cloned upstream of a minimal TATA promoter driving nGFP and Cre. As a Cre specificity control, a plasmid lacking an enhancer (TATA-only) was also used. Plasmids were electroporated at E14.5 into *ROSA-RFP* retinal explants to permanently label recombined cells with tdTomato. Explants were collected after 14 days of culture. **(B-B")** Images showing tdTomato+/GFP+/OTX2+ cells in TATA-only GFP-Cre electroporated retinal explants. Note the rare labeling and that lamination is often disrupted in these long-term cultures. **(C-C")** Images showing GFP+/ARR3 cells (cones) in DHS2-GFP-Cre and **(D-D")** DHS15-GFP-Cre electroporated explants. **(E-E")** Images showing GFP+/OPSW1+ cells (blue cones) in DHS2-GFP-Cre and **(F-F")** DHS15-GFP-Cre electroporated explants. Arrows mark single (white), double (magenta), and triple (cyan) labeled cells. Scale bars are 25 µm for all panels and 10 µm for insets. The outer retina is up in all panels.

Development • Supplementary information

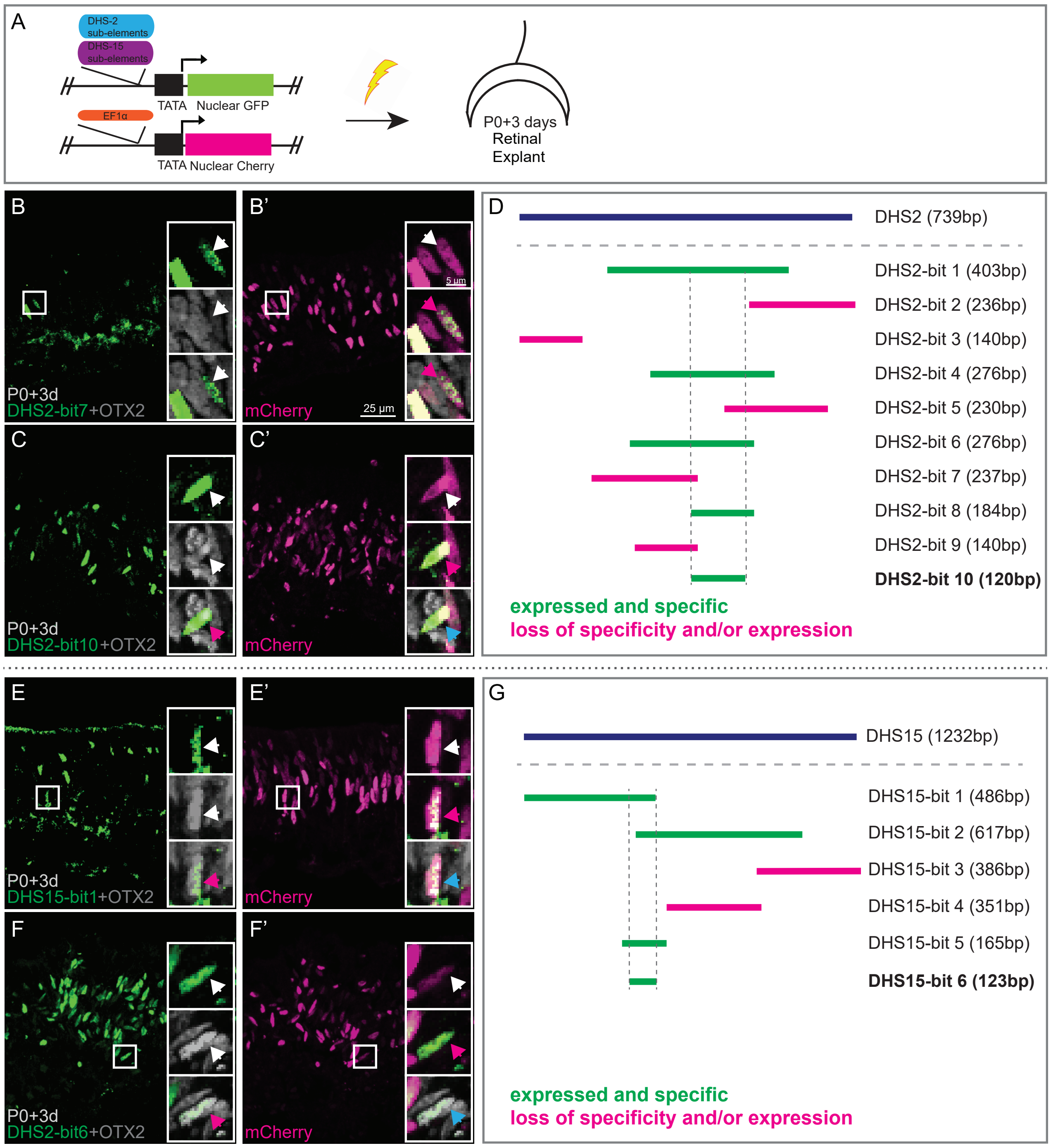

Fig. S5. Identification of essential DHS2 and DHS15 sequences. (A) Sub-elements of DHS2 and DHS15 were cloned into reporter vectors upstream of a minimal promoter (TATA) driving nGFP. These plasmids were co-electroporated with a positive control plasmid driving nCherry from a ubiquitous (EF1α) promoter. Plasmids were co-electroporated into P0 retinal explants, collected after 3 days of culture, and stained for GFP, mCherry, and OTX2. **(B-C', E-F')** Immunostained retinas from DHS2 (B-C') and DHS15 (E-F') enhancer dissection experiments. An example of intact activity and specificity and one of loss are shown for each enhancer. **(D, G)** Scheme showing relative sizes and locations of DHS2 (D) and DHS15 (G) sub-elements. Elements that retained expression and co-localization with OTX2 are colored green while elements that lost expression and/or OTX2 specificity are magenta. The minimal element that retained expression and co-localization with OTX2 is highlighted in bold and outlined with dashed lines. Arrows mark single (white), double (magenta), and triple (cyan) labeled cells. Scale bars are 25 µm for all panels and 5 µm for insets. The outer retina is up in all panels.

Development • Supplementary information

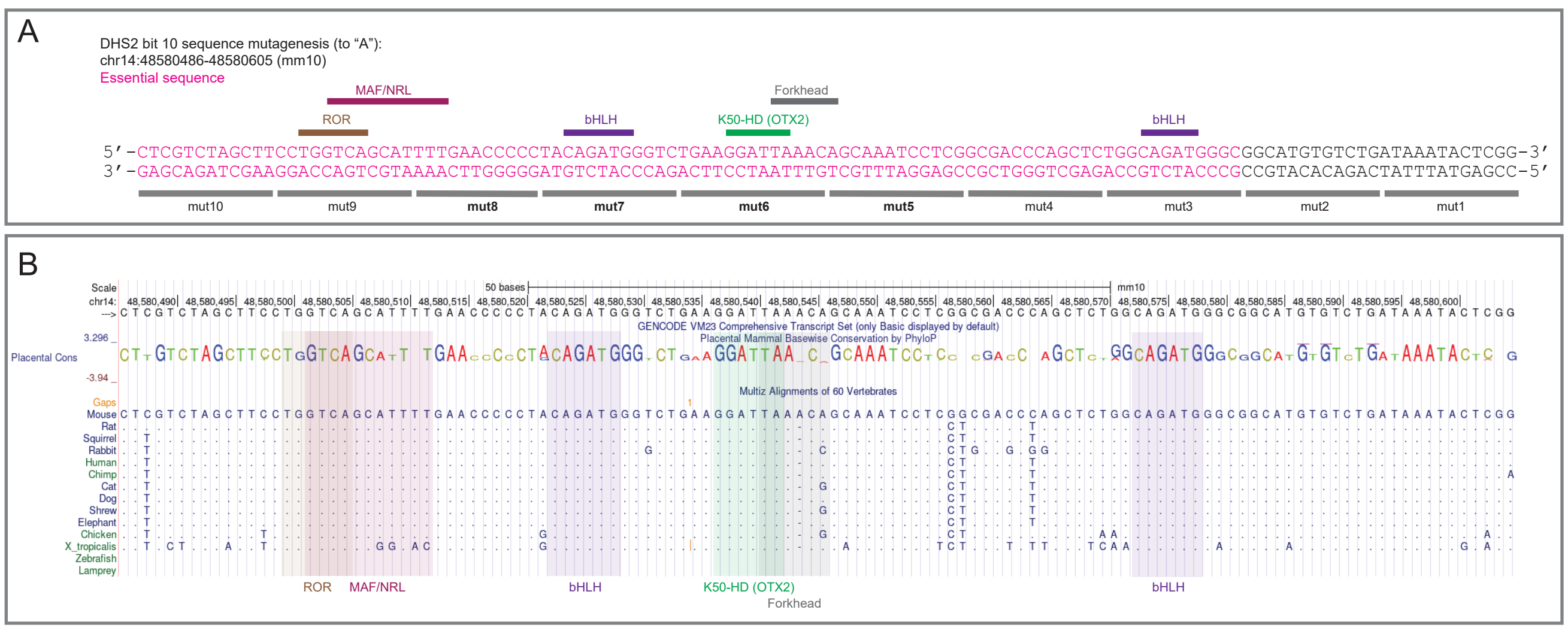

Fig. S6. Transcription factor binding predictions for the DHS2 bit 10 minimal element. (A) A subset of transcription factor binding sites predicted using JASPAR 2024 overlaid onto critical regions within the DHS2 minimal element (see also Fig. 2). **(B)** Conservation plot from UCSC genome browser with JASPAR transcription factor binding site predictions overlaid on the DHS2 minimal element. Note the high conservation of essential sequences, along with the basic helix-loop-helix (bHLH), ROR, MAF, and K50 homeodomain (HD) predicted binding sites.

Development • Supplementary information

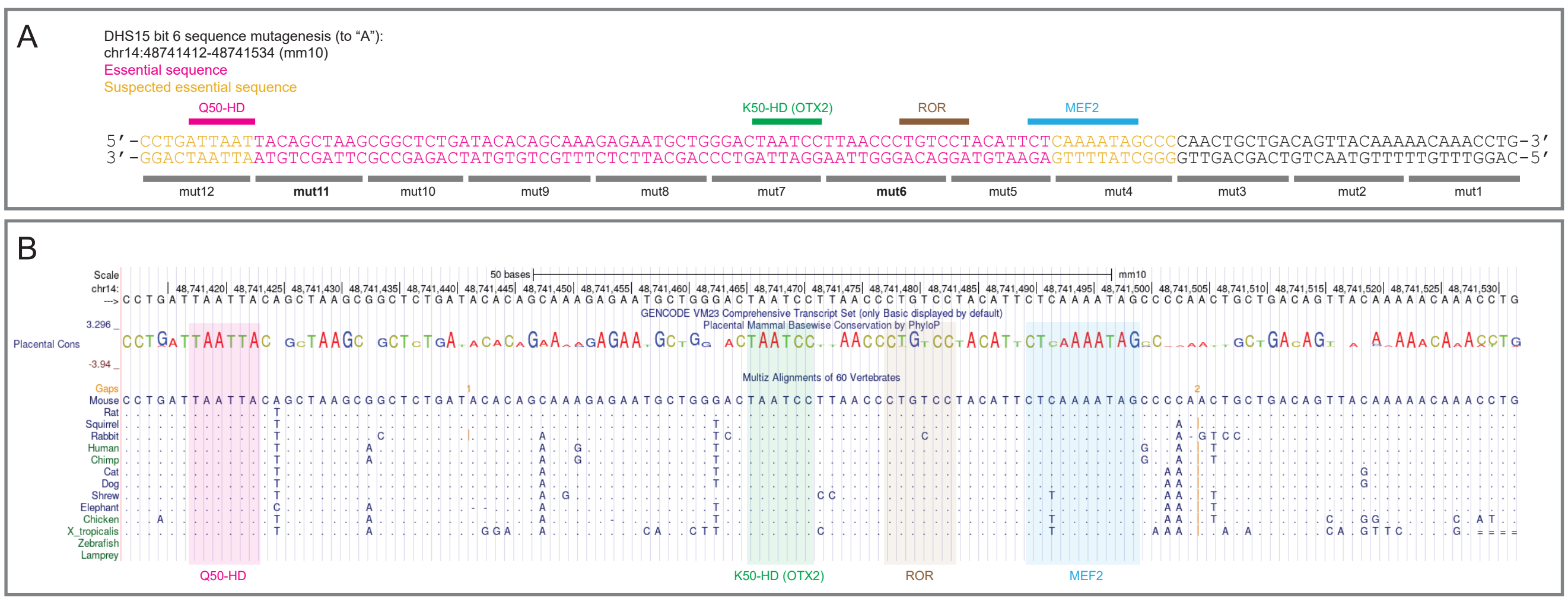

Fig. S7. Transcription factor binding predictions for the DHS15 bit 6 minimal element. (A) A subset of transcription factor binding sites predicted using JASPAR 2024 overlaid onto critical regions within the DHS15 minimal element (see also Fig. 2). Regions that showed strong, but not statistically significant, reductions in activity are highlighted in yellow as suspected essential sequences. **(B)** Conservation plot from UCSC genome browser with JASPAR transcription factor binding site predictions overlaid on the DHS15 minimal element. Note the high conservation of essential sequences, along with the ROR, MEF2, Q50 and K50 homeodomain (HD) predicted binding sites.

Development • Supplementary information

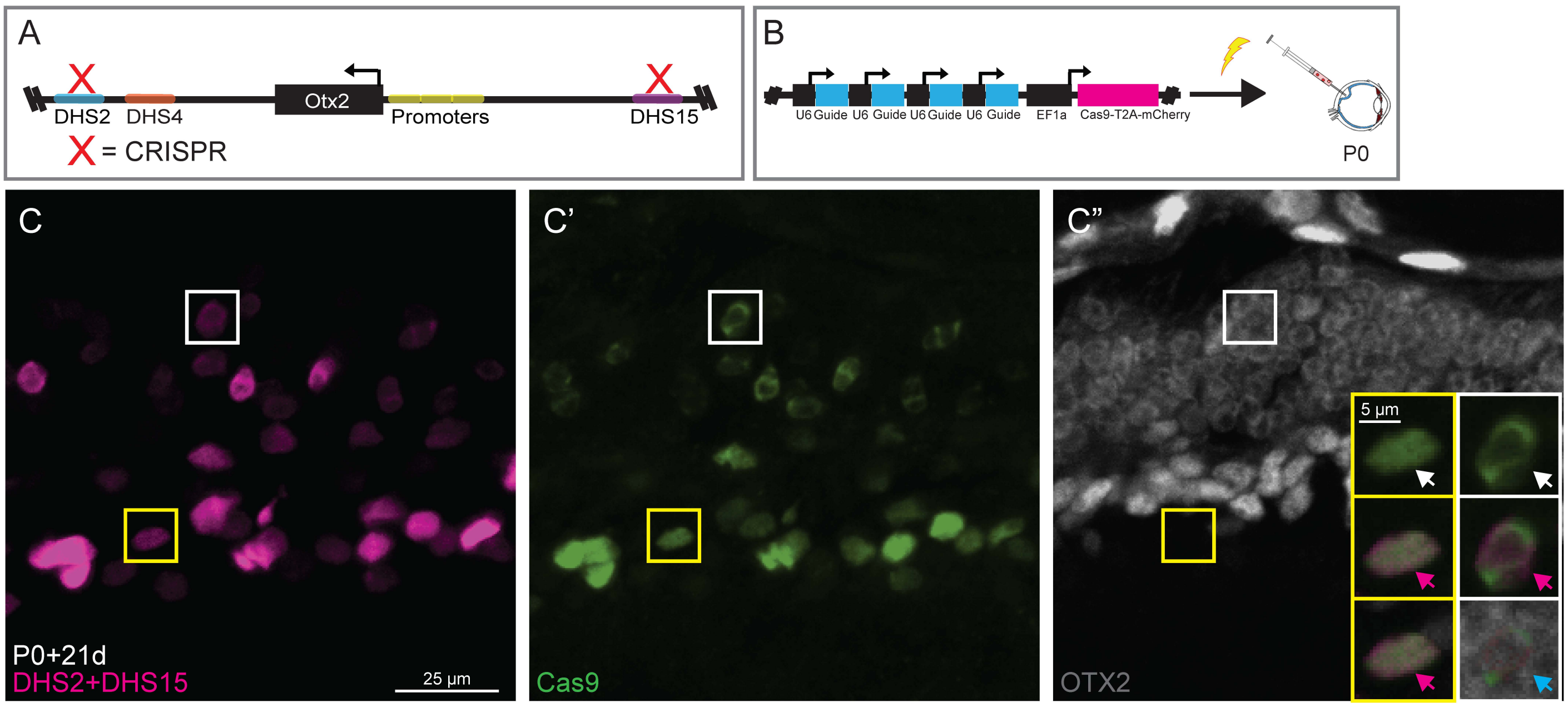

Fig. S8. Cas9 expression persists in long-term CRISPR-electroporations. (A-B) Scheme showing CRISPR targets (X) and plasmid design. Plasmids simultaneously targeting DHS2 and DHS15 were electroporated *in vivo* into P0 retinas and collected at P21. **(C-C'')** Immunostaining for mCherry, Cas9, and OTX2. Arrows mark single (white), double (magenta), and triple (cyan) labeled cells. Note that nearly all mCherry+ electroporated cells still co-express Cas9 at P21. Scale bars are 25 µm for all panels and 5 µm for insets. The outer retina is up in all panels.

Development • Supplementary information

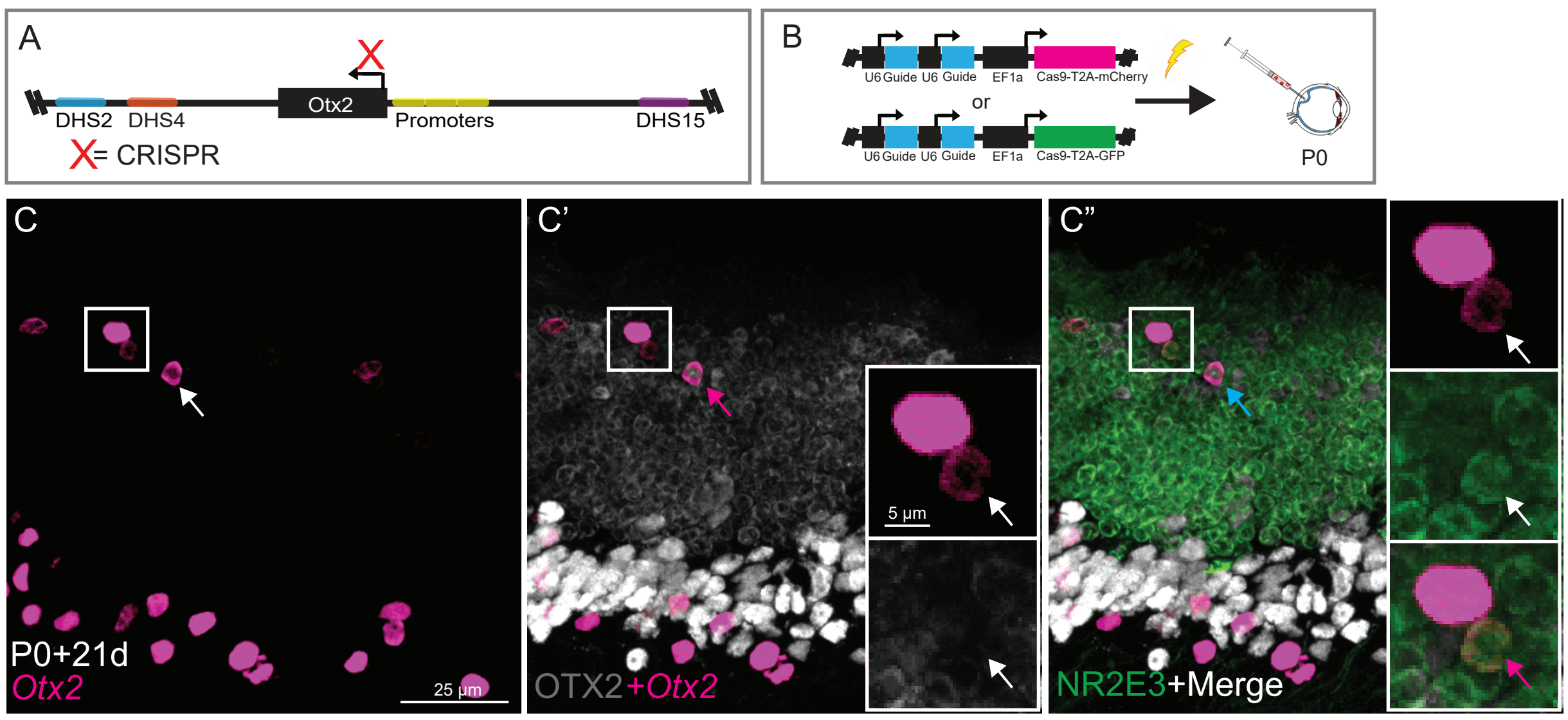

Fig. S9. Targeting *Otx2* with CRISPR results in photoreceptors lacking OTX2 expression. (A-B) Scheme showing CRISPR targets (X) and plasmid design. Plasmids simultaneously targeting the *Otx2* coding region were electroporated *in vivo* into P0 eyes and collected at P21. **(C-C")** Immunostaining for mCherry, OTX2, and NR2E3. Arrows mark single (white), double (magenta), and triple (cyan) labeled cells. While not abundant, mCherry+/NR2E3+/OTX2-negative rods were conspicuous in these conditions. Note that EF1α driven plasmids tend to show dimmer signal intensity in photoreceptors at this age, similar to the OTX2 staining pattern. Scale bars are 25 µm for all panels and 5 µm for insets. The outer retina is up in all panels.

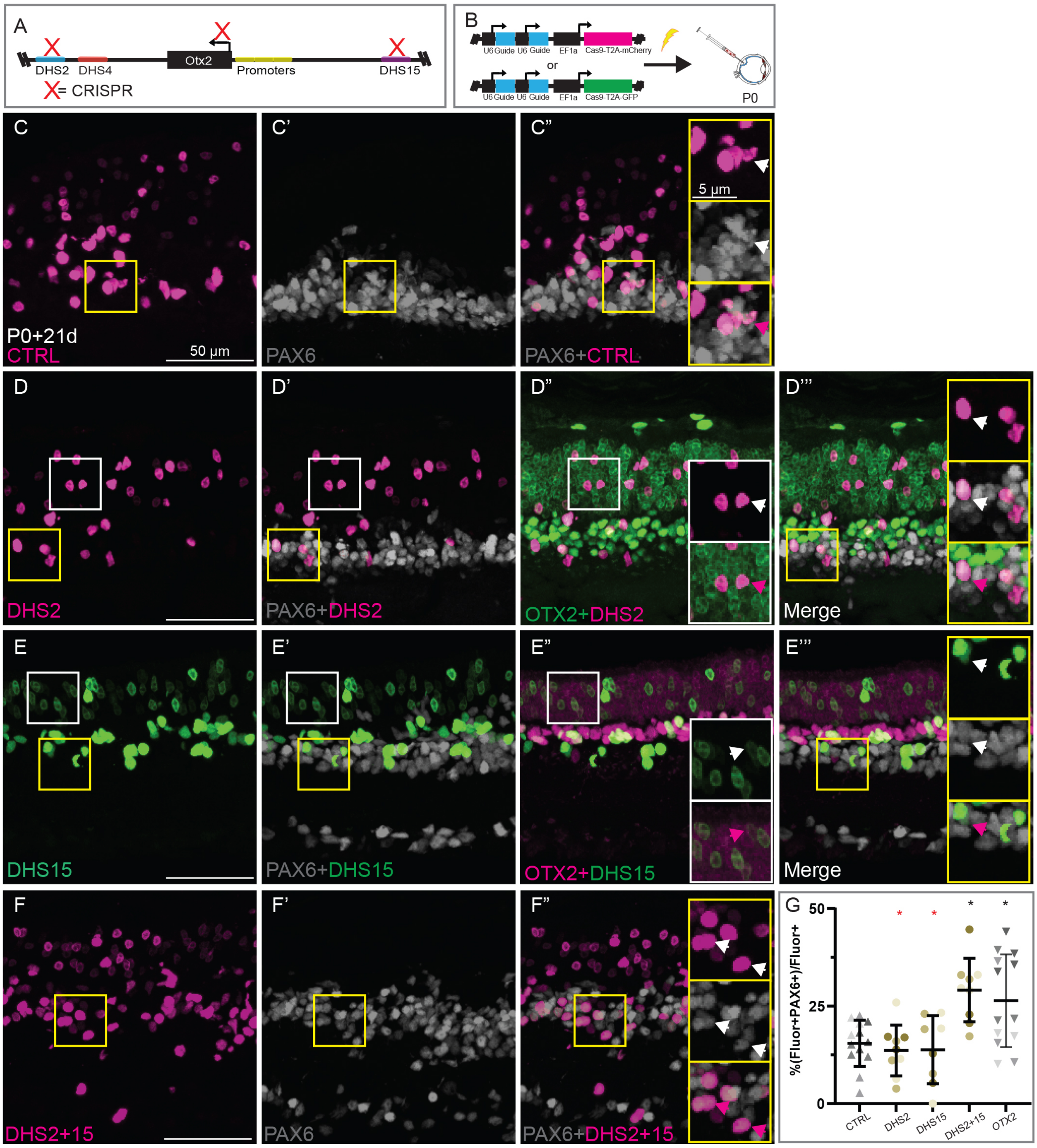

Fig. S10. CRISPR-mediated targeting of enhancers impacts PAX6+ cell numbers. (A-B) Scheme showing CRISPR targets (X) and plasmid design, as in Fig. 3. Plasmids were electroporated *in vivo* into P0 eyes and collected at P21. **(C-F")** Staining for mCherry (or GFP), OTX2, and PAX6. Note that EF1α driven plasmids often show dimmer signal intensity in photoreceptors at P21, similar to the OTX2 staining pattern. Arrows mark single (white) and double (magenta) labeled cells. Scale bars are 50 µm for all panels and 5 µm for insets. The outer retina is up in all panels. **(G)** Graph showing the percentage of electroporated cells that express PAX6 under negative control (CTRL), single CRISPR (DHS2 or DHS15), dual CRISPR (DHS2 +15), and positive control conditions (*OTX2*) at P21. For all plots, dots/triangles represent individual quantified images from a biological N ≥ 3 explants. The biological samples share the same color shade per condition. Changes were evaluated using Kruskal-Wallis non-parametric ANOVA tests. Bars show mean ± sd. Black asterisks denote P <0.05 compared to the negative control (CTRL) condition while red asterisks denote P <0.05 compared to the positive control (*OTX2*).

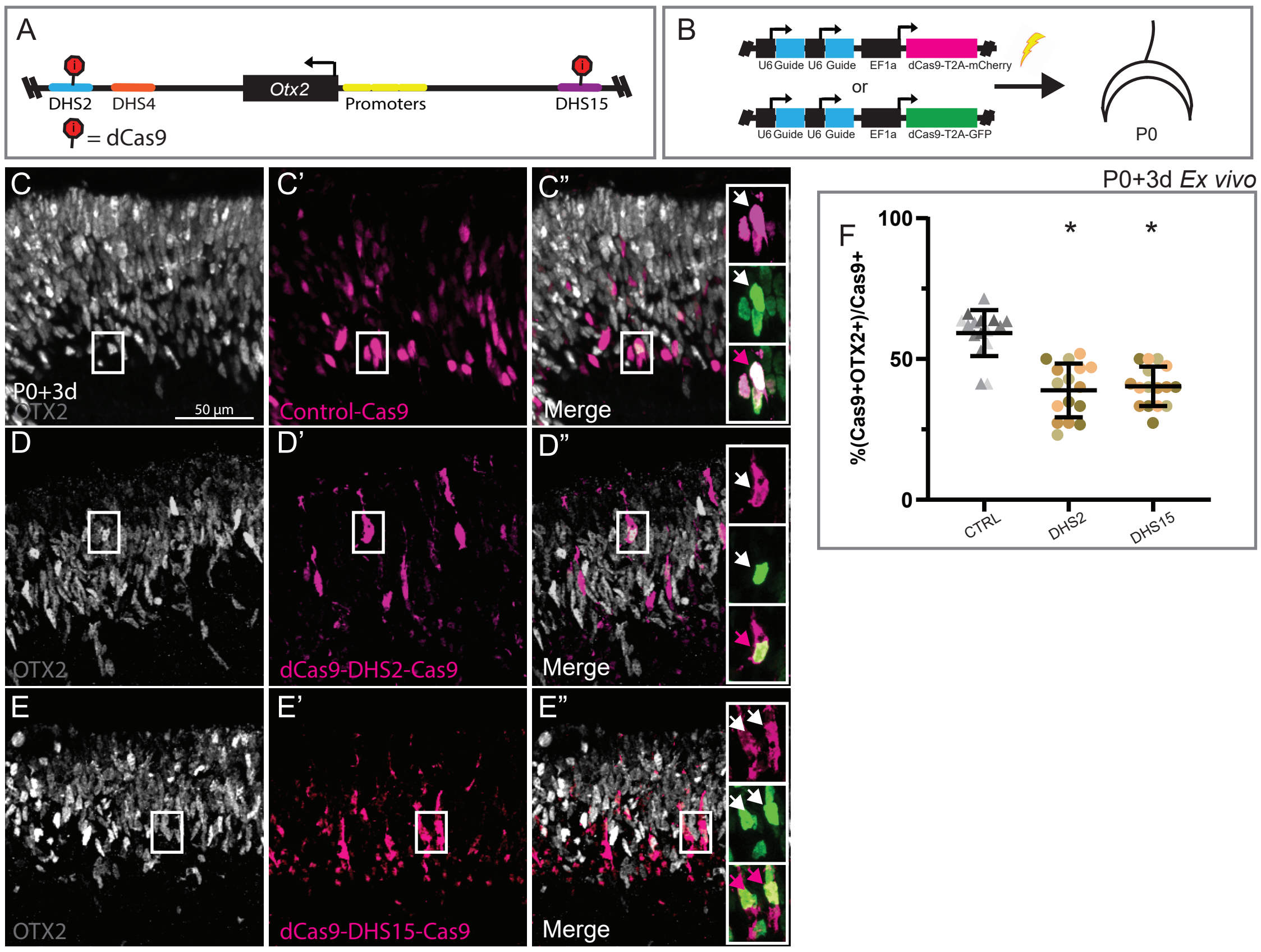

Fig. S11. Targeting DHS2 or DHS15 with non-cutting dCas9 reduces OTX2 expression. (A) Scheme showing dCas9 targets and **(B)** plasmid design. The same CRISPR guides targeting DHS2 or DHS15 were used, but in plasmids that contained only dCas9 rather than the cutting or CRISPRi versions used in Figures 3 and 5, respectively. As a negative control, a non-targeting CRISPRi construct was used (see Figs. 5-6). Plasmids were electroporated into P0 mouse retinas *ex vivo* and collected after 3 days. (**C-D"**) Immunostaining for dCas9 (magenta) and OTX2 (grey) in electroporated explants. (**E**) Quantification shows a significant decrease in the overlap of Cas9+/OTX2+ cells compared to the negative control. Insets show nuclear co-localization of dCas9 protein (magenta) and the nuclear localized fluorescent reporter (green). White arrowheads indicate single-labeled cells and magenta arrowheads indicate double-labeled cells. In the process of building the dCas9-only plasmids, one of the two nuclear localization sequences was removed. This resulted in a mix of nuclear and cytoplasmic dCas9 localization, which may reduce its potency. Scale bars are 50 µm for all panels and the outer retina is up. For all plots, dots/triangles represent individual quantified images from a biological N ≥ 3 explants. The biological samples share the same color shade per condition. Changes were evaluated using Kruskal-Wallis non-parametric ANOVA tests. Bars show mean ± sd. Black asterisks denote $P < 0.05$ compared to the negative control (CTRL) condition.

Development • Supplementary information

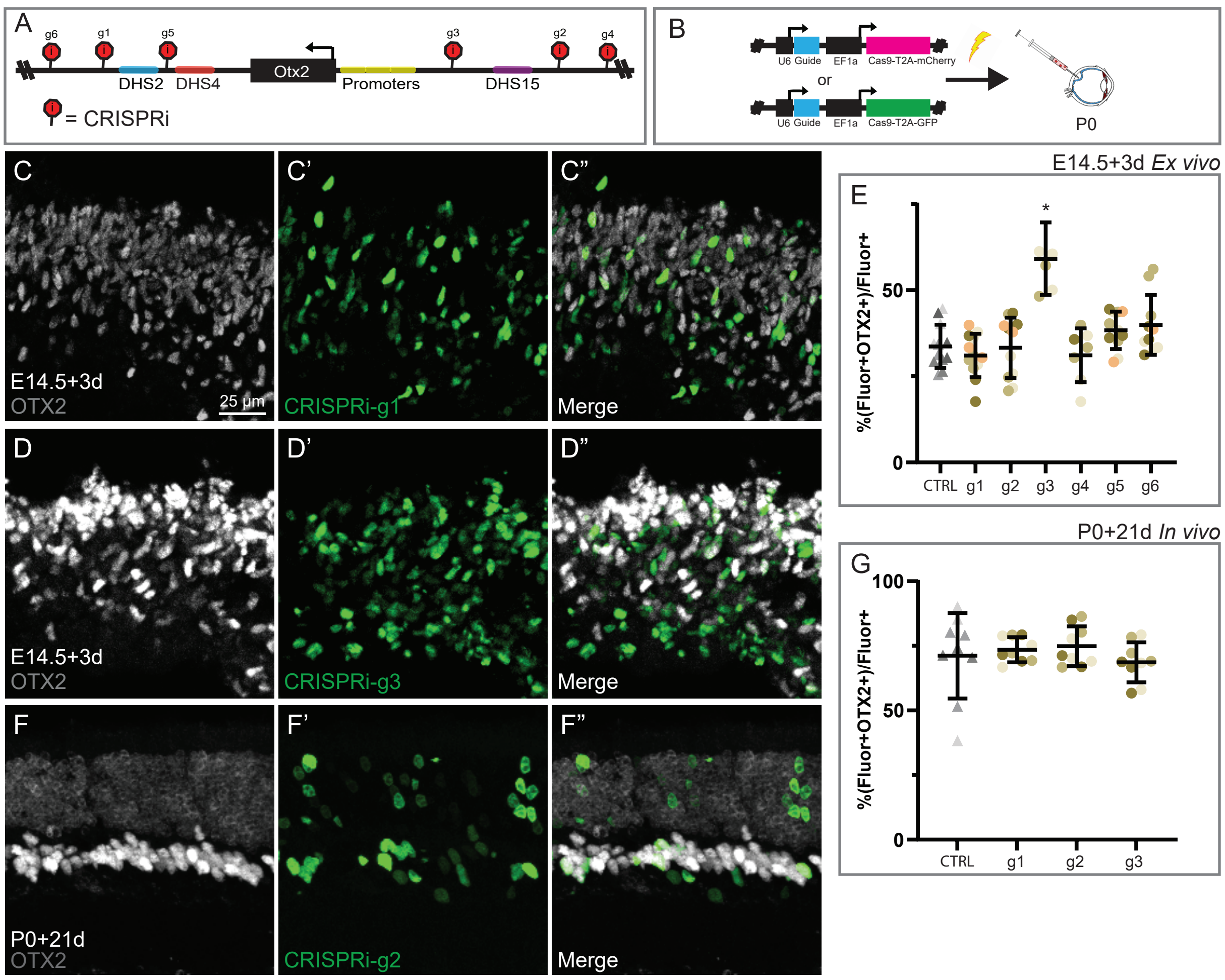

Fig. S12. CRISPRi-mediated *Otx2* repression is regionally specific. (A) Scheme showing CRISPRi targets (stop sign) and **(B)** plasmid design, as in Fig. 5. CRISPRi plasmid targeting non-coding regions (g1-g6) (one guide per location) across the *Otx2* locus were used to evaluate specificity of the technique. Plasmids were electroporated into E14.5 and P0 mouse retina *ex vivo* and *in vivo*, respectively, and collected after 3 and 21 days. **(C-D", F-F")** Immunostaining for GFP and OTX2 in E14.5 explants after 3 days of culture (C-D") or in P0 eyes collected at P21 (F-F"). Scale bars are 25 µm for all panels and the outer retina is up. **(E, G)** Graphs showing the percentage of electroporated cells that express OTX2 under negative control (CTRL) and single targeting (g1-g6) CRISPRi conditions. No loss of OTX2 expression was seen using these guides. For all plots, dots/triangles represent individual quantified images from a biological N ≥ 3 explants. The biological samples share the same color shade per condition. Changes were evaluated using Kruskal-Wallis non-parametric ANOVA tests. Bars show mean ± sd. Black asterisks denote P <0.05 compared to the negative control (CTRL) condition.

Development • Supplementary information

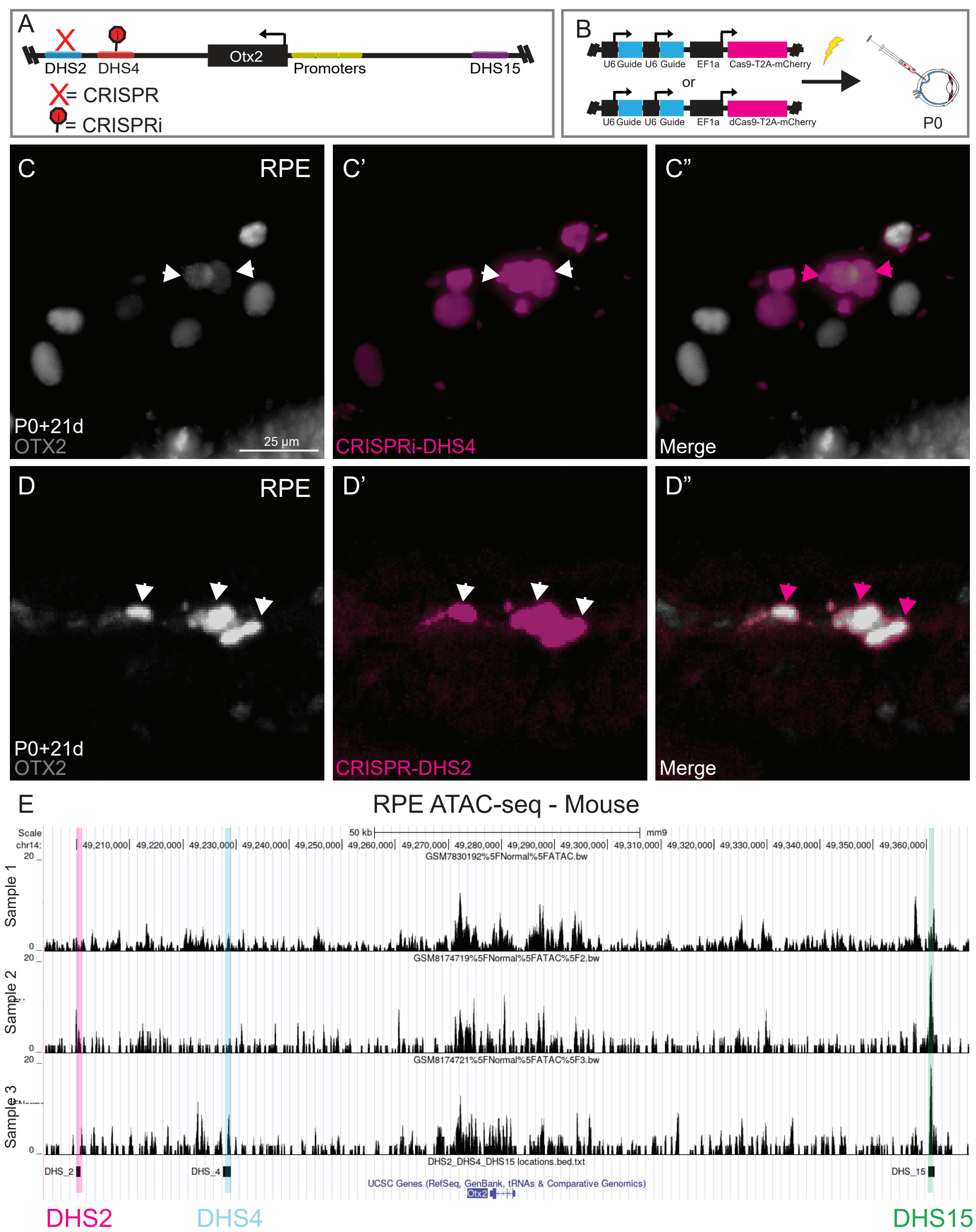

Fig. S13. CRISPR or CRISPRi targeting of the RPE does not affect OTX2 expression. (A-B) Scheme showing CRISPR (X) and CRISPRi targets (stop sign). Plasmids were electroporated into P0 mouse eyes *in vivo* and collected at P21. While these were done to manipulate the retina, on occasion electroporations targeted the RPE, which strongly expresses OTX2. **(C-D")** Immunostaining for electroporated cells (mCherry) and OTX2, focused on the RPE. (C-C") RPE electroporated with CRISPRi against DHS4, which strongly blocks OTX2 expression in the retina. DHS4 CRISPRi did not inhibit OTX2 expression in RPE. (D-D") CRISPR against DHS2 had no effect on OTX2 expression in the RPE. Arrows mark single (white) and double (magenta) labeled cells. Scale bars are 25 µm for all panels and the outer retina is up. **(E)** ATAC-seq genome browser tracks (mm9) from three mature mouse RPE samples showing chromatin accessibility across the *Otx2* locus. DHS2, DHS4 and DHS15 enhancer locations are highlighted. Accessibility is modest, with the exception of DHS15.

Development • Supplementary information

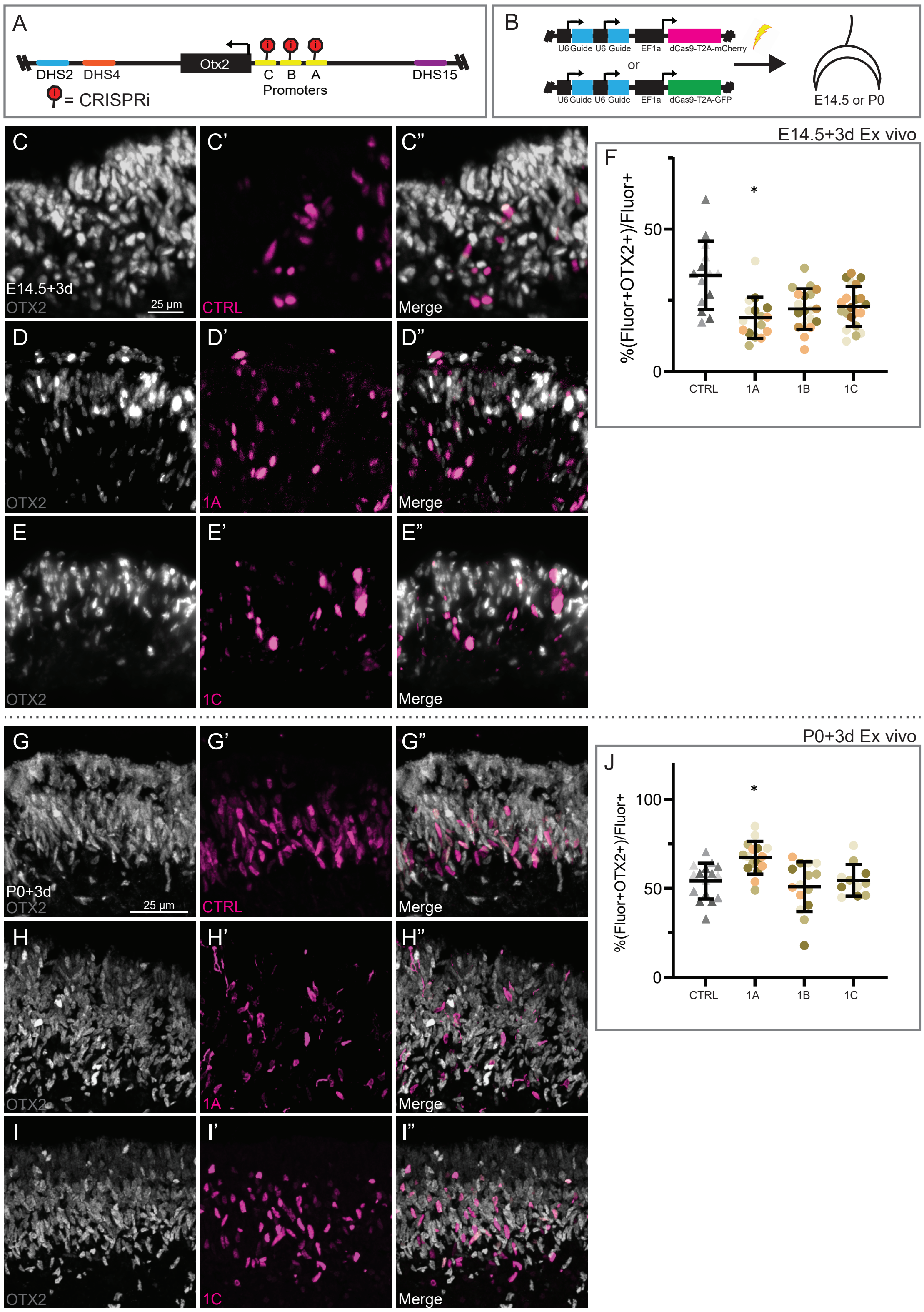

Fig. S14. CRISPRi-targeting of *Otx2* promoters has modest effects. (A) Scheme showing the location of guide sequences cloned into CRISPRi plasmids to target (stop sign) the *Otx2* promoter regions (1A, 1B, & 1C). **(B)** CRISPRi plasmids were electroporated into E14.5 and P0 mouse retinal explants and collected after 3 days of culture. **(C-E", G-I")** Immunostaining for electroporated cells (GFP or mCherry) and OTX2 from E14.5 (C-E") and P0 (G-I") electroporations. Scale bars are 25 µm for C-E" and G-I". The outer retina is up in all panels. **(F, J)** Graphs showing the percentage of electroporated cells that express OTX2 under negative control (CTRL) and single targeting (1A, 1B, & 1C) CRISPRi conditions. Significant changes in OTX2 overlap were seen only upon targeting the 1A region. For all plots, dots/triangles represent individual quantified images from a biological N ≥ 3 explants. The biological samples share the same color shade per condition. Changes were evaluated using Kruskal-Wallis non-parametric ANOVA tests. Bars show mean ± sd. Black asterisks denote P <0.05 compared to the negative control (CTRL) condition.

Development • Supplementary information

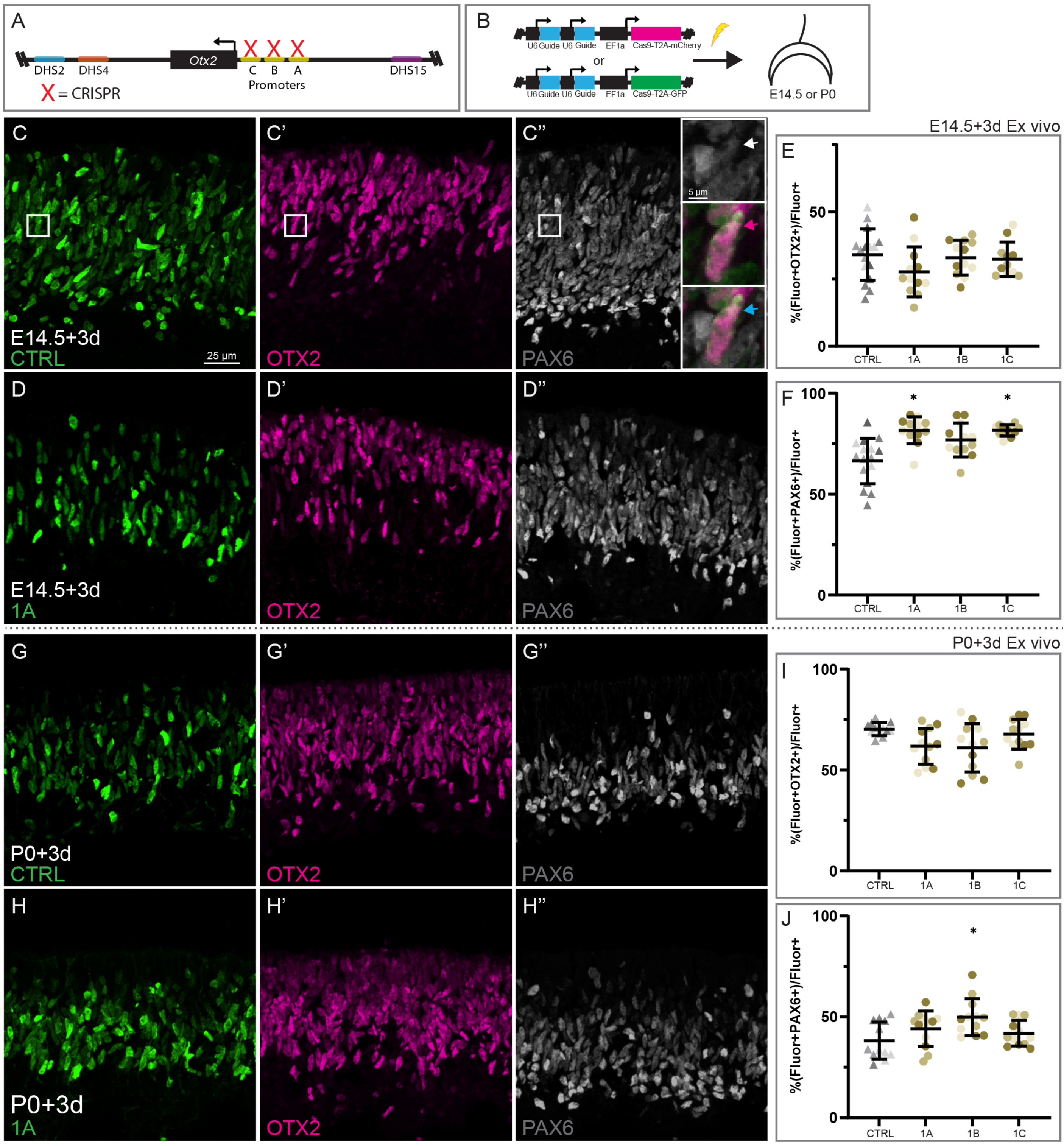

Fig. S15. CRISPR-targeting of *Otx2* promoters *ex vivo*. (A) Scheme showing the location of guide sequences cloned into CRISPR plasmids to target (X) the *Otx2* promoter regions (1A, 1B, & 1C). **(B)** CRISPR plasmids were electroporated into E14.5 and P0 mouse retinal explants and collected after 3 days of culture. **(C-D", G-H")** Immunostaining for electroporated cells (GFP or mCherry) along with PAX6 and OTX2 from E14.5 (C-D") and P0 (G-H") electroporations. Arrows mark single (white), double (magenta), and triple (cyan) labeled cells. Scale bars are 25 µm for all panels and 5 µm for insets. The outer retina is up in all panels. **(E-F, I-J)** Graphs showing the percentage of electroporated cells that express OTX2 (E, I) or PAX6 (F, J) under negative control (CTRL) and single targeting (1A, 1B, & 1C) CRISPR conditions from E14.5 (E-F) and P0 (I-J) electroporations. No loss of OTX2 expression was observed. For all plots, dots/triangles represent individual quantified images from a biological N ≥ 3 explants. The biological samples share the same color shade per condition. Changes were evaluated using Kruskal-Wallis non-parametric ANOVA tests. Bars show mean ± sd. Black asterisks denote P <0.05 compared to the negative control (CTRL) condition.

Development • Supplementary information

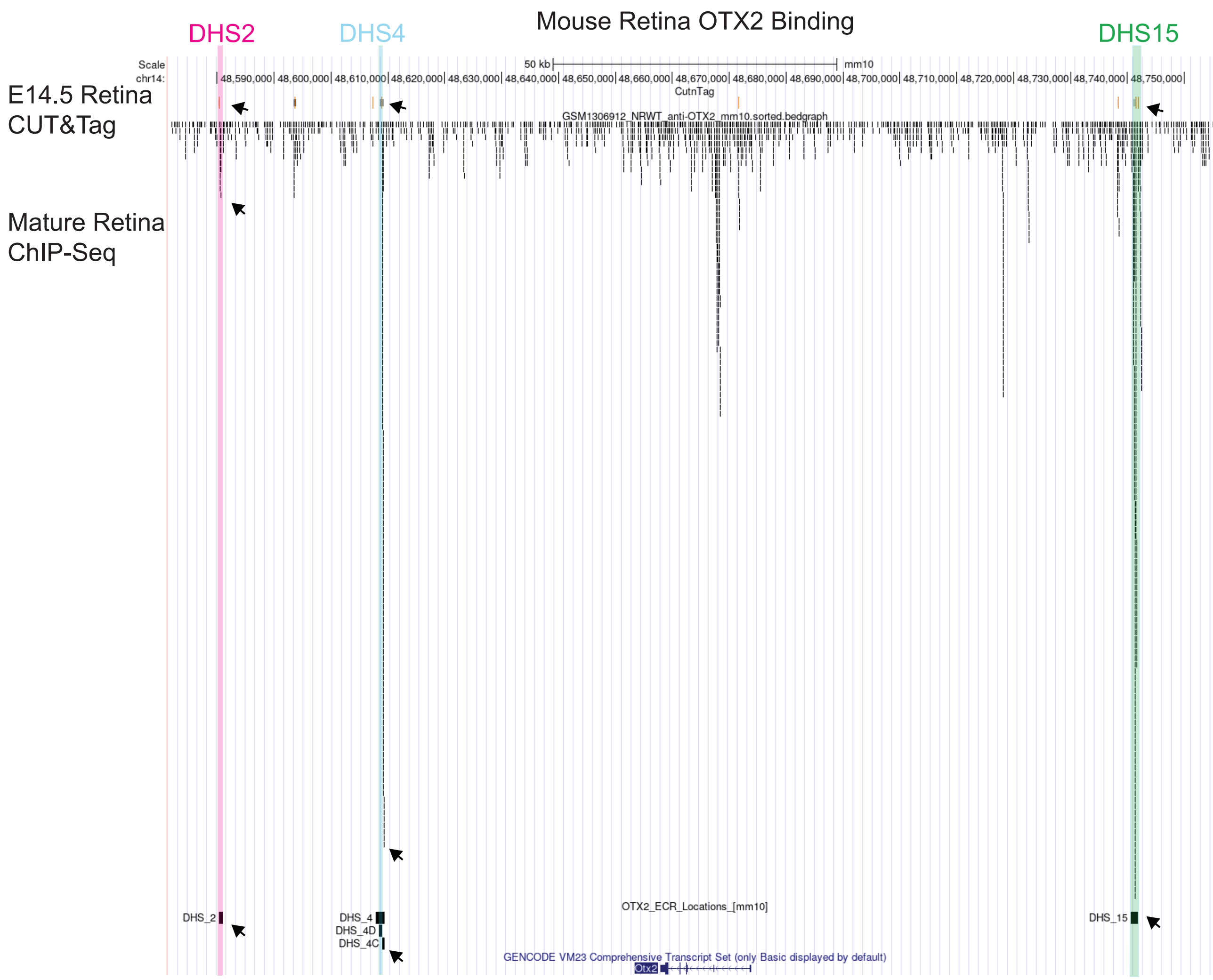

Fig. S16. CUT&Tag and ChIP-seq datasets suggest that OTX2 regulates DHS15. UCSC genome browser track (mm10) from E14.5 cleavage under targets and tagmentation (CUT&Tag) and adult retina OTX2 chromatin immunoprecipitation sequencing (ChIP-seq) datasets. The DHS2, DHS4, and DHS15 regions are highlighted. Arrows highlight signal overlap between enhancer regions and the CUT&Tag and ChIP-seq signals. Modest overlap is seen with the DHS2 region and the overlap with DHS4 is outside of the essential D-region, instead overlapping with DHS4-C, which lacks appreciable activity in the retina. Strong overlap is seen with DHS15, suggesting that OTX2 positively regulates itself through DHS15.

Development • Supplementary information

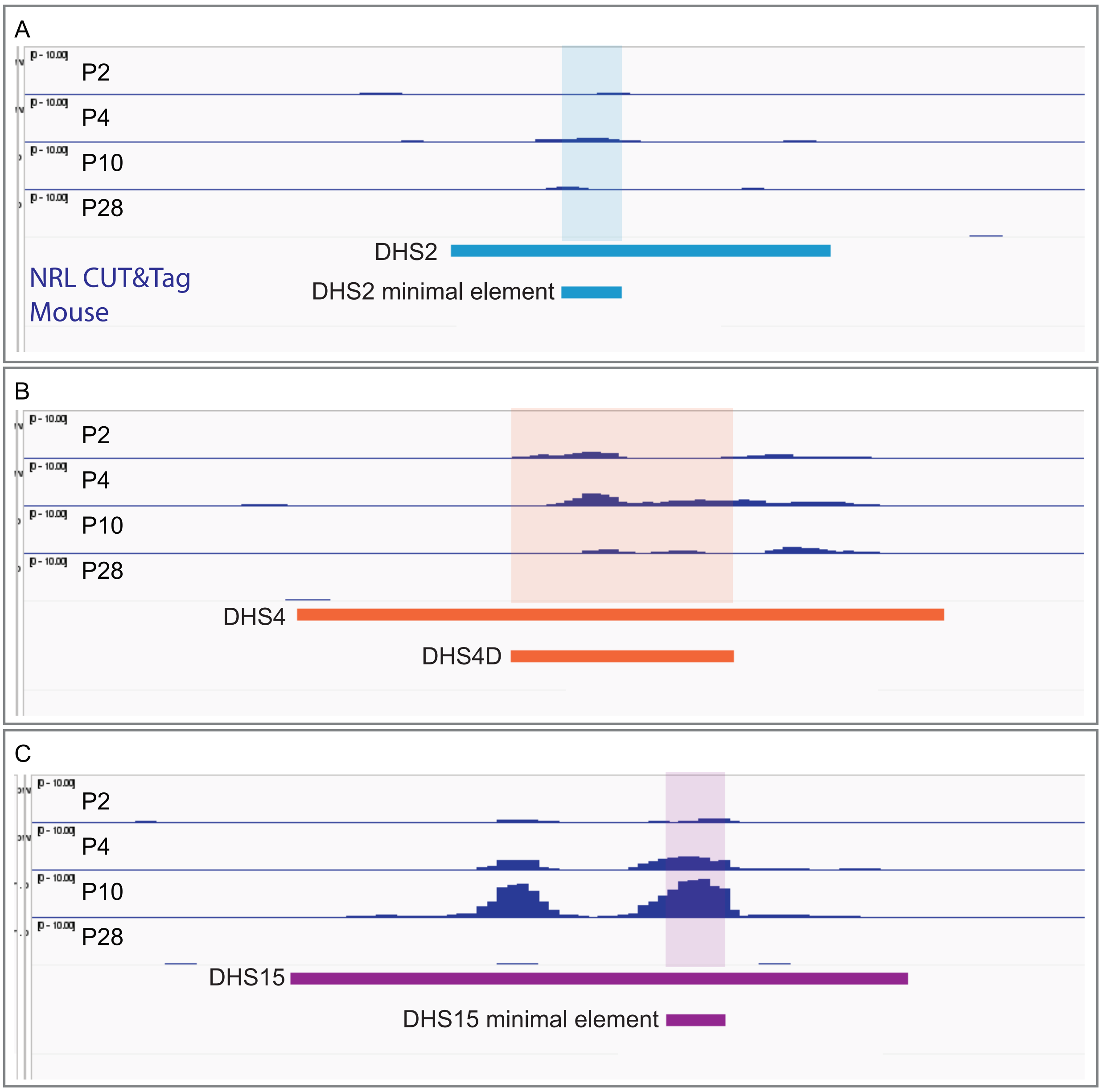

Fig. S17. CUT&Tag datasets suggest that NRL regulates DHS15 in the maturing murine retina. IGV browser showing the location of **(A)** DHS2, **(B)** DHS4 and **(C)** DHS15 enhancers. NRL CUT&Tag peaks at P2, P4, P10 and P28 are shown in each panel. Across development, NRL binding is either weak or absent at DHS2 and DHS4. Starting at P4 and increasing at P10, NRL binding is evident at DHS15. No NRL binding was observed at P28 at any of the enhancers.

Development • Supplementary information

Table S1. List of JASPAR predicted binding sites for DHS2 and DHS15 minimal enhancers.

Available for download at
https://journals.biologists.com/dev/article-lookup/doi/10.1242/dev.204881#supplementary-data

Table S2. List of enhancer sequences, guides, and antibodies used.

Available for download at
https://journals.biologists.com/dev/article-lookup/doi/10.1242/dev.204881#supplementary-data

Table S3. Mutation spectrum analysis in retinal cells electroporated with conventional CRISPR guides.

Available for download at
https://journals.biologists.com/dev/article-lookup/doi/10.1242/dev.204881#supplementary-data
