# Supplementary material for: A robust cis-regulatory network ensures Otx2 expression during retinal development
Source: Development. 2026 Mar 27;153(6):dev204881. doi: 10.1242/dev.204881 (PMC13495790; doi:10.1242/dev.204881)
Supplement: Supplementary file 2 [file develop-153-204881_review_history.pdf]

A robust *cis*-regulatory network ensures *Otx2* expression during retinal development

Ian J. Purvis, Omar E. Ochoa Olmos, Ko Uoon Park, Michael L. Kaufman, Charles M.Henry, Chris Schaaf, Olivia J. Clise, Corey D. Tesdahl, Ami Haas and Joseph A. Brzezinski, IV
DOI: 10.1242/dev.204881

Editor: François Guillemot

Review timeline

Original submission:	23 April 2025
Editorial decision:	6 June 2025
First revision received:	22 October 2025
Editorial decision:	27 November 2025
Second revision received:	9 February 2026
Accepted:	13 February 2026

Original submission

First decision letter

MS ID#: dev.204881

MS TITLE: A robust cis-regulatory network ensures Otx2 expression during retinal development

AUTHORS: Ian Purvis, Omar Ochoa Olmos, Ko Park, Michael Kaufman, Charles Henry, Christopher Schaaf, Olivia Clise, Corey Tesdahl, Ami Haas and Joseph A. Brzezinski IV

I have now received the reports of three referees on your manuscript and I have reached a decision. The referees' comments are appended below, or you can access them online: please go to:

As you will see, the referees express great interest in your work but they also have some significant criticisms and recommend a substantial revision of your manuscript before we can consider publication. If you are able to revise the manuscript along the lines suggested, which may involve further experiments, I will be happy receive a revised version of the manuscript. Your revised paper will be re-reviewed by one or more of the original referees, and acceptance of your manuscript will depend on your addressing satisfactorily the reviewers' major concerns. Please also note that Development will normally permit only one round of major revision.

If it would be helpful, you are welcome to contact us to discuss your revision in greater detail. Please send us a point-by-point response indicating your plans for addressing the referees' comments, and we will look over this and provide further guidance.

Please attend to all of the reviewers' comments and ensure that you clearly highlight all changes made in the revised manuscript. Please avoid using 'Tracked changes' in Word files as these are lost

© 2026. Published by The Company of Biologists under the terms of the Creative Commons Attribution License (https://creativecommons.org/licenses/by/4.0/).

in PDF conversion. I should be grateful if you would also provide a point-by-point response detailing how you have dealt with the points raised by the reviewers in the 'Response to Reviewers' box. If you do not agree with any of their criticisms or suggestions please explain clearly why this is so.

Reviewer 1

Advance summary and potential significance to field

The manuscript of Purvis et al. examines the regulation of the key retinal development transcription factor Otx2 through the action of three regulatory elements at the locus. The study investigates an important question in developmental biology, which is how the expression of critical developmental regulator genes is precisely controlled and robust. The use of CRISPRi (CRISPR interference) to target enhancers leads to more penetrant phenotypes which suggests that the use of this technique may be disrupting some fundamental aspect of gene regulation such as enhancer interaction. This is one of the main advances though the mechanism is not clear at this point and this would strengthen the conclusions of the study. There are a number of rigorous approaches and high quality data in this manuscript but there are some key points that should be addressed to make the conclusions more specific and well-supported.

Comments for the author

Major issues

1. One issue is whether the conclusions are fully supported for some of the data. There are a few examples where the effects are relatively small or not fully quantified where it makes firm conclusions hard to justify. The study does have some good quantitation, but here are other examples that fall into this category.

A) In Fig1, reporters for DHS2 and DHS15 are co-electroporated and it is concluded that the majority of cells use these enhancers simultaneously. However, the individual reporter patterns at E14.5 especially, but also at P0, look qualitatively different from one another (In B, there are a lot of DHS2-active cells near the RGC layer and not nearly as many DHS15-active, for example). This conclusion would be firmer with quantitation and/or with controls of retinas co-electroporated with the same enhancer driving the two fluorophores (DHS2-GFP + DHS2-cherry, for example) to determine what amount of deviation reflects the experimental set-up. In Supp Fig2, the overlap between DHS2 and DHS4 reporters as well as DHS15 and DHS4 reporters looks very concordant. It is not clear how this is squared with what is observed in Fig1 where it would then be expected for DHS2 and DHS15 to be more concordant with each other.

B)The study uses Rxrg staining to identify cells in FigS3, but the Rxrg staining is not very localized to the top part of the retina as you would expect, perhaps because the retinas are cultured for 10 days - perhaps a shorter time period would be sufficient to preserve morphology and also see activity? There is not also any quantitation so with the disorganized retina it is hard to determine if there is any specificity for cone cells.

C) Has a control condition been performed to determine if the Cre vector has any basal activity for Rosa reporter induction when a form without an enhancer is introduced? If this is shown in one of the previous studies that used it, this would be important to reference.

D) Experiments using Crispr guides to target the three different promoters are done and no Otx2 loss-of-function phenotypes are observed. The conclusion is that the promoters are interchangeable, but what is the evidence that the guides are actually effective at targeting the promoters? If they aren't actually effective than that could be the reason for this negative effect? Can an assessment be made that the guides are effective? The same argument could be made for the Otx2 coding region CRISPRi (line 351-) and the SuppFig10 data on the random Otx2 control regions. The authors do note generally that the effectiveness of the CRISPR reagents is a limitation of the study at the end of the paper.

© 2026. Published by The Company of Biologists under the terms of the Creative Commons Attribution License (https://creativecommons.org/licenses/by/4.0/).

E) Fig S8 - there is one example of a cell lacking Otx2 that supports the conclusion that a photoreceptor can survive without Otx2- How often is this observed? Could it be in the process of dying? With the use of a cytoplasmic GFP reporter could one determine that it has normal morphology and test for other photoreceptor markers?

2. The use of DHS2 and DHS15 CRISPR guides to target these elements individually shows relatively moderate effects, but the use of the DHS2 and DHS15 guides together leads to stronger effects which suggests that the elements may be redundant with each other (Line 294-). But since DHS2 and DHS15 are located flanking the Otx2 gene, couldn't it be that the combined use of these guides leads to deletion of the entire Otx2 coding region which is the reason for the phenotype?

3. The interpretation of the CRISPRi results is an important part of the paper as they are interpreted to show that the enhancer elements are interacting with each other. One instance where this is raised is with regards to DHS4 CRISPRi line 342-. "This perturbation acted like a dominant negative" - not sure what is meant by this? It seems likely that the authors are making an analogy to the genetic definition of a dominant negative allele whereby the mutant copy also is able to decrease the function of the product of the wild-type allele. Not sure if that analogy holds here in that the CRISPRi is likely targeting both alleles already and the extra effect may be on some other distinct genetic component, like another enhancer. Clarification on what is meant by dominant negative and discussion of how this particular term is applicable to this situation would be helpful.

It is also suggested that the CRISPRi was much stronger than conventional CRISPR. The suggestion on line 348 is that this is consistent with the CRISPRi affecting more than just its targeted enhancer. But why couldn't it be that the CRISPRi is just more effective at silencing the enhancer?

The discussion has several good points about why CRISPRi may be more efficacious than conventional CRISPR and how this may be due to interactions with other enhancer elements at the locus. However, there isn't a mechanism identified and so there is not a clear conclusion that can be drawn about what these results mean in terms of normal gene regulation.

4. The two enhancers of focus (DHS2 and DHS15) are active in similar cell populations but suggested to have different upstream regulation (at least to some extent) because the sequence motifs that are present in critical regions are different. The motifs correspond to predicted regulation by certain transcription factors, but there is not follow-up to show that modulation of these transcription factors (CRISPRing them or misexpressing them, for example) have differential effects on the two elements. That would make for a more solid case that these elements are in fact regulated differently.

Minor

Line 251,2 - "positive control plasmids targeting the Otx2 coding region remain highly efficacious at P7" - it is not clear what this means.

In mutants for Fig 2 and S4 it is not clear if there is a change in specificity if the amount of expression is very low. Then perhaps most of the GFP expression is from the basal activity of the vector and that is not likely to be Otx2-enriched and so it looks like it is not specific? It would be helpful if this interplay of specificity and activity was discussed for low activity situations.

Line 146 - suggest that the number of bipolar cells was variable and maybe because the elements mark only a subset of the bipolar population - not sure that is a valid argument as the the bipolar subtype percentages are pretty consistent across biological replicates?

© 2026. Published by The Company of Biologists under the terms of the Creative Commons Attribution License (https://creativecommons.org/licenses/by/4.0/).

Reviewer 2

Advance summary and potential significance to field

OTX2 plays a crucial role in specifying retinal cell fates, influencing the formation of about 85% of retinal cells. Its loss results in the absence of photoreceptors and bipolar cells and an increase in other cell types. Otx2 is activated in progenitor cells just before they exit the cell cycle and remains expressed in mature photoreceptors and bipolar cells, but not in amacrine or horizontal cells. Of the three enhancers (DHS2, DHS4, DHS15) identified previously, DHS4 is shown to initiate Otx2 expression. In this manuscript, the authors examined the function of DHS2 and DHS15 by matched CRISPR/Cas9 and CRISPRi targeting. The authors concluded that multiple cis-regulatory elements interact and substitute for each other to ensure robust OTX2 expression to specify retinal cell fates.

Overall, this is an excellent study that complements previous research and offers new insights into Otx2 expression during retinal development. The data being presented is definitely significant. The potential underlying mechanisms for the regulation have been discussed and limitations of the study acknowledged. However, the data does not completely justify the conclusions. Additionally, the experimental design is rather linear and alternate explanations exist. Otx2 gene likely has multiple cis-regulatory elements (CREs) given its key role in determination of multiple retinal cell fate. Could it serve as a competence factor for different fates but may not determine any on its own? HiC data at high resolution may be necessary to dissect Otx2 regulation in distinct spatiotemporal contexts. If mouse data is not available, can the authors get some insights from human HiC data? The authors are generally careful but have not discussed additional interpretations and experiments that could test. Some figures lack clarity as well. Discussion is long and redundant. The authors should primarily discuss key findings, integrating these with what is published. There is no reason to restate the results.

Specific comments and suggestions:

1. Summary statement, Abstract and text: "cis-regulatory robustness" - unclear what this really means? Is it "precision"? Also, is flexibility same as redundancy or are they talking about complementarity or synergy?

2. Abstract and text: "Each enhancer is needed for normal Otx2 expression." Quantitative statements are difficult based on immunostaining and flurescence methods. Additionally, recent studies have shown that the concentration of a transcription factor is critical in specific contexts. Same factor can have distinct functions, interaction with other proteins, or binding rate/affinity when present in different concentrations.

3. It is difficult to assume that Otx2 must be expressed at same levels in all cells and at all times, as implied by the last sentence in Abstract.

4. It's unclear how the authors evaluated the efficiency of the guide RNAs used in the study.

5. The authors should incorporate the cell type specificity of Otx2 enhancers (DHS2 and DHS15) from published single-cell ATAC-seq data (PMID: 34788628). Please incorporate any Hi-C and K27Ac data if available since these data would be critical for obtaining a more comprehensive overview of cis-regulatory elements that dictate Otx2 expression.

6. Throughout the manuscript, cell fate change was evaluated by CRISPR targeting from P0 onward. However, the fate of all cones and 10% of rods is already specified before that and would likely escape CRISPR engineering. Without additional studies at early time points, can the authors draw broad conclusions? This limitation of the experimental design should be included in Discussion. How did the authors identify photoreceptor vs bipolar cells? What cell markers were used?

© 2026. Published by The Company of Biologists under the terms of the Creative Commons Attribution License (https://creativecommons.org/licenses/by/4.0/).

7. Based on plasmid-based reporter assay in explant cultures, both DHS2 and DHS15 have enhancer activity at embryonic day embryonic day (E) 14.5 and postnatal day (P) 0. However, in-vivo assay on DHS2 and DHS15 from previous study (Wilken et al., 2015) suggest low expression of both the enhancers at P0 and increasing later at P7. Therefore, based on the previous report the DHS2/DHS15 enhancers should have no or limited expression at embryonic day. Please clarify.

8. In Fig 1B-C, many OTX2+ cells do not appear to express either plasmid, and many cells expressing DHS2 or DHS15 don't express OTX2. This can be better depicted and explained. Additionally, the expression of both individual and co-expressed reporter plasmids for DHS2 and DHS15 should have a separate figure.

9. Similarly, in Fig E-F, many OTX2+ photoreceptors and bipolars are not labeled with Rosa-RFP and DHS2 or DHS15. How is OTX2 expression maintained without DHS2 or DHS15 activities?

10. The authors hypothesize "DHS4+ cells would transition towards using DHS2 and DHS15 to maintain OTX2 expression". Based on the results DHS2 and DHS15 are simultaneously active at E14.4 and P0. It does not provide convincing evidence for transition of DHS4+ cells. Perhaps other enhancers are involved. Additionally, the authors should incorporate published data (PMID: 31493975, PMID: 28472656) to map poised active status of the DHS2 and DHS15 in developing mouse retina.

11. Otx2 is expressed even in mature retina though it's expression pattern changes. Can the authors perform reporter assays similar to figure 1 for DHS2 and DHS15 in adult mouse retina? The activity of most cell-type specific enhancers is spatiotemporally controlled.

12. In Fig S3, why did the authors specifically focus on cone photoreceptors?

13. In Fig 3, which panels are showing single electroporation of DHS2 or DHS15, or co-electroporation of both plasmids? Looks like panel D and H are showing co-electroporation but many cells are expressing only mCherry or GFP but not both? Did the authors consider these cells as single deletion of DHS2 or DHS15? The authors can use single electroporated samples.

14. In Fig 4, immunostaining images for DHS15 mutation at 7d were not included. Similarly, images for single mutation of DHS2 or DHS15 at 21d were not included. Fig 4B and Supp Fig 9B should include the design of a single plasmid expressing all 4 gRNAs.

15. Did the authors think different cell types may response to the loss of DHS2 or DHS15 differently? In Fig 4F and J, the authors may quantify OTX2 expression in photoreceptor and bipolar cells separately.

16. Authors have used JASPER to predict transcription factor binding for essential regions of DHS2 (Fig. S5) and DHS15 (Fig. S6). Published results of mapping retina specific TFs using ChIP-seq/CUT&RUN on mouse retina should be incorporated to validate the differential binding of retina specific TFs on DHS2 and DHS15.

17. "Targeting DHS2 and DHS15 significantly reduced OTX2 overlap (~15%), but this effect was modest compared to P0 retinas examined after 3 days (~50%) and the positive control condition (~75%)". Did the authors profiled for other cell types which might be responsible for modest phenotype at P7?

18. Line 296, the authors stated that "our results suggest that DHS2 and DHS15 partially substitute for each other to enable OTX2 expression and photoreceptor and bipolar cell formation". While panel 4K shows the ratio of photoreceptor vs bipolar is not changed, how about the absolute number of photoreceptor cell and bipolar? Did the authors suggest a cell fate shift from photoreceptor/bipolar to amacrine cell?

19. Fig 5 should include CRISPRi images against DHS15.

20. In Fig 6, the authors should explain why CRISPRi targeting of DHS2, DHS4, and DHS15 shifted the distribution of electroporated cells towards the inner nuclear layer and again should use cell type markers to evaluate photoreceptor/bipolar ratio. Was the unchanged ratio due to the inefficiency in targeting? In Supp Fig 10, the authors should show the images of g3 at E14.5 since it led to increased OTX2 expression.

21. Fig S14 can be moved to Results section rather than Discussion.

© 2026. Published by The Company of Biologists under the terms of the Creative Commons Attribution License (https://creativecommons.org/licenses/by/4.0/).

22. Line 453: the authors state that "we did not observe NRL ChIP signal at this enhancer (Hao et al, 2012)". NRL ChIP-seq was performed using adult retinas. The authors should check for NRL Cut&Run data at Otx2 during retinal development (PMID: 35776116)

Reviewer 3

Advance summary and potential significance to field

Previous studies from the senior author and others have identified enhancers that control temporal and cell-state dependent elements of Otx2 expression. Otx2 is a critical transcriptional regulator of retinal development, and the spatio-temporal regulation of its expression is complex and not fully understood. In this study, Purvis and coauthors perform a detailed assessment of enhancer properties in vivo and ex vivo contexts using electroporation of developing mouse retina. The study incorporates carefully designed enhancer testing to identify required minimal sequences. Mining of published ATAC-seq and Chip-seq datasets enabled predictions of candidate interacting transcription factors although they were not the primary focus. Rather, the team used CRISPR/Cas9 mutation and CRISPRi testing to test the candidate regulatory sequences within the enhancers and to identify potential interactions between them. Three enhancers were the primary focus, DHS4, which activates Otx2 expression in retinal progenitors and DHS-2 and -15 important for maintained expression in differentiating and mature photoreceptors and bipolar cells. By using both CRISPR and CRISPRi, the authors show that DHS-2 and -15 have overlapping and unique characteristics. Interestingly, CRISPRi targeting of the key enhancer sequences had stronger effects than CRISPR targeting at the same locations. CRISPRi testing of sequences near, but not part of the enhancers had minimal effects, which demonstrated specificity of these effects. Through iterative testing of these enhancers in isolation and in combination, their requirements to regulate Otx2 were shown to not be absolute but still important for the complex spatio-temporal regulation of Otx2 in the retina. While some effects on cell fate choice were observed with CRISPR targeting, the effects were more pronounced with CRISPRi. How this occurs is not clear, but the effects were convincing and reveal a robustness of the Otx2 enhancer environment to regulate Otx2. It is my opinion that this is a significant contribution to understanding how transcription factor expression is regulated in complex developmental programs such as that in the retina.

Comments for the author

The manuscript was well organized, experiments were well-controlled, and sample sizes appeared to be sufficient and analyzed with appropriate statistics. The literature was well cited. One issue was that the results and discussion were repetitive in several areas and editing would improve the discussion's readability.

The authors make the argument that the CRISPRi is having more potent effects than CRISPR/Cas9 targeted sequences because steric hindrances are interfering with the flexibility and interactions of the enhancers. This explanation is hard to follow. Is the point that the CRISPRi protein complex is causing the steric hindrances between the interacting enhancers? I could imagine two other possibilities: that bringing a transcriptional repressor to an enhancer will have a dominant negative effect as proposed for DHS4, or that binding of the CRISPRi complex is displacing more transcription factors at the enhancer than the CRISPR/Cas9 mutant.

There are several figures, especially in the supplement that have saturated signal, especially from mCherry or GFP and they obscure the weaker endogenous protein patterns in the merged panels. This was especially true for overlaps with NR2E3 and PAX6. Single channel images should be

© 2026. Published by The Company of Biologists under the terms of the Creative Commons Attribution License (https://creativecommons.org/licenses/by/4.0/).

provided in all cases where overlaps are being evaluated. Insets as provided in many of the main figures will suffice.

Source of NR2E3 antibody was not provided

First revision

Author response to reviewers' comments

We thank the reviewers for their thoughtful feedback and overall support of our manuscript. Below, we list their comments in black font and our responses in blue font. In some cases, we have split their feedback into subsections to improve clarity.

To address the reviewers' key concern: we spent the post-review period trying to get good sequencing data to elucidate the mutations CRISPR makes in *Otx2* regulatory elements. This proved technically challenging, but we learned that guide-to-guide deletions were rare and that medium-sized indels (6-30bp) were uncommon. Most indels were small (<5bp) and clustered near the guide's preferred cut site. We also found that mutation frequency varied significantly between guides. From our new analysis, we conclude that CRISPR does not generate a sufficient number of enhancer disabling mutations to account for the DHS2 and DHS15 phenotypes seen after three days after electroporation. This argues that CRISPR blocks enhancer activity through Cas9-mediated steric inhibition. Due to the paucity of disabling mutations, the restoration of *Otx2* expression we see over time is likely due to substituting enhancers. Based on these new results and other items addressed below, we have made several edits and additions to the results section. This has lengthened the results section. However, we have also made significant edits to the discussion section to truncate its length and improve clarity. We are grateful for the reviewers' suggestions, which have helped us significantly improve the manuscript.

REVIEWER COMMENTS:

Reviewer 1:

SUMMARY OF THE ADVANCE MADE IN THIS PAPER AND ITS POTENTIAL SIGNIFICANCE TO THE FIELD

The manuscript of Purvis et al. examines the regulation of the key retinal development transcription factor Otx2 through the action of three regulatory elements at the locus. The study investigates an important question in developmental biology, which is how the expression of critical developmental regulator genes is precisely controlled and robust. The use of CRISPRi (CRISPR interference) to target enhancers leads to more penetrant phenotypes which suggests that the use of this technique may be disrupting some fundamental aspect of gene regulation such as enhancer interaction. This is one of the main advances though the mechanism is not clear at this point and this would strengthen the conclusions of the study. There are a number of rigorous approaches and high quality data in this manuscript but there are some key points that should be addressed to make the conclusions more specific and well-supported.

SUGGESTIONS TO AUTHORS

© 2026. Published by The Company of Biologists under the terms of the Creative Commons Attribution License (https://creativecommons.org/licenses/by/4.0/).

Major issues

1. One issue is whether the conclusions are fully supported for some of the data. There are a few examples where the effects are relatively small or not fully quantified where it makes firm conclusions hard to justify. The study does have some good quantitation, but here are other examples that fall into this category.

A) In Fig1, reporters for DHS2 and DHS15 are co-electroporated and it is concluded that the majority of cells use these enhancers simultaneously. However, the individual reporter patterns at E14.5 especially, but also at P0, look qualitatively different from one another (In B, there are a lot of DHS2-active cells near the RGC layer and not nearly as many DHS15-active, for example). This conclusion would be firmer with quantitation and/or with controls of retinas co-electroporated with the same enhancer driving the two fluorophores (DHS2-GFP + DHS2-cherry, for example) to determine what amount of deviation reflects the experimental set-up. In Supp Fig2, the overlap between DHS2 and DHS4 reporters as well as DHS15 and DHS4 reporters looks very concordant. It is not clear how this is squared with what is observed in Fig1 where it would then be expected for DHS2 and DHS15 to be more concordant with each other.

We agree with the reviewer that it was difficult to appreciate how congruent the DHS2 and DHS15 patterns were during development. We have taken their advice and conducted co-electroporation experiments at E14.5 and P0. We co-electroporated DHS2-GFP & DHS2-Cherry, DHS15-GFP & DHS15-Cherry, and DHS2-GFP & DHS15-Cherry constructs. Upon quantification, we observed that Cherry+ electroporated cells tended to be more abundant than GFP+ cells. In matched pairs (*e.g.*, DHS2-GFP vs DHS2-Cherry), double labeled cells never reached 100% overlap, no matter how we quantified the electroporated population's denominator (GFP+, Cherry+, or total electroporated). It is unclear if this represents incomplete co-electroporation, differences in expression kinetics or detectability, or a combination of such factors. Conservatively, we decided to set the matched pair overlap in the total electroporated condition with the highest value (DHS15 at P0) as our maximum sensitivity. Using this, we determined that the DHS2 & DHS15 overlap at both timepoints represents about 75% of this maximum value. We therefore conclude that most cells co-express DHS2 and DHS15 during development. This is now reflected in the revised results section and in a new Fig. S2. Due to creating additional supplemental figures, many of our original supplemental figures have been given new numbering.

B) The study uses Rxrg staining to identify cells in FigS3, but the Rxrg staining is not very localized to the top part of the retina as you would expect, perhaps because the retinas are cultured for 10 days - perhaps a shorter time period would be sufficient to preserve morphology and also see activity? There is not also any quantitation so with the disorganized retina it is hard to determine if there is any specificity for cone cells.

We agree that the cone pattern was especially difficult to appreciate. This turned out to be too difficult to measure in our original experiments because the tissue was disorganized and the RXRG stains were not always reliable. As such, we fully repeated our DHS2 and DHS15 lineage tracing experiments in E14.5 *ROSA-RFP* retinal explants that we collected after 14 days of culture. The more advanced collection point allowed us to use better cone-specific markers- ARR3 and S-opsin. These data are now shown in a revised Fig. S4. Most of the labeled cells were rods, however, we observed a small number of ARR3+ or S-opsin+ cones in both lineages. Moreover, lineage labeled cones in both conditions retained GFP expression, arguing that DHS2 and DHS15 remain active in mature cones, similar to what we observed in rods using *in vivo* P0 lineage tracing. Cones represented about 6% of the GFP+ photoreceptors in both lineages. These new and improved findings are detailed in the revised results section, Fig. S4, and its caption.

© 2026. Published by The Company of Biologists under the terms of the Creative Commons Attribution License (https://creativecommons.org/licenses/by/4.0/).

C) Has a control condition been performed to determine if the Cre vector has any basal activity for Rosa reporter induction when a form without an enhancer is introduced? If this is shown in one of the previous studies that used it, this would be important to reference.

We have done numerous TATA-only GFP reporter experiments in the past (PMID: 25972927, 28829770, 34143204, & 35644251). The TATA only control is quite variable, ranging from zero detectable cells to small, scattered patches of GFP+ cells. When GFP+ cells are detected, they express OTX2 at a low rate after 1-2 days of culture, closely matching the EF1α ubiquitous control overlap with OTX2+ cells. Thus, TATA-only GFP drives a weak ubiquitous pattern. To ensure that this weak background activity is the same in the lineage system, we took the reviewer's advice and electroporated a TATA-only Cre-GFP plasmid into P0 ROSA-RFP mice. Consistent with our prior observations with TATA-only GFP plasmids, we observed very few RFP+ cells in these electroporations. When RFP+ cells were seen, they appeared to randomly label retinal cell types that are born around P0. Moreover, these RFP+ cells were never GFP+ upon collection, suggesting that activity from the TATA promoter is minimal and/or short-lived. Thus, it is unlikely that background activity from the TATA minimal promoter impacts our interpretations throughout the manuscript. Results from this experiment are shown in a revised Fig. S4.

D) Experiments using Crispr guides to target the three different promoters are done and no Otx2 loss-of-function phenotypes are observed. The conclusion is that the promoters are interchangeable, but what is the evidence that the guides are actually effective at targeting the promoters? If they aren't actually effective than that could be the reason for this negative effect? Can an assessment be made that the guides are effective? The same argument could be made for the Otx2 coding region CRISPRi (line 351-) and the SuppFig10 data on the random Otx2 control regions. The authors do note generally that the effectiveness of the CRISPR reagents is a limitation of the study at the end of the paper.

We struggled to measure guide efficiency in electroporated retinal cells. This was due in part to the need for cell sorting, which resulted in some missorted cells along with lysed wildtype cellular DNA contamination in our preparations. This greatly reduced our power to detect mutations. Since our original submission, we have optimized flow sorting conditions to significantly reduce contaminating wildtype cells and their DNA. We also built smaller PCR amplicons and submitted these products for next generation sequencing to estimate mutation types and rates from our cutting CRISPR experiments. The protocols and data from this analysis are included in our revised methods section and the new Table S3. As a benchmark, we examined CRISPR targeting the *Otx2* coding region. We compared the fraction of deleterious mutations in the coding region with the rate of protein loss rate seen through histology. One guide was more efficient than the other in creating deleterious mutations. However, even combined they are not as frequent as the degree of OTX2 loss seen by histology. It is also unclear whether deleterious mutations at each allele correlate in a given cell or if they occur in a truly independent fashion. Regardless, these findings suggest that the mutation rate we detect underreports the efficiency of the CRISPR system in electroporated retinas.

We then examined the mutation spectrum for all of the cutting CRISPR guides we used in the manuscript versus their predicted target regions. This worked for most of the guides, but we struggled to get amplicon sequencing to work for a few- one at DHS15, one at promoter C, and both guides for the promoter A region. This appears to be caused by problems with the amplification step. In general, guide-to-guide deletions were rare. Medium-sized (6-30bp) indels were also uncommon and varied depending on the guide/target region. Instead, we primarily observed small (<5bp) indels and substitutions that occurred close to the predicted cut site (-3 nucleotides from the PAM). For DHS15, we saw little evidence of disabling mutations. For DHS2, we saw a modest number of mutations (~15%) that likely disrupt an essential sequence region (bHLH binding site) of the enhancer. Even with reduced power to detect mutations in our assay, the low number we observed does not explain the OTX2 loss phenotypes we observed. From these observations, we propose in the revised results and discussion sections that enhancer activity is blocked by

© 2026. Published by The Company of Biologists under the terms of the Creative Commons Attribution License (https://creativecommons.org/licenses/by/4.0/).

steric hinderance from Cas9 binding. The subsequent recovery of OTX2 expression, despite the low number of mutations in guide regions, suggests that other enhancers can substitute for their loss-of-function over time.

We also observed a significant mutation burden at promoters B and C (A did not give interpretable results), including some larger deletions. Nonetheless, it is unknown whether these mutations impact essential sequences that disable the *Otx2* promoters. It is also possible that Cas9 sterically inhibits promoter activity. However, it appears that promoters have interchangeability because we observed no major phenotypes from targeting any of them with CRISPR or CRISPRi.

Unfortunately, this deep sequencing methodology is not suitable for measuring the effectiveness of CRISPRi. For this, we plan to do CUT&Tag against activating and repressive histone marks with our various CRISPRi constructs. This is a significant undertaking that will be part of an upcoming manuscript where we explore whether CRISPRi is disrupting interacting enhancers and 3D chromatin architecture.

E) Fig S8 - there is one example of a cell lacking Otx2 that supports the conclusion that a photoreceptor can survive without Otx2- How often is this observed? Could it be in the process of dying? With the use of a cytoplasmic GFP reporter could one determine that it has normal morphology and test for other photoreceptor markers?

This is a great question. We do not observe a large number of these cells (≤5 per section) in *Otx2* coding CRISPR conditions. When seen, however, these cells had NR2E3 expression and normal DAPI nuclear morphology (see the panel included below, the arrow marks a Cherry+, OTX2-negative, NR2E3+ cell). That said, it remains possible that many of these cells progressively die off. A more detailed analysis of this phenotype will be exciting to explore in the future. This will be done best using *Otx2* coding CRISPR systems that are driven by later acting enhancers like DHS2, DHS15, and/or NRL. We are currently optimizing such tools.

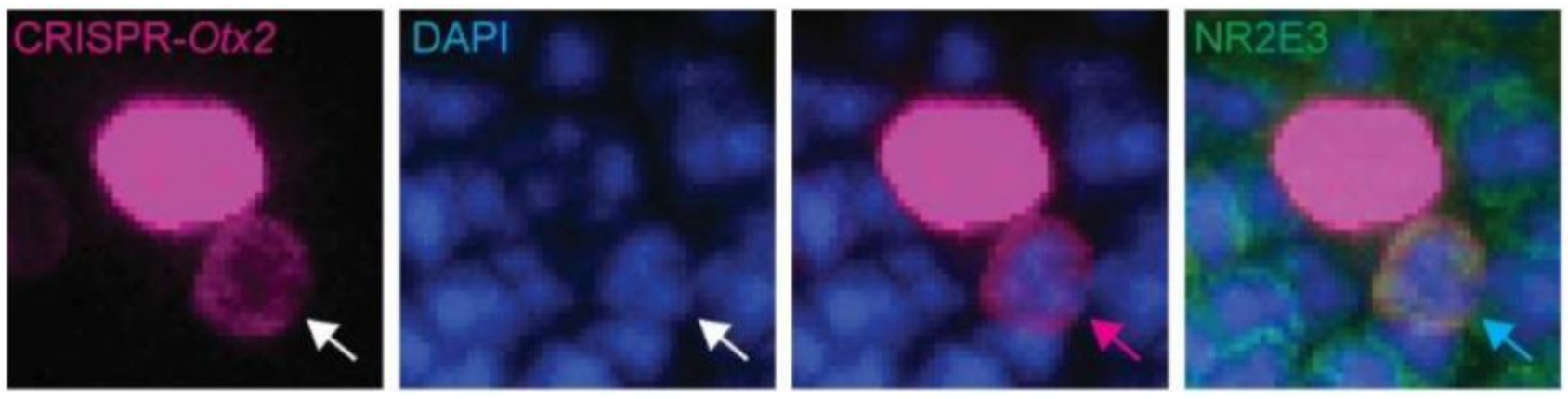

2. The use of DHS2 and DHS15 CRISPR guides to target these elements individually shows relatively moderate effects, but the use of the DHS2 and DHS15 guides together leads to stronger effects which suggests that the elements may be redundant with each other (Line 294-). But since DHS2 and DHS15 are located flanking the Otx2 gene, couldn't it be that the combined use of these guides leads to deletion of the entire Otx2 coding region which is the reason for the phenotype?

We also considered the possibility of large (>100kb) deletions. However, as noted above, our CRISPR mutation analysis shows that “large” guide-to-guide deletions are rare. Most deletions were <30 bp in length. Thus, very large deletions are likely extremely rare and would not contribute meaningfully to the phenotype. We also tested a CRISPR guide pair that flanked the first and last promoter regions, but this did not result in any change in OTX2 expression. While not measured directly, this further argues that multi-kilobase deletions are rare in retinal cells electroporated with CRISPR constructs.

© 2026. Published by The Company of Biologists under the terms of the Creative Commons Attribution License (https://creativecommons.org/licenses/by/4.0/).

3. The interpretation of the CRISPRi results is an important part of the paper as they are interpreted to show that the enhancer elements are interacting with each other. One instance where this is raised is with regards to DHS4 CRISPRi line 342-. "This perturbation acted like a dominant negative" - not sure what is meant by this? It seems likely that the authors are making an analogy to the genetic definition of a dominant negative allele whereby the mutant copy also is able to decrease the function of the product of the wild-type allele. Not sure if that analogy holds here in that the CRISPRi is likely targeting both alleles already and the extra effect may be on some other distinct genetic component, like another enhancer. Clarification on what is meant by dominant negative and discussion of how this particular term is applicable to this situation would be helpful.

Yes, we refer to “dominant negative” meaning that the DHS4 CRISPRi can block the activity of other contributors to *Otx2* gene regulation, like enhancers, that were not directly targeted. We agree that this terminology could be confusing and have removed it from the manuscript.

It is also suggested that the CRISPRi was much stronger than conventional CRISPR. The suggestion on line 348 is that this is consistent with the CRISPRi affecting more than just its targeted enhancer. But why couldn't it be that the CRISPRi is just more effective at silencing the enhancer?

It is certainly possible that CRISPRi is more efficacious at silencing enhancers than disabling mutations or steric hinderance caused by conventional CRISPR. However, we observed that acute OTX2 suppression by CRISPR and CRISPRi (after 3 days) using the same guides against DHS2 and DHS15 was the same. This suggests that both systems block enhancer activity equally. In the long run, the stronger CRISPRi phenotype could be due to its ability to more efficaciously block enhancer activity. However, due to the low number of guide region disrupting mutations in our conventional CRISPR experiment, it seems likely that Cas9 would have considerable time to sterically hinder enhancer activity. Thus, it is likely that the difference in long-term phenotypes is due to the CRISPRi system’s epigenetic modifiers acting beyond the targeted enhancer to disrupt substituting elements or 3D genomic structure. We are planning experiments to measure how CRISPRi impacts histone modifications at targeted and non-targeted enhancers to determine whether it disrupts interacting *cis*-regulatory elements. We are also planning chromatin conformation experiments to look at 3D architecture impacts from CRISPR vs. CRISPRi.

The discussion has several good points about why CRISPRi may be more efficacious than conventional CRISPR and how this may be due to interactions with other enhancer elements at the locus. However, there isn't a mechanism identified and so there is not a clear conclusion that can be drawn about what these results mean in terms of normal gene regulation.

We agree with the reviewer that the mechanism of CRISPRi is not fully elucidated by our data. However, we believe that it is appropriate and important to briefly speculate about potential gene regulatory mechanisms, which we are excited to test going forward.

4. The two enhancers of focus (DHS2 and DHS15) are active in similar cell populations but suggested to have different upstream regulation (at least to some extent) because the sequence motifs that are present in critical regions are different. The motifs correspond to predicted regulation by certain transcription factors, but there is not follow-up to show that modulation of these transcription factors (CRISPRing them or misexpressing them, for example) have differential effects on the two elements. That would make for a more solid case that these elements are in fact regulated differently.

© 2026. Published by The Company of Biologists under the terms of the Creative Commons Attribution License (https://creativecommons.org/licenses/by/4.0/).

This is a great future direction that we are planning to pursue. We have included a new Fig S16 that shows NRL CUT&Tag data from several postnatal timepoints (see also reviewer 2, points 16 & 22, below). These data show a progressive increase in NRL binding to the DHS15 minimal sequence as the retina matures, but none is seen in the DHS2 region. This further argues that regulation of these enhancers is distinct. Furthermore, we see DHS15 activity in cones, which do not make NRL. Hence, these enhancers may also be regulated differentially between OTX2+ cell types.

Minor

Line 251,2 - "positive control plasmids targeting the Otx2 coding region remain highly efficacious at P7" - it is not clear what this means.

Our positive control CRISPR plasmid targets the *Otx2* coding region. At P7, we still see a drastic decrease in OTX2 expression in cells electroporated with this plasmid. Indeed, the efficacy of this positive control CRISPR plasmid against the *Otx2* coding region does not diminish with age in any of our experiments.

In mutants for Fig 2 and S4 it is not clear if there is a change in specificity if the amount of expression is very low. Then perhaps most of the GFP expression is from the basal activity of the vector and that is not likely to be Otx2-enriched and so it looks like it is not specific? It would be helpful if this interplay of specificity and activity was discussed for low activity situations.

The reviewer is shrewd to point out that the minimal TATA promoter may weakly drive a non-OTX2 enriched pattern (see point 1C, above), which could dilute enhancer specificity measurements. However, any mutation that strongly reduces activity still highlights important regulatory sequences in the enhancer, even if this does not impact specificity. It is also unclear how much activity is supplied by the minimal promoter, though our lineage data argues that it is modest and short-lived (see point 1C, above). The wide range of activities (from nearly zero to full) in our mutants also suggests that the minimal promoter is not a major driver of activity.

Line 146 - suggest that the number of bipolar cells was variable and maybe because the elements mark only a subset of the bipolar population - not sure that is a valid argument as the bipolar subtype percentages are pretty consistent across biological replicates?

We agree. We proposed this argument and then a few lines afterwards argue against it for the reasons the reviewer points out. We have condensed this part of the results section to improve clarity.

Reviewer 2:

SUMMARY OF THE ADVANCE MADE IN THIS PAPER AND ITS POTENTIAL SIGNIFICANCE TO THE FIELD

OTX2 plays a crucial role in specifying retinal cell fates, influencing the formation of about 85% of retinal cells. Its loss results in the absence of photoreceptors and bipolar cells and an increase in other cell types. Otx2 is activated in progenitor cells just before they exit the cell cycle and remains expressed in mature photoreceptors and bipolar cells, but not in amacrine or horizontal cells. Of the three enhancers (DHS2, DHS4, DHS15) identified previously, DHS4 is shown to initiate Otx2 expression. In this manuscript, the authors examined the function of DHS2 and DHS15 by matched CRISPR/Cas9 and CRISPRi targeting. The authors concluded that multiple cis-regulatory elements interact and substitute for each other to ensure robust OTX2 expression to specify retinal cell fates.

© 2026. Published by The Company of Biologists under the terms of the Creative Commons Attribution License (https://creativecommons.org/licenses/by/4.0/).

Overall, this is an excellent study that complements previous research and offers new insights into Otx2 expression during retinal development. The data being presented is definitely significant. The potential underlying mechanisms for the regulation have been discussed and limitations of the study acknowledged. However, the data does not completely justify the conclusions. Additionally, the experimental design is rather linear and alternate explanations exist. Otx2 gene likely has multiple cis-regulatory elements (CREs) given its key role in determination of multiple retinal cell fate. Could it serve as a competence factor for different fates but may not determine any on its own? HiC data at high resolution may be necessary to dissect Otx2 regulation in distinct spatiotemporal contexts. If mouse data is not available, can the authors get some insights from human HiC data? The authors are generally careful but have not discussed additional interpretations and experiments that could test. Some figures lack clarity as well. Discussion is long and redundant. The authors should primarily discuss key findings, integrating these with what is published. There is no reason to restate the results.

SPECIFIC COMMENTS AND SUGGESTIONS:

1. Summary statement, Abstract and text: "cis-regulatory robustness" - unclear what this really means? Is it "precision"? Also, is flexibility same as redundancy or are they talking about complementarity or synergy?

> We use “robustness” to reflect the quality of being unlikely to break or fail under stress. Flexibility and enhancer redundancy are similar concepts. Either way, this means that a cell has multiple gene regulatory options to get to a common outcome. This prevents target gene regulatory failures due to genetic or environmental stressors, thereby canalizing development. Canalization of development is also referred to as “robustness”. Here, we use *cis*-regulatory robustness to encompass flexibility, redundancy, and substitution at the level of gene expression.

2. Abstract and text: "Each enhancer is needed for normal Otx2 expression." Quantitative statements are difficult based on immunostaining and fluorescence methods. Additionally, recent studies have shown that the concentration of a transcription factor is critical in specific contexts. Same factor can have distinct functions, interaction with other proteins, or binding rate/affinity when present in different concentrations.

> Our CRISPR mutagenesis experiments show that DHS2 and DHS15 are required for OTX2 expression after three days. However, we did not measure expression levels with our manipulations, so it is possible that they also have a quantitative effect on *Otx2* transcript abundance during development and into adulthood. We refer to expression in the manuscript as the detectability of OTX2 protein (binary), rather than absolute (continuous) levels. Future work is needed to look at quantitative effects of our enhancer perturbations and how upstream regulators bind the sequences to regulate cell fate.

3. It is difficult to assume that Otx2 must be expressed at same levels in all cells and at all times, as implied by the last sentence in Abstract.

> We apologize, but that was not the intention of the last sentence. Rather, we wanted to convey that robustness (see reviewer 2, point 1, above) in the system enables *Otx2* to be expressed in its normal spatial and temporal developmental patterns. Future work will be needed to look at the quantitative expression aspect of each enhancer.

© 2026. Published by The Company of Biologists under the terms of the Creative Commons Attribution License (https://creativecommons.org/licenses/by/4.0/).

4. It's unclear how the authors evaluated the efficiency of the guide RNAs used in the study.

> We have now conducted an analysis of CRISPR mutagenesis efficiency in electroporated retinal cells. Please see the introduction of this document and the comments to reviewer 1 (point 1D, above) for details. These data are now shown in a new Table S3 and in substantial revisions to the results, discussion, and methods sections.

5. The authors should incorporate the cell type specificity of Otx2 enhancers (DHS2 and DHS15) from published single-cell ATAC-seq data (PMID: 34788628). Please incorporate any Hi-C and K27Ac data if available since these data would be critical for obtaining a more comprehensive overview of cis-regulatory elements that dictate Otx2 expression.

> We have looked at single cell ATAC-seq data in the past to try to differentiate accessible regions in specific cell identities. This was not especially informative for DHS2, 4, or 15, as they fit the expected patterns. Of note, human single cell ATAC-seq in retinal organoids shows that DHS2, 4, and 15 have largely conserved accessibility patterns (PMID: 35081356; Figure 3). We and others (PMID: 32631829) have examined a very large number of accessible sites around the *Otx2* locus. However, the vast majority of these sequences lack enhancer activity in our hands. Due to the low success rate of ATAC-seq and histone modifications to predict retinal enhancers, we favor taking a focused chromosome conformation capture (4C) approach with defined OTX2+ cell sets to define promoter-enhancer and enhancer-enhancer contacts in the future. We have also looked at published mouse retinal Hi-C datasets. However, these do not have sufficient resolution to identify small enhancer regions or how they may be interacting with each other. We believe that taking a 4C approach will be superior for identifying novel enhancers and how they interact dynamically across retinal development. It will also allow us to manipulate the system with CRISPRi and study how it changes 3D chromatin interactions and architecture.

6. Throughout the manuscript, cell fate change was evaluated by CRISPR targeting from P0 onward. However, the fate of all cones and 10% of rods is already specified before that and would likely escape CRISPR engineering. Without additional studies at early time points, can the authors draw broad conclusions? This limitation of the experimental design should be included in Discussion. How did the authors identify photoreceptor vs bipolar cells? What cell markers were used?

> The reviewer is correct that we did not comprehensively search for all cell fate effects from our CRISPR manipulations. Instead, we focused on the postnatal retina, where we could perform *in vivo* manipulations and examine intact tissue. This timing makes it easier to see changes in fate outcomes between postnatally generated OTX2+ (rods, bipolars) and OTX2-negative (amacrines, glia) cell types. Starting around P7, OTX2 expression becomes modest in photoreceptors and remains intense in bipolar cells. Coupled with cell localization differences, this makes discriminating between rods and bipolars cells straightforward with OTX2 stains.

7. Based on plasmid-based reporter assay in explant cultures, both DHS2 and DHS15 have enhancer activity at embryonic day embryonic day (E) 14.5 and postnatal day (P) 0. However, in-vivo assay on DHS2 and DHS15 from previous study (Wilken et al., 2015) suggest low expression of both the enhancers at P0 and increasing later at P7. Therefore, based on the previous report the DHS2/DHS15 enhancers should have no or limited expression at embryonic day. Please clarify.

> This is a great observation. Yes, the expression pattern in the Wilken et al, 2015 paper showed modest DHS2 and DHS15 expression around P1, leading us to expect that these enhancers would have little if any activity during embryonic development. However, when

© 2026. Published by The Company of Biologists under the terms of the Creative Commons Attribution License (https://creativecommons.org/licenses/by/4.0/).

we revisited this idea through numerous electroporations in both embryonic and postnatal retinas, we found that DHS2 and DHS15 reproducibly had more activity than initially appreciated. The discrepancy is likely from the lack of embryonic data, some technical variance, and the modest sample size used in the original 2015 paper. However, it is notable that the DHS2 and DHS15 P7 data closely match our current results. The accessibility of these enhancers (PMID: 35081356; Figure 3) in immature developing human retinal organoids is also consistent with early ("embryonic") activity.

8. In Fig 1B-C, many OTX2+ cells do not appear to express either plasmid, and many cells expressing DHS2 or DHS15 don't express OTX2. This can be better depicted and explained. Additionally, the expression of both individual and co-expressed reporter plasmids for DHS2 and DHS15 should have a separate figure.

We agree that this can be better quantified. As described above (reviewer 1, point 1A), we now quantify the overlap between these two enhancers in a new Fig S2 and estimate that they are co-expressed about 75% of the time.

9. Similarly, in Fig E-F, many OTX2+ photoreceptors and bipolars are not labeled with Rosa-RFP and DHS2 or DHS15. How is OTX2 expression maintained without DHS2 or DHS15 activities?

This reflects a technical limitation to electroporation assays. The key limitations are that: (1) only a subset of cells was electroporated, (2) and just a subset of them localized plasmids into the nucleus where they can be expressed. As a result, relatively few retinal progenitors and their daughters express plasmid constructs. Thus, we expect to label only a subset of the OTX2+ cell population. Overcoming this mosaicism requires a uniform labeling system, which could be addressed through the creation of transgenic mice in the future. In terms of OTX2 expression, it is likely that other enhancers substitute for the loss of DHS2 and DHS15 activities. Other enhancers are needed in mature bipolar cells, which do not use DHS2 or DHS15 to sustain *Otx2* expression in the adult retina.

10. The authors hypothesize "DHS4+ cells would transition towards using DHS2 and DHS15 to maintain OTX2 expression". Based on the results DHS2 and DHS15 are simultaneously active at E14.4 and P0. It does not provide convincing evidence for transition of DHS4+ cells. Perhaps other enhancers are involved. Additionally, the authors should incorporate published data (PMID: 31493975, PMID: 28472656) to map poised active status of the DHS2 and DHS15 in developing mouse retina.

Our data show that this transitional relationship is likely. We previously showed that DHS4 expression is transient and upstream of both OTX2+ and OTX2-negative cell identities (PMID: 34143204). These DHS4+ cells are in transition from progenitors to neurons. In contrast, our DHS2 and DHS15 lineages are activated early and restricted to photoreceptors and bipolars, which maintain OTX2 expression. We show in Fig. S3 that DHS4 reporters can overlap with DHS2 or DHS15 reporters. Due to the short-term stability of the fluorescent reporters and the known lineages, the most parsimonious explanation is that cells turn on DHS4 before activating DHS2 and/or DHS15. Moving forward, we plan to map the activity of these enhancers relative to each other using reporter co-electroporation, single cell RNA-sequencing, and pseudotime analysis (similar to PMID: 34143204). From this, we will have sufficient temporal resolution to map the spatial and temporal patterns DHS2/4/15 activity patterns relative to each other. This will improve the usefulness of these enhancers as precision gene manipulation tools for future work. As described in the discussion, we predict that other enhancers play a role in *Otx2* activation or maintenance.

© 2026. Published by The Company of Biologists under the terms of the Creative Commons Attribution License (https://creativecommons.org/licenses/by/4.0/).

11. Otx2 is expressed even in mature retina though it's expression pattern changes. Can the authors perform reporter assays similar to figure 1 for DHS2 and DHS15 in adult mouse retina? The activity of most cell-type specific enhancers is spatiotemporally controlled.

Yes, we have done this by examining the expression of our reporter constructs in newborn mice after 21 days. In Figs. 1E’ & 1F’, we show that DHS2 and DHS15 are active (GFP+) in rods, but not bipolar cells, within the P21 mouse retina. As described above (reviewer 1, point 1B), we repeated embryonic lineage tracing with DHS2 and DHS15 constructs specifically to examine cones. DHS2 and DHS15 activities were maintained in cones at the end of the culture period (~P9). From this, we conclude that DHS2 and DHS15 activity is maintained in mature rods and cones. The use of viral vectors or transgenic mice could be used in the future to test whether these enhancers remain active at even older timepoints.

12. In Fig S3, why did the authors specifically focus on cone photoreceptors?

DHS2 and DHS15 lineage tracing in P0 retinas shows labeling of rods and bipolar cells. However, the remaining OTX2+ cell type, the cone, is generated before birth in mice. Since electroporations rarely deliver DNA into the nucleus of postmitotic cells, we tested our lineage constructs at an embryonic time when cones are being generated from retinal progenitors. As mentioned above (reviewer 1, point 1B & reviewer 2, point 11), we have repeated this embryonic lineage tracing and show more convincingly that cones are in the DHS2 and DHS15 lineages. Further, we see that cones maintain DHS2 and DHS15 activity as they mature, similar to rods.

13. In Fig 3, which panels are showing single electroporation of DHS2 or DHS15, or co-electroporation of both plasmids? Looks like panel D and H are showing co-electroporation but many cells are expressing only mCherry or GFP but not both? Did the authors consider these cells as single deletion of DHS2 or DHS15? The authors can use single electroporated samples.

For Fig. 3, we electroporated single targeting plasmids and also co-electroporated plasmids to target both DHS2 and DHS15. The negative control included non-targeting guides and either GFP or Cherry to match the co-electroporated DHS2 and DSH15 condition. Due to space constraints, we only showed the double electroporated conditions along with the positive control targeting the *Otx2* coding region. Nonetheless, all of the quantified data are shown in the graphs. For transparency, we include images of DHS15 and DHS2 single targeting below at both E14.5 and P0.

© 2026. Published by The Company of Biologists under the terms of the Creative Commons Attribution License (https://creativecommons.org/licenses/by/4.0/).

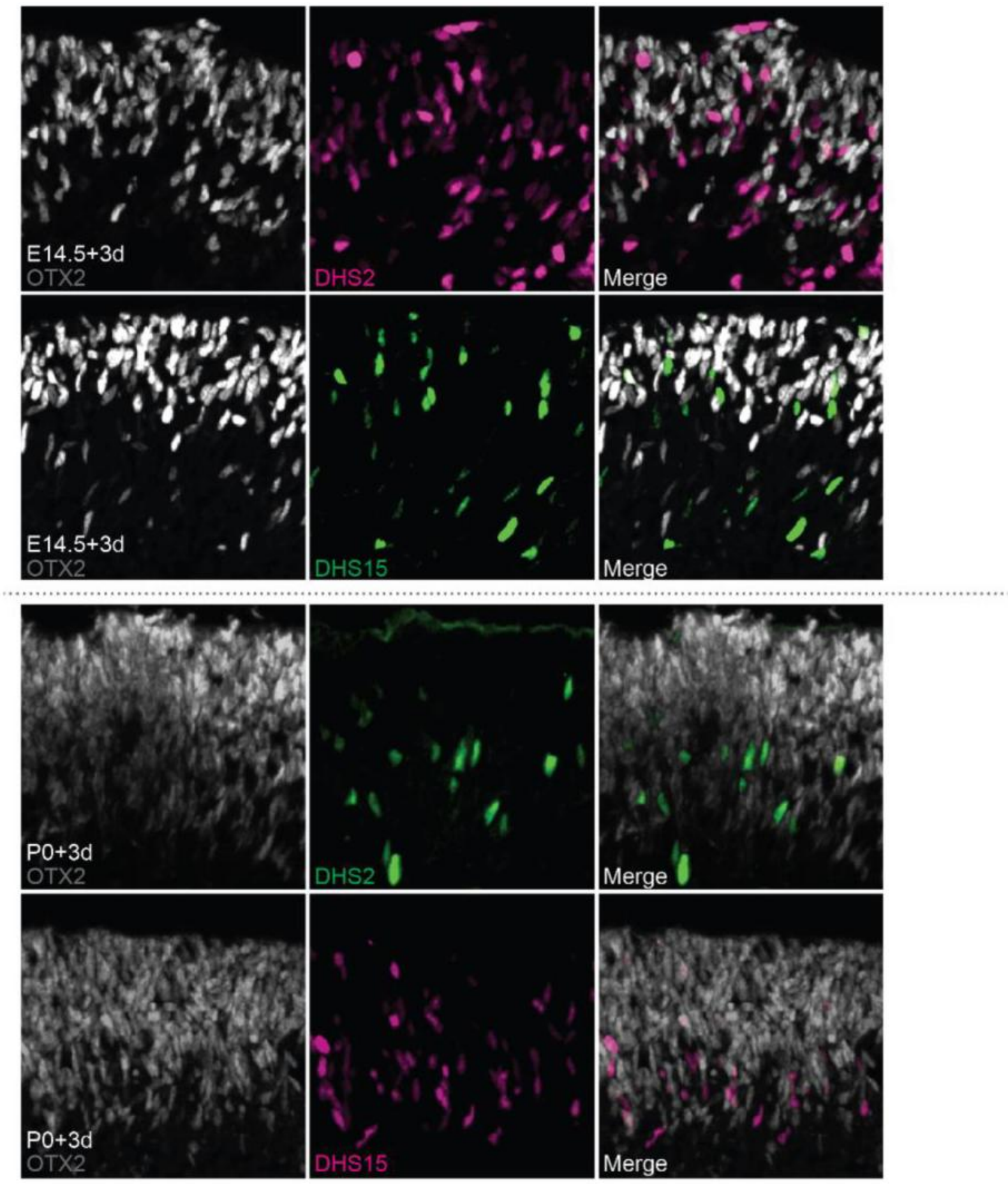

14. In Fig 4, immunostaining images for DHS15 mutation at 7d were not included. Similarly, images for single mutation of DHS2 or DHS15 at 21d were not included. Fig 4B and Supp Fig 9B should include the design of a single plasmid expressing all 4 gRNAs.

As mentioned above, we did not include images for every condition due to limited figure space. However, all quantified data is shown in the graphs. We have revised Fig. 4B and Fig. S8 to include a graphic showing the 4-guide plasmid design. For transparency, we include images of DHS15 at P7 and DHS2 & 15 at P21 below.

© 2026. Published by The Company of Biologists under the terms of the Creative Commons Attribution License (https://creativecommons.org/licenses/by/4.0/).

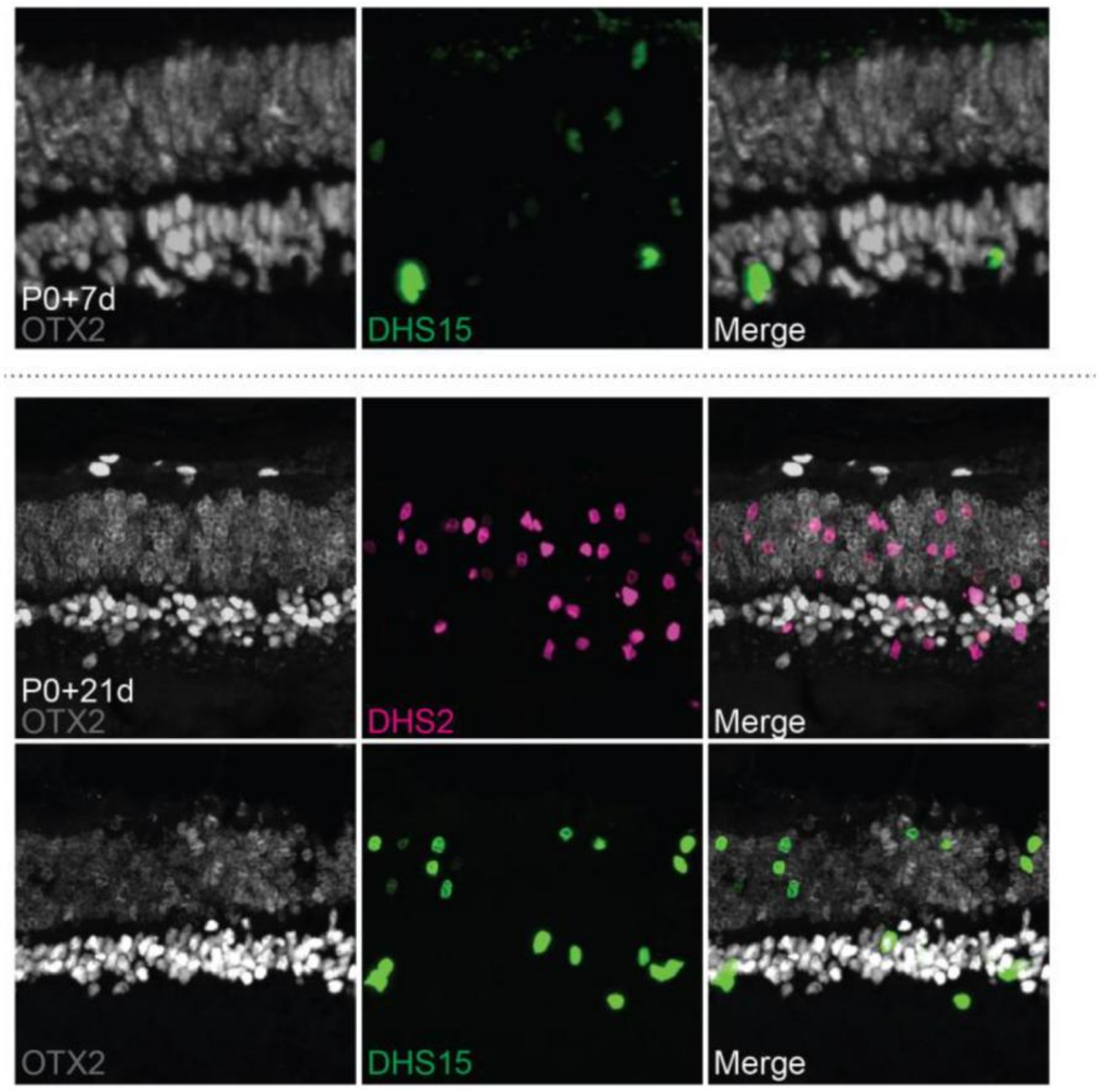

15. Did the authors think different cell types may response to the loss of DHS2 or DHS15 differently? In Fig 4F and J, the authors may quantify OTX2 expression in photoreceptor and bipolar cells separately.

> Yes, we also thought that this could occur! This is why we measured the rod-to-bipolar ratio in our CRISPR manipulations (Fig. 4K). However, this showed that while the number of OTX2+ cells was reduced in the DHS2/15 double targeting conditions, it did not preferentially impact rods vs. bipolars. The ratio was equivalent across negative control and enhancer targeting conditions.

16. Authors have used JASPER to predict transcription factor binding for essential regions of DHS2 (Fig. S5) and DHS15 (Fig. S6). Published results of mapping retina specific TFs using ChIP-seq/CUT&RUN on mouse retina should be incorporated to validate the differential binding of retina specific TFs on DHS2 and DHS15.

> We showed OTX2 binding datasets in our original submission. We now include postnatal CUT&Tag datasets for retinal NRL binding (see also reviewer 1, point 4, above) in a new Fig. S16. These data show that NRL binds to DHS15, but not to DHS2 or DHS4. This binding was not appreciable until rods started to mature, suggesting that DHS15 is regulated differently over time and between rods and cones. We have updated our model figure to reflect NRL binding only to the DHS15 enhancer (Fig. 7B). In the future, we plan to explore upstream regulator binding to each enhancer in detail.

17. "Targeting DHS2 and DHS15 significantly reduced OTX2 overlap (~15%), but this effect was modest compared to P0 retinas examined after 3 days (~50%) and the positive control condition (~75%)". Did the authors profiled for other cell types which might be responsible for modest phenotype at P7?

© 2026. Published by The Company of Biologists under the terms of the Creative Commons Attribution License (https://creativecommons.org/licenses/by/4.0/).

We did not do a comprehensive screen for conversion to OTX2-negative cell types at P7. This is a major goal of our future studies to understand how enhancer perturbed cells behave after temporarily losing OTX2 and subsequently reacquiring expression.

18. Line 296, the authors stated that "our results suggest that DHS2 and DHS15 partially substitute for each other to enable OTX2 expression and photoreceptor and bipolar cell formation". While panel 4K shows the ratio of photoreceptor vs bipolar is not changed, how about the absolute number of photoreceptor cell and bipolar? Did the authors suggest a cell fate shift from photoreceptor/bipolar to amacrine cell?

Yes, the absolute number of OTX2+ cells goes down modestly in DHS2/15 double CRISPR targeting conditions (Fig. 4J). In Fig. S10, we show that bright round PAX6+ cells increase in the DHS2/15 double targeting condition compared to control. This is consistent with a fate shift to amacrine cells.

19. Fig 5 should include CRISPRi images against DHS15.

As described above, we omitted some images for the sake of space. The quantification of the data is present in the graph of figure 5. For transparency, we include images of DHS15 CRISPRi at E14.5 and P0, below.

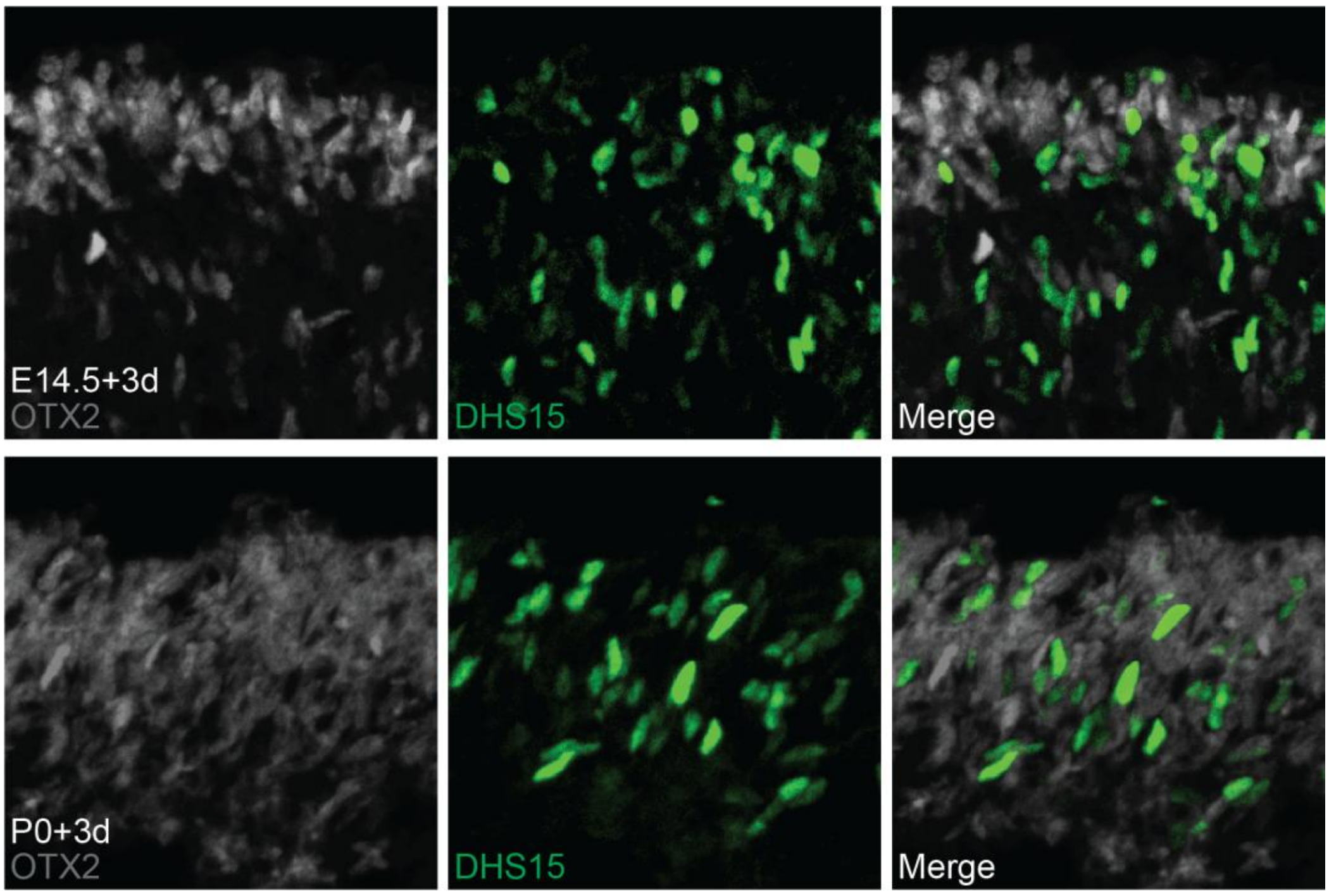

20. In Fig 6, the authors should explain why CRISPRi targeting of DHS2, DHS4, and DHS15 shifted the distribution of electroporated cells towards the inner nuclear layer and again should use cell type markers to evaluate photoreceptor/bipolar ratio. Was the unchanged ratio due to the inefficiency in targeting? In Supp Fig 10, the authors should show the images of g3 at E14.5 since it led to increased OTX2 expression.

Prior work has shown that *Otx2* loss results in the increase of amacrines and ganglion cells. Since we targeted P0 retinas, the expected result from CRISPRi would be to lower OTX2 expression and shift cells towards an amacrine fate (RGCs are not made postnatally). This fate change explains the prominent shift of electroporated cells into the inner INL, where

© 2026. Published by The Company of Biologists under the terms of the Creative Commons Attribution License (https://creativecommons.org/licenses/by/4.0/).

amacrines normally reside. Note also that the reporter is brighter in amacrine cells, making this shift especially prominent to the eye. The lack of change in the photoreceptor-to-bipolar ratio suggests that photoreceptor and bipolar cell fates are equally impacted. We have revised Fig. S11 to include an image of the g3 targeting.

21. Fig S14 can be moved to Results section rather than Discussion.

While we would very much like to include more display items in the main figures, there is not enough space.

22. Line 453: the authors state that "we did not observe NRL ChIP signal at this enhancer (Hao et al, 2012)". NRL ChIP-seq was performed using adult retinas. The authors should check for NRL Cut&Run data at Otx2 during retinal development (PMID: 35776116)

Thank you for this suggestion! Using this CUT&Tag dataset, we were able to show that NRL can bind to the DHS15 minimal enhancer sequence in the postnatal retina. This is included in a new Fig. S16. We have also edited the discussion section to reflect this new knowledge. Please also see the above comments (reviewer 1, point 4 & reviewer 2, point 16).

Reviewer 3:

SUMMARY OF THE ADVANCE MADE IN THIS PAPER AND ITS POTENTIAL SIGNIFICANCE TO THE FIELD

Previous studies from the senior author and others have identified enhancers that control temporal and cell-state dependent elements of Otx2 expression. Otx2 is a critical transcriptional regulator of retinal development, and the spatio-temporal regulation of its expression is complex and not fully understood. In this study, Purvis and coauthors perform a detailed assessment of enhancer properties in vivo and ex vivo contexts using electroporation of developing mouse retina. The study incorporates carefully designed enhancer testing to identify required minimal sequences. Mining of published ATAC-seq and Chip-seq datasets enabled predictions of candidate interacting transcription factors although they were not the primary focus. Rather, the team used CRISPR/Cas9 mutation and CRISPRi testing to test the candidate regulatory sequences within the enhancers and to identify potential interactions between them. Three enhancers were the primary focus, DHS4, which activates Otx2 expression in retinal progenitors and DHS-2 and -15 important for maintained expression in differentiating and mature photoreceptors and bipolar cells. By using both CRISPR and CRISPRi, the authors show that DHS-2 and -15 have overlapping and unique characteristics. Interestingly, CRISPRi targeting of the key enhancer sequences had stronger effects than CRISPR targeting at the same locations. CRISPRi testing of sequences near, but not part of the enhancers had minimal effects, which demonstrated specificity of these effects. Through iterative testing of these enhancers in isolation and in combination, their requirements to regulate Otx2 were shown to not be absolute but still important for the complex spatio-temporal regulation of Otx2 in the retina. While some effects on cell fate choice were observed with CRISPR targeting, the effects were more pronounced with CRISPRi. How this occurs is not clear, but the effects were convincing and reveal a robustness of the Otx2 enhancer environment to regulate Otx2. It is my opinion that this is a significant contribution to understanding how transcription factor expression is regulated in complex developmental programs such as that in the retina.

SUGGESTIONS TO AUTHORS

The manuscript was well organized, experiments were well-controlled, and sample sizes appeared to be sufficient and analyzed with appropriate statistics. The literature was well cited. One issue was that the results and discussion were repetitive in several areas and editing would improve the discussion's readability.

© 2026. Published by The Company of Biologists under the terms of the Creative Commons Attribution License (https://creativecommons.org/licenses/by/4.0/).

Thank you. We have made several edits throughout the results and discussion to simplify the text and improve readability. We have reduced instances where we were reviewing the results to both shorten and streamline the manuscript.

The authors make the argument that the CRISPRi is having more potent effects than CRISPR/Cas9 targeted sequences because steric hindrances are interfering with the flexibility and interactions of the enhancers. This explanation is hard to follow. Is the point that the CRISPRi protein complex is causing the steric hindrances between the interacting enhancers? I could imagine two other possibilities: that bringing a transcriptional repressor to an enhancer will have a dominant negative effect as proposed for DHS4, or that binding of the CRISPRi complex is displacing more transcription factors at the enhancer than the CRISPR/Cas9 mutant.

Steric hinderance is one possible way that CRISPRi could block enhancer activity. As the reviewer mentions, this could happen if the dCas9 complex parked on the enhancer physically blocks its ability to bind transcription factors. Indeed, this can happen with regular Cas9 when binding is protracted, as described in our revised discussion section. Since CRISPRi fuses epigenetic modifiers with dCas9, it may also inhibit other enhancers in its local 3D environment, creating a "dominant negative" effect. That enhancer CRISPR can be overcome while CRISPRi cannot suggests that CRISPRi acts beyond just the targeted enhancer. The reviewers' third model is also possible (see also reviewer 1, point 3), as epigenetic modifications may block enhancer activity better than mutations. However, we see essentially equivalent results from CRISPR and CRISPRi after 3 days of development, suggesting that they have similar efficacy. We have revised the discussion extensively to clarify our logic. In the future, we plan to examine how these enhancers interact in 3D and whether this and their epigenetic states are disrupted by CRISPRi.

There are several figures, especially in the supplement that have saturated signal, especially from mCherry or GFP and they obscure the weaker endogenous protein patterns in the merged panels. This was especially true for overlaps with NR2E3 and PAX6. Single channel images should be provided in all cases where overlaps are being evaluated. Insets as provided in many of the main figures will suffice.

The reason for saturation was that the electroporated photoreceptors tended to be labeled less intensely than other cell types (*e.g.*, amacrines), making it difficult to get well-balanced images. To offset this issue, we have included single channel and merged insets in revised Figs. 4, S4, S9, and S10.

Source of NR2E3 antibody was not provided

Thank you for pointing out this omission. We have added the NR2E3 antibody information into a revised Table S2 spreadsheet, in the "Antibodies" tab.

<u>Second decision letter</u>

MS ID#: dev.204881R1

© 2026. Published by The Company of Biologists under the terms of the Creative Commons Attribution License (https://creativecommons.org/licenses/by/4.0/).

MS TITLE: A robust cis-regulatory network ensures Otx2 expression during retinal development

AUTHORS: Ian Purvis, Omar Ochoa Olmos, Ko Park, Michael Kaufman, Charles Henry, Christopher Schaaf, Olivia Clise, Corey Tesdahl, Ami Haas and Joseph A. Brzezinski IV

Dear Dr Brzezinski IV,

I have now received the reports of two of the referees who reviewed the earlier version of your manuscript and I have reached a decision. The reports are appended below and you can access them online:

The overall evaluation is positive and we would like to publish a revised manuscript in Development, provided that you satisfactorily address the remaining suggestions and comments of referee 1. Please attend to all of the reviewer's comments in your revised manuscript and detail them in your point-by-point response. If you do not agree with any of their criticisms or suggestions explain clearly why this is so.

Reviewer 1

Advance summary and potential significance to field

Comments for the author

Overall, the authors have addressed a number of the issues raised in the initial review. A concern remains with regard to the effectiveness of the CRISPR reagents and the interpretation of the data.

The authors have included CRISPR mutagenesis sequencing data which is a good addition to the manuscript. However, a stronger case needs to be made or expanded for how this supports some of their conclusions. In addition, some further annotation/clarification of TableS3 would be helpful. This second issue is addressed first, as it may be relevant to the misunderstanding of the reviewer and the points made subsequently. Placing the location of the primers used to generate amplicons on the BAM file visualization would be helpful for cases like DHS15 where it is not clear why the control and the DHS15 amplicons are different sizes - is that because there were different amplicons between the control and the experimental? If so, that is fine as they capture the DHS15-g2 region, but it would help clarify why these plots look different. Another issue is that on the left side of each sheet, one guide sequence and the amplicon primers are listed. But for the conditions besides "Otx2", the constructs are indicated by the main text and the BAM stack on the right to contain two guides that target the region. Thus, it is not clear what the single "guide sequence" represents. Is it just one of the two guides or was there only one guide assessed in the experiment - the schematic would suggest not, but it is also striking that for DHS2 and Exon1B there is no evidence that one of the two guides are working (for DHS15, one guide region wasn't assessed and Exon1C does show evidence of both working).

While the inclusion of this data do add to the manuscript, it is not clear that it supports some of the conclusions. One issue brought up in the first review is the possibility that combined DHS2 and DHS15 CRISPR targeting leads to a stronger Otx2 phenotype because it deletes the Otx2 gene which is flanked by these two regions. This is an alternative explanation to the interpretation that these two regions serve a redundant function in cis-regulation of Otx2. In the TableS3 data for DHS2, there does not seem to be any evidence that g1 is working at all and so the extent with the g1/g2

© 2026. Published by The Company of Biologists under the terms of the Creative Commons Attribution License (https://creativecommons.org/licenses/by/4.0/).

combination to which the DHS2 element is disrupted beyond the immediate vicinity of g2 seems minimal. For DHS15, data was not obtained for the g1 location and g2 did not seem to have an effect. So this also does not present a clear picture that the DHS15 region is being disrupted. This raises the possibility that targeting DHS2 and DHS15 alone with guide pairs is not effective because at most only one guide is working and only causing very local changes. But in combination, DHS2 and DHS15 could lead to a large gene deletion between each of their one active guides. In the author's rebuttal it is stated that "In general, guide-to-guide deletions were rare" and from the revised manuscript text Line 305 "Importantly, we found little evidence of deletions spanning guide pairs." However, from TableS3, the only combo guide pair with evidence that both guides were working (and two active guides would be important for seeing deletions that would be predicted from a simultaneous cut at two sites and a subsequent NHEJ reaction that deletes the intervening sequence) was for Exon1C and the plots would suggest that this combination does indeed have a much higher rate of deletions aligned with each of the guide cut sites. It is not clear how these BAM plots should be read to say there is a precise guide to guide deletion (and further clarification of this in the text would be helpful), but more than half of the reads have a sharp cutoff at one of the two guide sites on the side closest to the other guide suggesting a deletion oriented towards the other guide. Sequence data from each of the individual guides alone would inform whether the interpretation that the combined sequence data supports a deletion effect. Thus, strong evidence to rule out a DHS2 to DHS15 deletion and removal of the Otx2 coding region is not present in this sequencing data. Thus, this possibility should be noted or evidence to argue against it included. The conclusion that Cas9 is binding to these regions and sterically inhibiting enhancer regulation could have much stronger support by using the DHS2 and DHS15 guides with a simple dead Cas9 version without any interference protein domains. That would also rule out that there is a large deletion being created with DHS2/15 simultaneous CRISPR.

For the investigation of the promoter usage of Otx2, the lack of sequencing data for exon 1A and a lack of efficacy of the guides targeting exon 1B make it difficult to draw a firm conclusion about the necessity of these promoters. The conclusions of these experiments should be moderated without further data to support that the CRISPR or CRISPRi reagents are effective.

Reviewer 3

Advance summary and potential significance to field

The authors have done a very thorough and thoughtful revision. While limitations remain, they are acknowledged and don't detract from the impact of the study as it stands.

Second revision

Author response to reviewers' comments

Once again, we thank the reviewers for their thoughtful feedback and overall support of our manuscript. Below, we list their comments in black font and our responses in blue font. We have split Reviewer 1's feedback into subsections to improve readability.

Reviewer 1:

Overall, the authors have addressed a number of the issues raised in the initial review. A concern remains with regard to the effectiveness of the CRISPR reagents and the interpretation of the data.

© 2026. Published by The Company of Biologists under the terms of the Creative Commons Attribution License (https://creativecommons.org/licenses/by/4.0/).

The authors have included CRISPR mutagenesis sequencing data which is a good addition to the manuscript.

1. However, a stronger case needs to be made or expanded for how this supports some of their conclusions. In addition, some further annotation/clarification of TableS3 would be helpful. This second issue is addressed first, as it may be relevant to the misunderstanding of the reviewer and the points made subsequently. Placing the location of the primers used to generate amplicons on the BAM file visualization would be helpful for cases like DHS15 where it is not clear why the control and the DHS15 amplicons are different sizes - is that because there were different amplicons between the control and the experimental? If so, that is fine as they capture the DHS15-g2 region, but it would help clarify why these plots look different. Another issue is that on the left side of each sheet, one guide sequence and the amplicon primers are listed. But for the conditions besides "Otx2", the constructs are indicated by the main text and the BAM stack on the right to contain two guides that target the region. Thus, it is not clear what the single "guide sequence" represents. Is it just one of the two guides or was there only one guide assessed in the experiment - the schematic would suggest not, but it is also striking that for DHS2 and Exon1B there is no evidence that one of the two guides are working (for DHS15, one guide region wasn't assessed and Exon1C does show evidence of both working).

   To address the reviewer’s comments, we tested new primers for all loci that we were unable to assess in the prior submission. This was primarily done by designing smaller amplicons. In our revised Table S3, we now show mutation data for all enhancers and promoters, though not each guide target (we were unable to get data for DHS15-g1). Additionally, we made multiple improvements to the annotations throughout each sheet in Table S3, including detailed descriptions of the BAM visualizations. Notably, each guide needed to be analyzed separately by the CrispRVariants pipeline, which is highlighted in the revised Table S3. For Exon 1C, the vast majority of reads from the CrispRVariants analysis were classified as “Other”. These are reads that do not map to the target window. This could be due to mis-primed amplicons, erroneously sequenced reads, or large deletions that failed to align. Big deletions (guide-to-guide or larger) do not appear prevalent in our BAM visualizations, suggesting that they are relatively rare. Overall, it appears that disabling mutations and whole element deletions are not the main driver of the DHS2 and DHS15 CRISPR phenotypes we observed.

2. While the inclusion of this data do add to the manuscript, it is not clear that it supports some of the conclusions. One issue brought up in the first review is the possibility that combined DHS2 and DHS15 CRISPR targeting leads to a stronger Otx2 phenotype because it deletes the Otx2 gene which is flanked by these two regions. This is an alternative explanation to the interpretation that these two regions serve a redundant function in cis-regulation of Otx2. In the TableS3 data for DHS2, there does not seem to be any evidence that g1 is working at all and so the extent with the g1/g2 combination to which the DHS2 element is disrupted beyond the immediate vicinity of g2 seems minimal. For DHS15, data was not obtained for the g1 location and g2 did not seem to have an effect. So this also does not present a clear picture that the DHS15 region is being disrupted. This raises the possibility that targeting DHS2 and DHS15 alone with guide pairs is not effective because at most only one guide is working and only causing very local changes. But in combination, DHS2 and DHS15 could lead to a large gene deletion between each of their one active guides. In the author's rebuttal it is stated that "In general, guide-to-guide deletions were rare" and from the revised manuscript text Line 305 "Importantly, we found little evidence of deletions spanning guide pairs." However, from TableS3, the only combo guide pair with evidence that both guides were working (and two active guides would be important for seeing deletions that would be predicted from a simultaneous cut at two sites and a subsequent NHEJ reaction that deletes the intervening sequence) was for Exon1C and the plots would suggest that this combination does indeed have a much higher rate of deletions aligned with each of the guide cut sites. It is not clear how these BAM plots should be read to say there is a precise guide to guide deletion (and further clarification of this in the text

© 2026. Published by The Company of Biologists under the terms of the Creative Commons Attribution License (https://creativecommons.org/licenses/by/4.0/).

would be helpful), but more than half of the reads have a sharp cutoff at one of the two guide sites on the side closest to the other guide suggesting a deletion oriented towards the other guide. Sequence data from each of the individual guides alone would inform whether the interpretation that the combined sequence data supports a deletion effect. Thus, strong evidence to rule out a DHS2 to DHS15 deletion and removal of the Otx2 coding region is not present in this sequencing data. Thus, this possibility should be noted or evidence to argue against it included.

We have expanded the annotations for BAM plots in Table S3 for improved clarity. Solid black lines running horizontally through a read denote mapped deletions. Prominent solid black lines can be seen in the BAM plots for Exon 1B. We do not see large black lines spanning entire reads in our analysis of DHS2. Moreover, the use of smaller amplicons in our new analysis makes detecting guide-to-guide deletions difficult, as PCR amplification from the internal primer would be disrupted. Such limitations are noted in our revised Table S3. Our DHS2, Exon 1A, and Exon 1B data includes amplicons that span both guides, yet there are still few guide-to-guide deletions detected. This suggests that such deletions are rare at DHS15 and Exon 1C. Thus, it is unlikely that guide-to-guide deletions between DHS2 and DHS15 that remove *Otx2* are abundant enough to account for our double targeting phenotypes. As noted below, Cas9 mediated steric hinderance of enhancer activity likely accounts for a large fraction of the observed DHS2 and DHS15 conventional CRISPR phenotypes.

3. The conclusion that Cas9 is binding to these regions and sterically inhibiting enhancer regulation could have much stronger support by using the DHS2 and DHS15 guides with a simple dead Cas9 version without any interference protein domains. That would also rule out that there is a large deletion being created with DHS2/15 simultaneous CRISPR.

This is a great idea! We built dCas9-only (lacking KRAB and MeCP2 domains) plasmids targeting either DHS2 or DHS15 containing the same guides used in the CRISPR experiments. These plasmids were electroporated into P0 retinal explants and collected after three days of culture, similar to the experiments in Figure 3. The results are shown in a new Fig. S11, along with modest additional text in the results, methods, and discussion sections. We observed that the dCas9-only plasmids strongly reduced OTX2 expression after three days, though not as well as conventional (Cas9) plasmids. This argues that Cas9 steric hinderance disables DHS2 and DHS15 enhancer function. As noted above and in our revised results and discussion sections, we were unable to profile all of the mutations caused by conventional CRISPR. We are probably missing at least a third of possible mutations at any given locus for various technical reasons. Thus, we conclude that mutations and steric hinderance combine to disable DHS2 and DHS15 function. This accounts for the OTX2 expression loss we observed in our conventional CRISPR experiments. The readily observed steric hinderance also argues against the likelihood of very large guide-to-guide deletions between DHS2 and DHS15 driving phenotypes.

4. For the investigation of the promoter usage of Otx2, the lack of sequencing data for exon 1A and a lack of efficacy of the guides targeting exon 1B make it difficult to draw a firm conclusion about the necessity of these promoters. The conclusions of these experiments should be moderated without further data to support that the CRISPR or CRISPRi reagents are effective.

As noted above, we were able to profile the mutations at all three promoter regions. In general, most mutations were single nucleotide variants and small indels. However, Exon 1B had a small population of guide-to-guide deletions. The relatively low number of disabling mutations are unlikely to significantly disrupt the promoters. While CRISPRi had only modest phenotypes, it is possible that it is not effective at the targeted locations.

© 2026. Published by The Company of Biologists under the terms of the Creative Commons Attribution License (https://creativecommons.org/licenses/by/4.0/).

Thus, it is difficult to know whether any promoter is necessary for *Otx2* expression without extensive new experimentation (*e.g.*, promoter knock-out mice). As such, we have softened our conclusions throughout the manuscript about promoter necessity.

Reviewer 3:

The authors have done a very thorough and thoughtful revision. While limitations remain, they are acknowledged and don't detract from the impact of the study as it stands.

Thank you. We hope the revisions described above further strengthen the manuscript.

Third decision letter

MS ID#: dev.204881R2

MS TITLE: A robust cis-regulatory network ensures Otx2 expression during retinal development

AUTHORS: Ian Purvis, Omar Ochoa Olmos, Ko Park, Michael Kaufman, Charles Henry, Christopher Schaaf, Olivia Clise, Corey Tesdahl, Ami Haas and Joseph A. Brzezinski IV

Dear Dr Brzezinski IV,

I am delighted to tell you that your manuscript has been accepted for publication in Development, pending our standard publication integrity checks.

© 2026. Published by The Company of Biologists under the terms of the Creative Commons Attribution License (https://creativecommons.org/licenses/by/4.0/).
